# The effectiveness of telerehabilitation in upper limb musculoskeletal disorders: a systematic review

**DOI:** 10.1186/s12891-026-10008-7

**Published:** 2026-05-28

**Authors:** Charlotte Pietzsch, Maria Girbig, David Reissig, Susanne Titze, Alice Freiberg, Albert Nienhaus, Andreas Seidler

**Affiliations:** 1 Faculty of Medicine, Institute and Policlinic of Occupational and Social Medicine (IPAS), TU Dresden, Dresden, Germany; 2 German Social Accident Insurance for the Health and Welfare Services (BGW), Hamburg, Germany

**Keywords:** Telerehabilitation, Telemedicine, Remote rehabilitation, Musculoskeletal, Upper limb, Exercise therapy, Physical therapy, Occupational therapy, Systematic review, Meta-analysis

## Abstract

**Background:**

Telerehabilitative services have been increasingly used in recent years, raising questions about their effectiveness. In musculoskeletal disorders, evidence supports telerehabilitation but methodological quality of evidence is limited and focused on lower limb disorders. Hence, this systematic review aimed to assess the effectiveness of telerehabilitation in upper limb musculoskeletal disorders.

**Methods:**

We conducted electronic searches in MEDLINE, Embase, and AMED (up until 11/27/2024), complemented by hand searches. Randomized (RCTs) and non-randomized studies of interventions (NRSIs) assessing upper limb musculoskeletal disorders reporting on pain, activities of daily living (ADL), or health-related quality of life (HrQol) were included. We focused on studies from high-income countries in English or German. Risk of bias was assessed using RoB 2 and ROBINS-I. Standardized mean differences (SMDs) and 95% confidence intervals were derived from meta-analysis, where negative values favor telerehabilitation. Certainty of evidence was rated using GRADE. We grouped studies comparing telerehabilitation to standard care (subgrouped into in-person or minimal intervention) and separately examined studies with a tele-adjunct. Compared to in-person rehabilitation we assessed whether outcomes were similarly improved with telerehabilitation. For tele-interventions as adjunct or compared to minimal care we assessed whether telerehabilitation improved outcomes.

**Results:**

We included twenty RCTs representing 1,285 participants. All NRSIs were excluded due to critically high risk of bias. Compared to in-person rehabilitation, telerehabilitation showed similar improvements with very low certainty of evidence in pain (SMD: -0.33 [-1.16 to 0.5] VAS/NRS), moderate in ADL (SMD: -0.07 [-0.31 to 0.17] QuickDASH/DASH), and low in HrQol (SMD: -0.26 [-0.82 to 0.31]). Compared to brochure-based exercising, certainty of evidence was low for improved pain (SMD: -0.28 [-0.58 to 0.03]), moderate in ADL (SMD: -0.56 [-0.88 to -0.24]), and low in HrQol (SMD: -0.31 [-0.65 to 0.02]). Tele-adjunct yielded low certainty of evidence for improvements in pain (SMD: -0.38 [-0.71 to -0.05]) and moderate in ADL (SMD: -0.51 [-0.88 to -0.14).

**Conclusions:**

Our results tend to support the effectiveness of telerehabilitation across different comparisons although certainty of evidence is inconclusive. Future studies should particularly investigate the non-inferiority of telerehabilitation compared to in-person rehabilitation and the added benefit of tele-adjunct.

**Trial registration:**

Prospero registration ID: CRD42024622465

**Supplementary Information:**

The online version contains supplementary material available at 10.1186/s12891-026-10008-7.

## Background

Musculoskeletal disorders (MSDs) are highly prevalent with 1.71 billion people affected in 2019 worldwide. The impact on burden of disease within these conditions is reflected in the high number of years lived with disability (YLDs) [1]. MSDs are predominantly contributing to the number of people who would benefit from rehabilitation. This is particularly observed in the age group from 15 to 64 years, which corresponds to the working age, and where MSDs account for about two-thirds of the number of people presumably in need of rehabilitation [1]. This suggests a high importance of musculoskeletal disorders and their rehabilitation on the working forces. Moreover, musculoskeletal disorders have been found to be the leading cause for all work-related complaints, with three out of five workers affected in Europe (i.e., EU-28 states) [2]. MSDs often result in absence from work, especially of longer time-periods. For Germany, the economic impact with loss of production and loss of gross value added has been estimated with 0.5 to 1.0% of the gross domestic product [2].

Although MSDs are mainly attributable to back disorders, the upper limb plays an important role in the working context [1, 2]. 41% of workers report on shoulder, neck or upper limb complaints [2]. Furthermore, the upper limb is a common site of occupational injury [3, 4]. Additionally, occupational diseases due to mechanical overload of the upper limb, such as rotator cuff disorders and carpal tunnel syndrome, represent a substantial burden [5]. In German statutory insurance system, the latter is recognized as one of the nine most frequent occupational diseases [5].

Although the need for rehabilitation is substantial, it often remains unmet due to several barriers, including limited service availability in rural areas, long waiting lists, and underutilization of existing services [6, 7]. Telerehabilitation offers a promising solution by improving access to rehabilitation services and expanding service capacity [8]. Telerehabilitation is a subconcept of telemedicine, defined as the “delivery of therapeutic rehabilitation at a distance or offsite using telecommunication technologies” [9, 10]. This definition suggests a multifaceted concept, encompassing therapeutic rehabilitation that may include education, physical exercising, or psychologic interventions [11]. Communication technologies may range from simple text messaging to complex delivery pathways involving sensory systems [8, 11, 12]. Distinction is made between synchronous modes (i.e., real-time interaction for example over videoconferencing sessions) and asynchronous modes (i.e., sessions are performed independently by the patient for example with prerecorded digital training sessions) [8]. Due to technological advance, usage of telemedicine has expanded gradually, with the COVID-19 pandemic further accelerating this trend [13–16]. In addition to contributing to the general preparedness of the healthcare system, telerehabilitation may increase access to rehabilitation services, for example in rural communities [8, 17, 18]. It may also compensate personnel shortages, reduce costs, and carbon emission [17, 19, 20]. However, technical issues can negatively impact the usage of telerehabilitation and the digital divide (i.e., systematic differences in access to, experience with, skills to use, and opportunities to use technology, see [21]) may exacerbate health inequalities [14, 22, 23]. Systematic reviews on musculoskeletal disorders generally support the effectiveness of telerehabilitation, but quality of the evidence is limited, and foremost based on investigations of the lower limb or back disorders [8, 11]. Furthermore, telerehabilitation effectiveness appears to be context-dependent, with various implementation approaches possible. Telerehabilitation can serve either as an alternative to usual care (e.g. in-person rehabilitation or other) or as an adjunct to usual care. If used as an alternative to an in-person rehabilitation, telerehabilitation can be understood as a delivery mode transporting the same content as face-to face therapy but over a distance, e.g. therapy sessions over videoconferencing. In this case, interest is directed to whether this delivery mode leads to outcomes comparable to an in-person care (e.g. non-inferiority of telerehabilitation). Whereas in other implementation settings, the telerehabilitation intervention provides additional support exceeding usual care, e.g. guided home exercise programs supported by telerehabilitation, where usual care consists of a short instruction to self-exercising. Accordingly, studies examine the non-inferiority or superiority of telerehabilitation interventions.

Within this systematic review, we sought to synthesize evidence on telerehabilitation that can be transferred to rehabilitation of work-related disorders. To this end, we focused on upper limb musculoskeletal disorders given their strong link with the working context. Unlike prior reviews [24], we did not restrict the review to a specific diagnosis but included a broader spectrum of upper limb disorders considered to be associated with the working context. Our aim was to provide a robust evidence synthesis in the field which considers different implementation modes. Hence, we sought to estimate effect sizes that can be expected when telerehabilitation is used as an alternative to an in-person rehabilitation, as a replacement for minimal care (e.g., advice, paper-based instructions) or when it is used as an adjunct to standard care. Accordingly, the following research questions were examined:

1. Does telerehabilitation for musculoskeletal disorders of the upper limb lead to similar improvements compared to in-person rehabilitation regarding pain, activities of daily living (ADL), and health-related quality of life (HrQol)?
2. Does telerehabilitation for musculoskeletal disorders of the upper limb improve pain, ADL, and HrQol compared to a minimal form of standard care?
3. Does telerehabilitation as an adjunct to standard care lead to improved pain, ADL, and HrQol compared to standard care only/or with minimal add-on?

## Methods

The review methods were guided by the Cochrane handbook [25] and reported according to the PRISMA statement [26] (see Additional file 1). The protocol for this systematic review was registered on PROSPERO database (CRD42024622465). During review conduct, we deviated from the protocol in three respects: First, we lowered the minimum age to 16 years, as apprenticeships can begin at this age, making work-related disorders relevant to this population. Second, we grouped studies comparing telerehabilitation to standard care, with subgrouping into standard care as in-person rehabilitation or minimal forms of standard care. Third, studies using a tele-intervention as adjunct to standard care were analyzed separately.

### Literature search

We searched the electronic databases MEDLINE, Embase, and AMED up to 11/27/2024 (for search strings of electronic databases see Additional file 2). Additional searches were conducted on Clinical Trials.gov and rehadat.de, as well as in reference lists of included studies and related systematic reviews, and by forward citation tracking of included studies via Google Scholar.

### Inclusion and exclusion criteria

Following the PICOS (Patient, Intervention, Comparison, Outcome, Study design) [27] scheme, we included studies involving adults aged 16 and older with chronic or traumatic musculoskeletal disorders or neural compression syndromes of the upper limb. Studies with mixed diagnoses were considered only if they reported separate results for each included diagnosis. We excluded studies with populations presenting underlying systemic diseases (e.g., fibromyalgia, rheumatoid arthritis, neurologic, or oncologic disease) or including persons under 16 years of age (Population). The intervention had to be delivered remotely via any telecommunication mode and contain at least one of the following components: exercise, education, advice, coaching, or other psychological interventions aiming at self-management of symptoms (Intervention). Studies comparing telerehabilitation to no rehabilitation or to standard care (either as an in-person rehabilitation or a minimal form of rehabilitation) were included (Comparison). The primary endpoints were pain, ADL, and HrQol (Outcome). Studies that examined the following secondary endpoints were also included: range of motion, strength, return-to work, self-efficacy, cost-effectiveness, adverse events, resource-usage, patient satisfaction, and intervention adherence. However, these are not subject of this report. For study design, randomized controlled trials (RCTs) were included, as they provide the most robust evidence for effectiveness. Non-randomized studies of interventions (NRSIs) with a control group were also considered, as they reflect the pragmatic study designs often used in digital interventions [28]. Additional criteria included publications in English or German language, dated from January 2000 onwards, and originating from high-income countries, as defined by the World Bank [29]. For this classification the gross national income (GNI) per capita is calculated and expressed in U.S. dollars. Based on absolute thresholds, economies are assigned to one of four income groups: low, lower-middle, upper-middle, and high [30]. We excluded studies carried out in middle- and low-income countries due to their pronounced barriers to telemedicine, which are often characterized by limited infrastructure, resources, and knowledge, which may result in more selective study populations in these countries [31].

### Selection, data extraction & risk of bias assessment

After removing duplicates, titles and abstracts as well as subsequent full texts were screened for eligibility. Two reviewers worked independently in title/abstract (CP, ST) and full-text screenings (CP, DR), with a preceding pilot phase. Conflicts were resolved through consent meetings, and in cases of persistent dissent, a third (MG) and fourth (AS) reviewer was consulted. Data extraction was performed by one reviewer (CP), with a second reviewer providing quality control (DR). Covidence was used for screening and data extraction. In case of missing data, authors of studies were contacted. Data were collected from all reported measures within one of the outcome domains at three time points: at the end of the intervention, six months post-intervention, and one-year post-intervention. Since intervention duration varied across studies, the first time-point - defined as the end of the intervention - differed between studies. For the second time-point, data from any time-point later than the end of intervention measurement up to six months after end of the intervention was used (if more than one time-point was available the one closest to six months post- intervention was selected). Similarly, data for the third time-point were collected from assessments conducted more than six months and up to 12 months after the end of intervention. Additional data were collected on study design, country, data collection period, setting, participants, sample size, methods, funding, conflicts of interest, details of intervention, and control intervention. Two reviewers (CP, DR) independently assessed the risk of bias using the RoB 2 tool for RCTs and the ROBINS-I V2 tool for NRSIs [32, 33]. Results with a critical risk of bias (i.e. the study is very problematic and should be excluded from synthesis) within ROBINS-I assessment were excluded [32, 34]. The results of the RoB 2 assessment were visualized using robvis [35]. Within RoB 2, results are assessed in five domains, with levels low, some concerns, and high risk of bias, from which an overall risk of bias judgement can be derived. Consensus was reached through subsequent meetings (among CP, DR), and in cases of discrepancy, two additional reviewers were consulted (MG, AS).

### Qualitative and quantitative synthesis

For synthesis, we prioritized endpoint measures of metric data, which were used to calculate standardized mean differences (SMDs) and 95% confidence intervals (CIs) as the standardized metric. To facilitate interpretation, we standardized all SMDs so that negative values favored telerehabilitation and positive values favored the control intervention, regardless of the underlying measurement instrument. An SMD of 0.2 to < 0.5 was considered small, 0.5 to < 0.8 medium, and 0.8 or higher a large effect [36]. When studies reported median differences due to skewed data, we did not undertake transformation, and data were only included into narrative synthesis. For better clinical interpretation, SMDs reported within the summary of findings table were back-transferred into the original instrument’s metric. To this end a pooled SD was calculated and SMD [95% CI] was multiplied by it [37, 38]. Results were synthesized within each outcome domain, with the primary focus on the time point immediately following the intervention. Results from later time points were collected as described above and are reported in Additional file 7. Results were analyzed in two groups: (1) Telerehabilitation was compared to standard care, with subgrouping into in-person rehabilitation or minimal intervention. Herein, the overall pooled estimate is not reported because of deviating hypotheses. (2) Tele-interventions as adjunct to standard care were compared to no/minimal adjunct. Quantitative synthesis was executed if two or more studies within the aforementioned grouping system provided data from the same scale or instrument (in pain, VAS and NRS scales were treated as one instrument, likewise DASH and QuickDASH in ADL). The data were synthesized by calculating a pooled SMD using random effects model, since we expected varying effects due to difference in study settings and study populations, and analyses were visualized with forest plots. Statistical heterogeneity was assessed using I² and the p-value for heterogeneity. Subgroup analyses were initially planned for risk of bias as well as for patient and intervention specific variables (e.g. gender, age, delivery mode). If applicable, sensitivity analysis was performed by excluding highly problematic studies from analysis, i.e., studies with some concerns or high risk of bias in at least three domains. When combining endpoint data and change data within one SMD-analysis, we used an imputed endpoint standard deviation instead of standard deviations from change data as proposed within the Cochrane Handbook [39]. For publication bias, funnel plots were examined and the Egger`s test was conducted with a minimum of ten studies [40]. All analyses were executed using the metafor package in R [41, 42].

### GRADE – assessment of the certainty of evidence

Certainty of evidence was rated using GRADE methodology by one reviewer (CP) for the results listed in Table 2 [43]. Since only results from RCTs were ultimately judged, certainty of evidence rating started with high and downgrade was undertaken within five domains: risk of bias, inconsistency, indirectness, imprecision, publication bias. Comparing telerehabilitation to in-person rehabilitation, we assessed the certainty of evidence that outcomes are improved to a similar extent. The margin was set to an SMD of 0.2 in favor of in-person rehabilitation (with positive values favor in-person rehabilitation). If the upper limit of the confidence interval exceeded 0.2, it was concluded that outcomes may be more improved with in-person rehabilitation and downgrade for imprecision was applied. This threshold can be considered as conservative, for an SMD of 0.2 corresponds to a small effect and does not correspond necessarily with an effect of clinically meaningfulness. For comparison to minimal standard care and telerehabilitation as an adjunct, we rated the certainty of evidence, that telerehabilitation leads to improved outcomes compared to the control intervention. To this end, the margin for downgrade of imprecision was set to 0. That means that results were only downgraded for imprecision if the confidence interval also included a possible negative effect for telerehabilitation. Conversely, results were not downgraded if the confidence interval indicated a beneficial effect for telerehabilitation, even if a negligible positive effect was also included.

## Results

### Search results

**Fig. 1:**
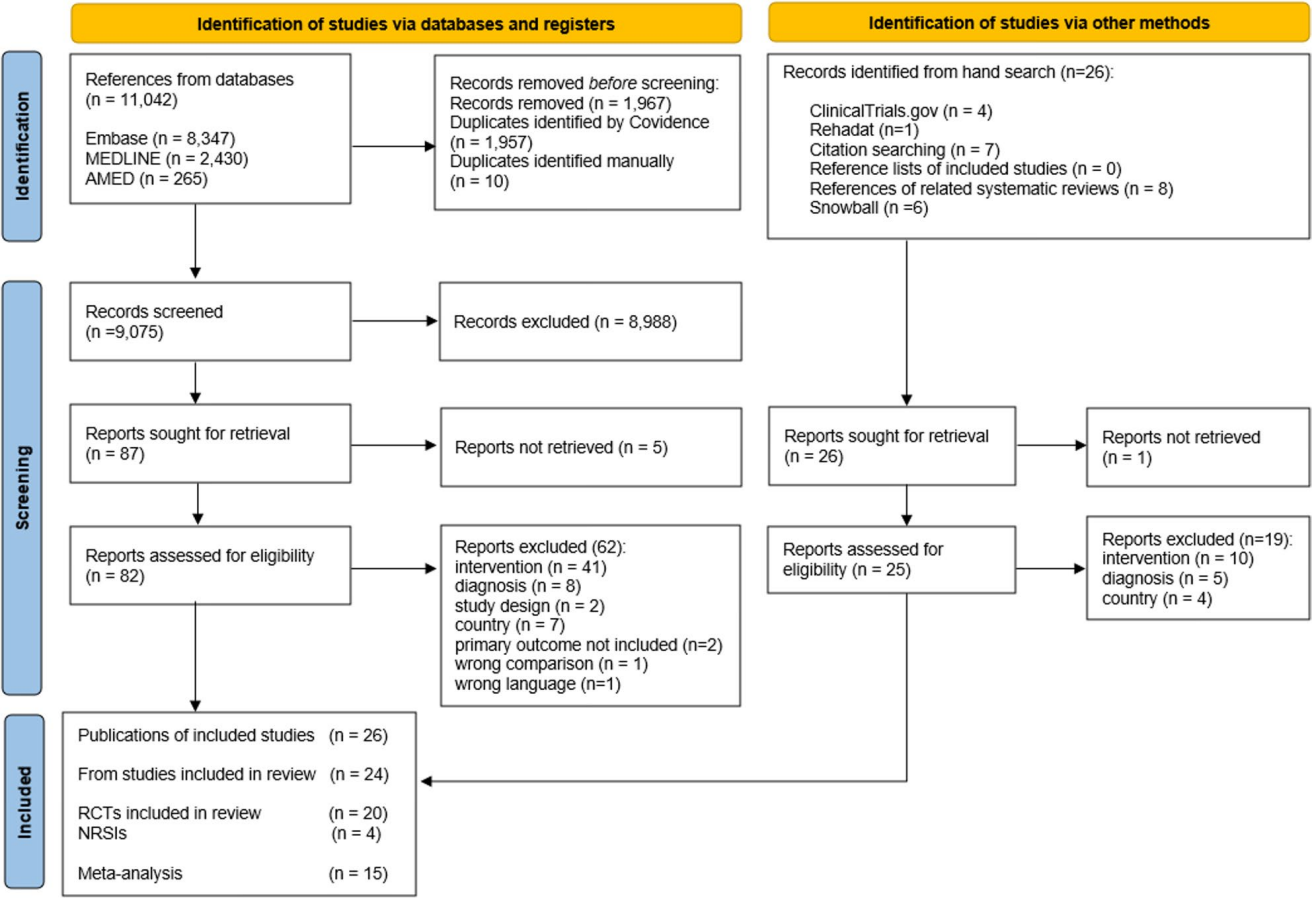
PRISMA flow chart of included studies

We initially retrieved 11,042 articles through the electronic database searches. After removing duplicates 9,075 publications remained. Additionally, 26 references were identified through supplementary search methods. Studies excluded during full-text screening are listed in Additional file 3 with reasons for exclusion. Finally, we were able to include 20 RCTs and four NRSIs (see PRISMA flow chart in Fig. 1). Although initially included, the four NRSIs were excluded from synthesis in the further course due to critical risk of bias as assessed using the ROBINS-I V2 tool (see Additional file 4 for ROBINS-I results of NRSI studies). Therefore, the following section presents study characteristics for RCTs only. The results from NRSI studies were not included into any synthesis (i.e., qualitative and quantitative synthesis) and were not considered within the discussion part. This process follows recommendation of the Cochrane methods group [34]. For completeness, the characteristics of excluded NRSIs are presented in Additional file 8.

### Main characteristics of the studies

Main characteristics of the RCTs are depicted in Table 1 and in Additional file 5. Twenty RCTs (from 21 reports) representing 1,285 participants were included into synthesis: Sixteen trials compared telerehabilitation to standard care [44–60]. Of these, 11 trials used in-person rehabilitation as the control intervention [44, 47–51, 53, 55, 56, 59, 60]. In the remaining five trials, telerehabilitation was compared with minimal care, which always consisted of a brochure-based instruction to home exercising, sometimes with one-time instruction or occasional monitoring [45, 46, 52, 57]. In four other trials, telerehabilitation was incorporated as an add-on to standard care [12, 61–63].

**Table 1:** Characteristics of included studies

Study, year (Country)	Diagnosis N: total randomized (IG/CG)	Intervention (IG)	Control group (CG)	Outcome (Instrument)	SMD [95% CI], negative values favor telerehabilitation^a^	Overall risk of bias^b^
Telerehabilitation versus standard care (subgroup = in-person rehabilitation)						
Barrett et al., 2024 [44] (USA)	Primary thumb CMC arthroplasty N: 67 (31/36)	Video-based instruction to home exercise protocol	In-person instruction to home exercise protocol	ADL (PROMIS UE)	-0.12 [-0.63 to 0.39]	High
Correia et al., 2022 [48] (Portugal)	Rotator cuff repair N: 50 (27/23)	Guided exercise program using tablet app. Weekly remote adjustment by therapist + home-based in-person sessions.	Home-based in-person rehabilitation sessions.	Pain (CMS pain subscale), 0–15, higher is better	Median Difference [95% CI]: 0 [− 5 to 5]	Some concerns
				ADL (QuickDASH)	0.12 [-0.56 to 0.80]	
				ADL (CMS)	0.16 [-0.51 to 0.84]	
Coughlin et al., 2021 [49] (United Kingdom)	Distal radius fractures, conservative treatment N: 80 (40/40)	Video instruction to self-directed exercise	In-person instruction to self-guided exercising	ADL (Change in DASH)	0.07 [-0.41 to 0.55]	Some concerns
Lara et al., 2022 [50] (USA)	Distal radius fracture, operative treatment N: 51 (22/29)	Video instruction to self-directed exercise	In-person instruction to self-guided exercising	Pain (VAS)	0 [-0.57 to 0.57]	Some concerns
				ADL (QuickDASH)	-0.40 [-0.96 to 0.16]	
				HrQol (VR-12 physical scale)	-0.26 [-0.83 to 0.31]	
Pastora-Bernal et al., 2018a [54] & 2018b [55] (Spain)	Subacromial impingement syndrome, operative treatment N: 18 (8/10)	Customized exercises program through web application, complemented by videoconferencing with therapist	In-person physical therapy (manual therapy, home exercise programs and other physiotherapy techniques)	Pain (CMS pain subscale)	-0.61 [-1.56 to 0.34]	Some concerns
				ADL (CMS total):	0.59 [-0.32 to 1.49]	
Tousignant et al., 2020 [59] (Canada)	Proximal humerus fracture, conservative treatment N (randomized): 31 N (analyzed): 30 (15/15)	Supervised exercise sessions and advice via teleconferencing + unsupervised exercise	Supervised exercise sessions in-person + unsupervised exercise	pain (CMS pain subscale)	-0.23 [-0.95 to 0.48]	Some concerns
				ADL (CMS total)	-0.23 [-0.92 to 0.47]	
				ADL (DASH)	-0.09 [-0.78 to 0.61]	
Vasavada et al., 2024 [60] (USA)	Rotator cuff repair N: 32 (12/20)	Home exercise program through internet-based telerehabilitation software, videoconferencing with therapist.	In-person physical therapy	ADL (ASES Score)	(only visual): improved ASES scores in both groups at 6 months with higher values in IG. Vast cross-over from IG to CG	High
Pak et al., 2023 [53] (USA)	Tendon related shoulder pain N: 90 (46/44)	Fully remote, tailored program including home exercise, education, cognitive behavioral therapy via web-app using motion sensors	Supervised exercise, manual therapy, education, motivational interviewing and cognitive behavioral therapy if appropriate	Pain (NRS), 0–10	Median difference [95% CI], CG-IG (positive values favor telerehabilitation): -0.6 [-0.9 to -0.4]	Some concerns
				ADL (QuickDASH), 0-100	Median difference [95% CI], CG-IG (positive values favor telerehabilitation): -2.3 [-14.7 to 10.0]	
Marley et al., 2022 [51] (United Kingdom)	Shoulder impingement syndrome, operative treatment N: 64 (31/33)	Software-based exercise program including exergaming, tailored to patients’ ability, weekly remote review of progress	Weekly physical therapy with assessment for progression and provision of standardized home exercise program.	ADL (DASH)	0.23 [-0.27 to 0.72]	Some concerns
				ADL (OSS)	0.17 [-0.33 to 0.66]	
				HrQol (EQ-VAS)	Only p-value provided: no significant difference in the EQ-VAS score in either group at any time point (*p* = 0.587)	
Choi et al., 2024 [47] (Republic of Korea)	Distal radius fracture, operative treatment N: 36 (21/15)	home-based exercise with smart glove	In-person rehabilitation + home exercise self-directed	Pain (VAS)	-0.88 [-1.98 to 0.22]	High
				ADL (QuickDASH)	-0.72 [-1.73 to 0.29]	
				ADL (Mayo Score)	-0.36 [-1.35 to 0.63]	
Roddey et al., 2002 [56] (USA)	Rotator cuff repair N: 108 (54/54)	Video instruction to self-directed exercise	In-person instruction to self-guided exercising	ADL (SPADI)	0.18 [-0.23 to 0.59]	High
				ADL (Penn Score)	0.01 [-0.40 to 0.42]	
Telerehabilitation versus standard care (subgroup = minimal rehabilitation)						
Blanquero et al., 2019 [45] (Spain)	Carpal tunnel syndrome, operative treatment N: 50 (25/25)	Tailored home exercise program using tablet app	Paper-based instruction to self-guided home exercise	Pain (VAS)	-0.33 [-0.89 to 0.23]	Some concerns
				ADL (QuickDASH)	-0.57 [-1.13 to -0.02]	
Blasco et al., 2024 [46] (Spain)	Reverse shoulder arthroplasty N: 31(17/14)	Rehabilitation program assisted with a chatbot: information, exercise instructions, diary, weekly feedback	Same rehabilitation protocol brochure-based + one in-person session	Pain (NRS)	-0.17 [-0.88 to 0.54]	Some concerns
				ADL (QuickDASH)	-1.33 [-2.09 to -0.57]	
				ADL (CMS)	-0.36 [-1.05 to 0.34]	
				HrQol (EQ-5D-5 L)	0.00 [0.71 to -0.71]	
Meijer et al., 2024 [52] (Netherlands)	Distal radius fracture, operative or conservative treatment N: 93 (47/46)	Smartphone or tablet-based exergaming	Home-based unsupervised wrist exercises	Pain (NRS)	only reported visually: at week 6 endpoint values for both groups are close to each other, confidence intervals overlapping	Some concerns
				ADL (Change in PRWE):	0.16 [-0.24 to 0.57]	
Rodríquez-Sánchez-Laulhé et al., 2023 [57] (Spain)	Hand osteoarthritis N Participants: 74 (34/40) N hands: 144 (66/78)	Home exercise with tablet app: diary, advice, exercise instructions, symptom report	Paper-based home exercise program. Introductory in-person session.	Pain (change in NRS)	-0.59 [-0.95 to -0.23]	High
				pain (change in AUSCAN)	-0.18 [-0.54 to 0.17]	
				ADL (Change in QuickDASH)	-0.42 [-0.78 to -0.06]	
				ADL (Change in AUSCAN)	-0.21 [-0.56 to 0.15]	
Shim et al., 2023 [58] (Republic of Korea)	Rotator cuff tear N: 115 (58/57)	Brochure-based exercise followed by AR-based exercises for 6 weeks with performance feedback, two monitoring sessions	Brochure-based home exercises + diary; condition checked by weekly telephone call.	Pain (NRS)	0.02 [-0.36 to 0.39]	High
				ADL (SPADI)	-0.07 [-0.44 to 0.31]	
				ADL (DASH)	-0.27 [-0.65 to 0.10]	
				ADL (SST)	-0.36 [-0.74 to 0.01]	
				HrQol (EQ-5D-5 L)	-0.40 [-0.78 to -0.02]	
Telerehabilitation as add-on to standard care versus no/minimal add-on						
Blanquero et al., 2020 [62] (Spain)	Work-related hand or finger injury N: 74 (40/34)	In-person sessions same in both groups, App-based home exercise program + weekly monitoring with therapist	In-person sessions same in both groups, paper-based home exercise program, weekly monitoring during in-person session	Pain (VAS)	-0.47 [-1.08 to 0.14]	High
				ADL (QuickDASH)	-0.57 [-1.17 to 0.03]	
Chen et al., 2017 [12] (Taiwan)	Frozen shoulder, conservative treatment N: 66 (33/33)	Instruction to perform daily exercises + daily text messaging (reminders, encouragement, education)	Instruction to perform daily exercises	Pain (VAS)	-0.08 [-0.59 to 0.42]	High
				ADL (SST)	0.04 [-0.46 to 0.54]	
Choi et al., 2019 [63] (Republic of Korea)	Frozen shoulder, conservative treatment N: 84 (42/42)	NSAIDs + education to self-guided exercise, app-based support of exercising	NSAIDs + education to self-guided exercise	Pain (VAS)	-0.19 [-0.61 to 0.24]	High
Martinez-Rico et al., 2018 [61] (Spain)	Shoulder instability with Bankart repair N: 71 (36/35)	Out-patient physical therapy with home exercise program + phone-based coaching to self-care	Out-patient physical therapy with home exercise program	Pain (VAS):	-0.81 [-1.30 to -0.33]	High
				ADL (DASH):	-0.47 [-0.94 to 0.00]	
				ADL (OSIS):	-0.40 [-0.87 to 0.07]	

*SMD* Standardized mean difference, *CI* confidence interval, *IG* intervention group, *CG* control group, *CMC* carpometacarpal, *ADL* activities of daily living, *HrQol* health-related quality of life, *NSAID* non-steroidal anti-inflammatory drug, *VAS* visual analogue scale, *NRS* numeric rating scale, *CMS* Constant-Murley Score, *DASH* Disabilities of the Arm, Shoulder and Hand questionnaire, *VR-12* Veterans RAND 12-item health survey, *EQ-5D-5 L* EuroQol five dimensions five levels measurement, *OSIS* Oxford Shoulder Instability Score, *SST* Simple Shoulder test, *PROMIS UE* patient reported outcomes measurement information system upper extremity, *OSS* Oxford shoulder score, *EQ-VAS* EuroQol visual analogue scale, *ASES score* American Shoulder and Elbow Surgeons Score, *Mayo* modified Green-O’Brien score, *SPADI* Shoulder Pain And Disability Index, *Penn score* Pennsylvania shoulder score, *PRWE* Patient-Rated Wrist Evaluation, *AUSCAN* Australian/Canadian Osteoarthritis Hand Index

^a^if no SMD could be derived, data are provided as reported in primary study accompanied by context information for interpretation

^b^risk of bias was assessed at result level, not study level, but because there was no deviation of assessment within several results of one study, the overall assessment is reported here at study level. See Additional file 4 for individual result assessment

The studies originated from the countries Spain [45, 46, 55, 57, 61, 62], USA [44, 50, 53, 56, 60], Republic of Korea [47, 58, 63], United Kingdom [49, 51], Canada [59], the Netherlands [52], Portugal [48], and Taiwan [12]. Twelve studies investigated shoulder disorders with the following diagnoses/procedures: rotator cuff tear [48, 56, 58, 60], subacromial impingement [51, 55], frozen shoulder [12, 63], proximal humerus fracture [59], tendon related shoulder pain [53], reverse shoulder arthroplasty [46], and Bankart lesion [61]. Eight trials examined disorders of the wrist or the hand with diagnoses/procedures: distal radius fracture [47, 49, 50, 52], carpal tunnel syndrome [45], hand osteoarthritis [57], thumb arthroplasty [44], and work-related hand or finger injuries [62]. Most studies focused on patients undergoing rehabilitation after an operative procedure [44–48, 50–52, 55, 56, 58, 59, 61, 62], with only six studies including patients following conservative management [12, 49, 52, 53, 57, 63]. The mean age of participants ranged from 28 to 70 years, with an average of 56 years. A higher percentage of females (64%) was observed. Further, we noted that trials often excluded participants with language barriers or insufficient digital access.

Most participants received a tele-intervention, where they exercised with the help of an application run on a smartphone, tablet, or personal computer. The intervention always included an exercise component, except for the study by Martinez-Rico et al. [29], which tested a coaching intervention, and the study by Chen et al. [12], where messages with reminders and information were sent. Exercise interventions were supplemented with educational content in three trials [53, 57, 59] and in one trial with a cognitive behavioral therapy element [53]. Seven trials included monitoring of training activities or symptoms [46, 48, 51, 53, 57, 58, 62]. Eleven studies used an individualized tele-intervention [45, 47, 48, 51–54, 57, 59, 61, 62].

Telerehabilitation often appeared in asynchronous mode like exercise applications via smartphone, tablet or personal computer [45–48, 51–53, 55, 57, 58, 60, 62, 63], with four studies implementing exergaming or augmented reality [47, 51, 52, 58]. In four other trials, telerehabilitation consisted of digital video material provision, containing exercise instructions [44, 49, 50, 56], which can also be described as asynchronous. Video or telephone conferencing, which are synchronous delivery modes, were dominantly used as a complementary element [45, 53, 55, 60, 62] and only twice as the main component [59, 61]. Only one study used a mixed form of telerehabilitation by combining tele-sessions with in-person sessions [48].

### Risk of bias

**Fig. 2:**
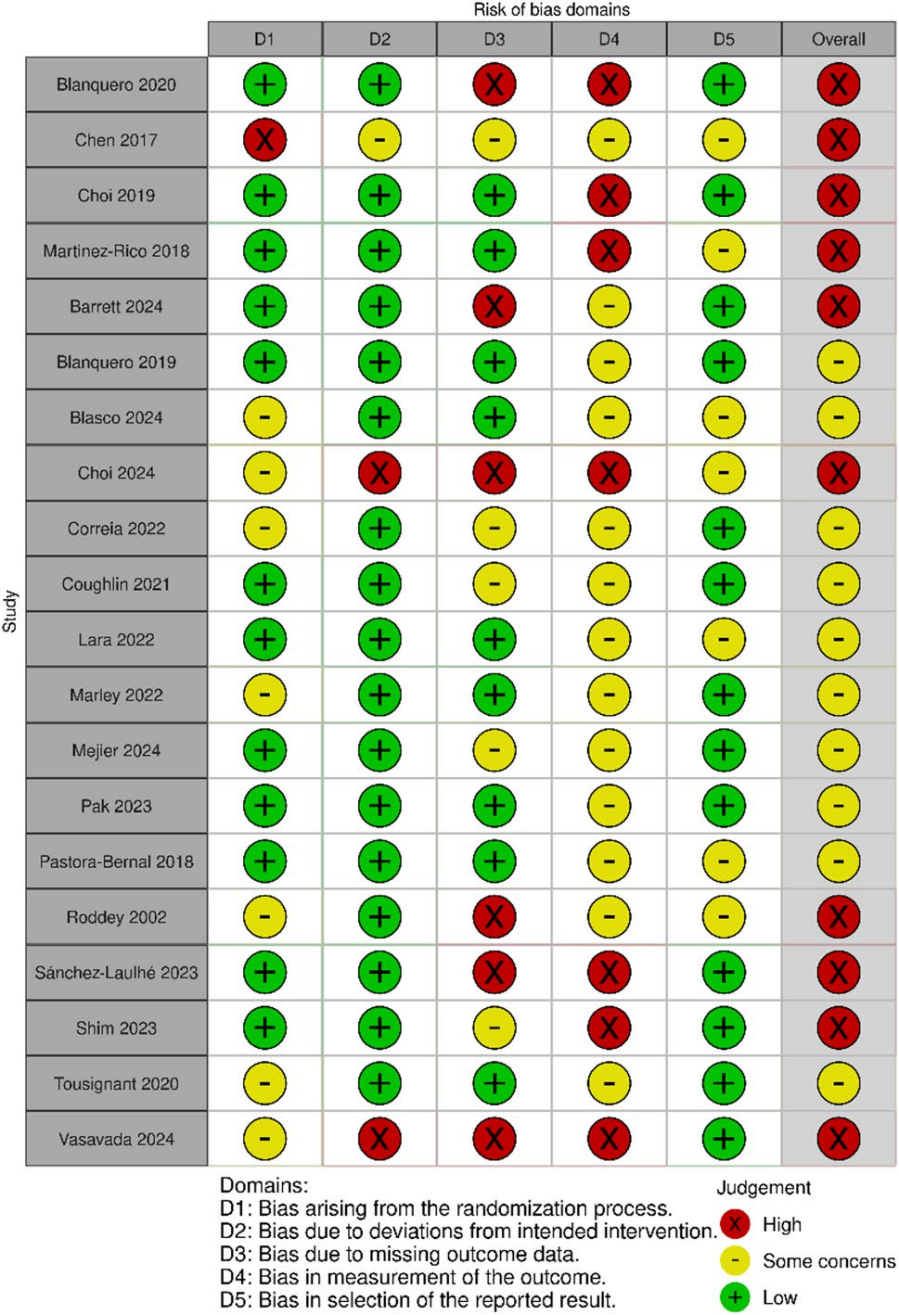
Results of RoB 2 assessment on study level

As reported above, all four NRSIs were excluded due to critical risk of bias within ROBINS-I assessment (See Additional file 4). During RoB 2 assessment, ten RCTs were judged to have some concerns regarding risk of bias, and another ten were judged to be at high risk of bias. Figure 2 depicts the risk of bias in the individual RCTs (as assessments did not differ between outcomes within a given study, RoB 2 judgements are presented at the study level; see Additional File 4 for individual RoB 2 ratings).

All studies received a judgement of at least some concerns in domain four due to the impossibility of blinding in combination with subjective outcome measures. Additionally, missing outcome data and missing study protocols were commonly issued and sometimes concealment of allocation was not reported in sufficient detail. Three studies involved industrial funding [48, 52, 53] and in six studies, conflicts of interest were issued with the authors partially being involved in the company marketing of the tested software product [45, 48, 53, 55, 57, 62].

### Results for pain, ADL and HrQol

The results of individual studies for effect directly after the intervention are presented in standardized metric in Table 1 (for group values see Additional file 5). Findings of later follow-up measurements are presented in Additional file 7.

#### Pain

Fifteen studies reported on pain [12, 45–48, 50, 52, 53, 55, 57–59, 61–63]. In six studies comparing telerehabilitation to in-person care [47, 48, 50, 53, 54, 59], the average SMDs in pain ranged from − 0.88 to 0.00. These results indicate no difference between groups (i.e. a mean effect size under 0.2, which was not statistically significant) or a tendency for telerehabilitation to be more effective (i.e. a mean effect size of -0.2 or lower, which was not statistically significant). In contrast, the study from Pak and colleagues, for which no SMD could be derived, reported a median difference of -0.6 [-0.9 to -0.4] (0-100 Scale, positive values favor telerehabilitation) [53].

Five studies compared telerehabilitation to a brochure-based home exercise program [45, 46, 52, 57, 58]. Herein, SMDs ranged between − 0.59 and 0.02. The study from Meijer and colleagues [52] only reported visual results, indicating no between-group difference for average pain on a NRS scale. Two other studies yielded no difference between groups [46, 58]. One study found a tendency of benefit for telerehabilitation [45] and another yielded a statistically significant effect in favor of telerehabilitation [57].

Four studies with a tele-intervention as add-on to standard care reported on pain [12, 61–63], with SMDs ranging from − 0.81 to -0.08. The effects sizes pointed towards tendency in favor of telerehabilitation [62], statistically significant effect in favor of telerehabinlitation or no between group difference [12, 61, 63].

**Fig. 3:**
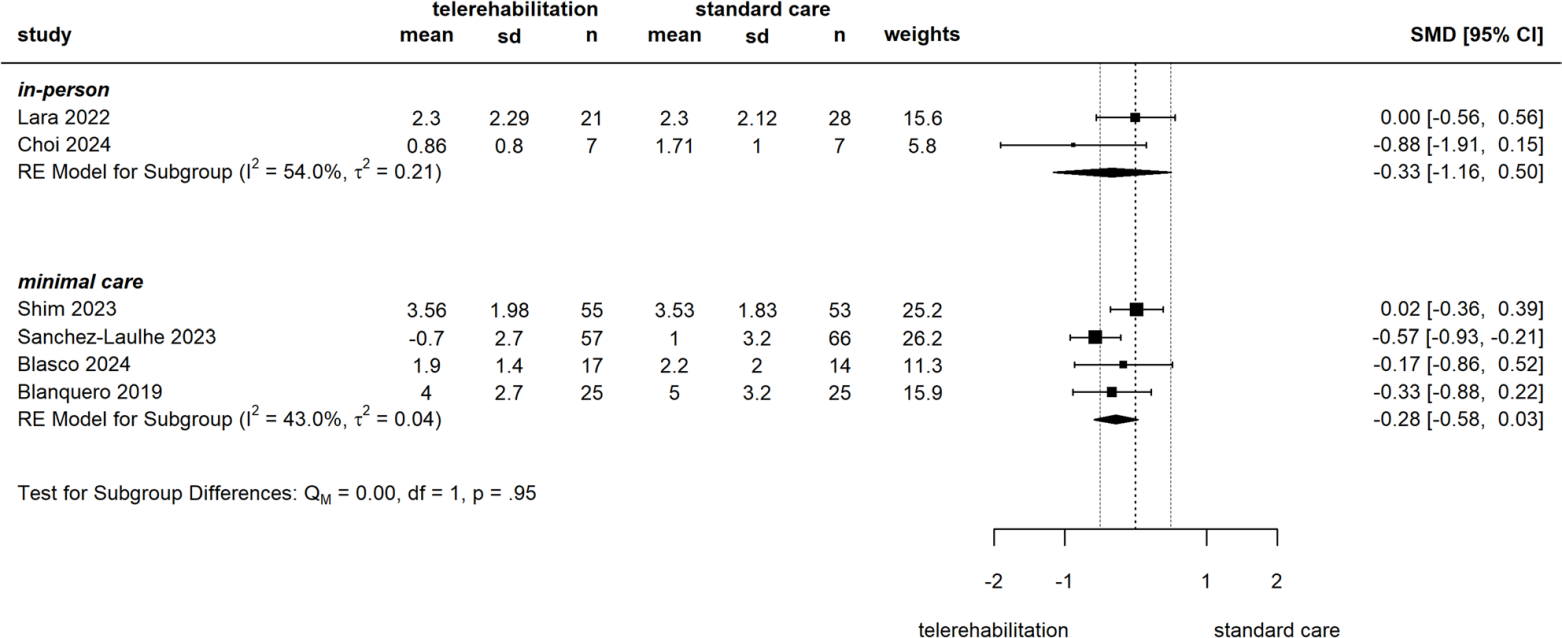
Meta-analysis of pain (NRS/VAS), telerehabilitation versus standard care SMD: Standardized mean difference, CI: confidence interval, sd: standard deviation, RE: random effects

**Fig. 4:**
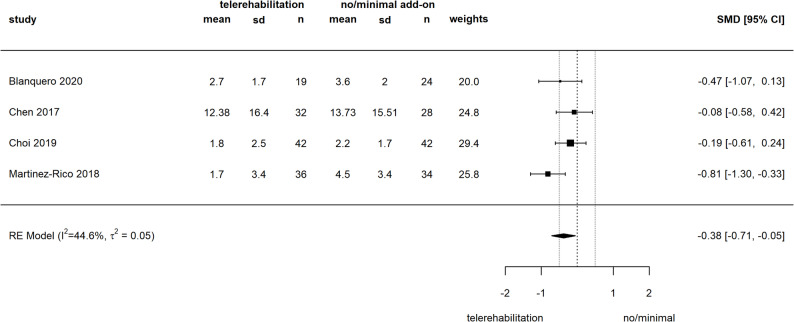
Meta-analysis of pain (NRS/VAS), telerehabilitation as adjunct versus no/minimal adjunct SMD: Standardized mean difference, CI: confidence interval, sd: standard deviation, RE: random effects

From 15 studies reporting on pain, 13 could be included into meta-analyses. In comparison of telerehabilitation versus in-person care, our meta-analysis of pain measured by VAS/NRS (two studies could be included) yielded an SMD of -0.33 [-1.16 to 0.5] (see Fig. 3), with one study at some concerns [50] and another at high [47] risk of bias. Pooled Constant-Murley scores (CMS) showed an SMD of -0.38 [-0.93 to 0.18] (see Fig. 9 in Additional file 6), derived from two studies with some concerns for risk of bias [54, 59]. Compared to a minimal form of standard care, four studies were pooled to an SMD of -0.28 [-0.6 to 0.03] in VAS/NRS (see Fig. 3) with two studies at some concerns [45, 46] and another two at high [57, 58] risk of bias. In studies, which implemented telerehabilitation as an adjunct to standard care, meta-analysis of VAS/NRS pain scores yielded an SMD of -0.38 [-0.71 to -0.05] (see Fig. 4). The risk of bias was judged high in all four contributing studies [12, 61–63]. Subgroup analysis in telerehabilitation versus minimal care showed higher effects towards telerehabilitation in hand and wrist disorders and for intervention duration ≥ 12 weeks, but no influence of overall risk of bias. For telerehabilitation as adjunct sensitivity analysis showed robustness against influence of one study with several risk of bias concerns [12] subgroup analysis revealed higher effect for shorter intervention duration and no difference between hand and shoulder region (for subgroup analysis see Additional file 6, Fig. 2–8).

#### ADL

Nineteen trials reported on the outcome ADL [12, 44–53, 55–61]. When trials used in-person rehabilitation as a comparison [44, 47–51, 53, 55, 56, 59, 60], the effects were highly variable, with average SMDs ranging from − 0.72 to 0.59. The results from primary studies either indicate no between group difference [44, 48, 49, 51, 53, 56, 59, 60], a tendency in favor of telerehabilitation [47, 50, 59], or a tendency in favor of in-person care [51, 55].

In five studies comparing telerehabilitation to a minimal form of standard care [45, 46, 52, 57, 58], SMDs ranged from − 1.33 to 0.16. Herein, results from three studies tended to favor telerehabilitation [46, 57, 58], with statistically significant findings in three studies [41–43, 53, 54]. In two studies, results showed no between group difference [52, 58].

Three studies with a tele-intervention as add-on to standard care examined ADL with SMDs ranging from − 0.57 to 0.04 [12, 61, 62]. In two studies, the results suggested a potential superiority of the tele-intervention [61, 62] over standard care alone. In contrast, a third study found no difference between the groups [12].

**Fig. 5:**
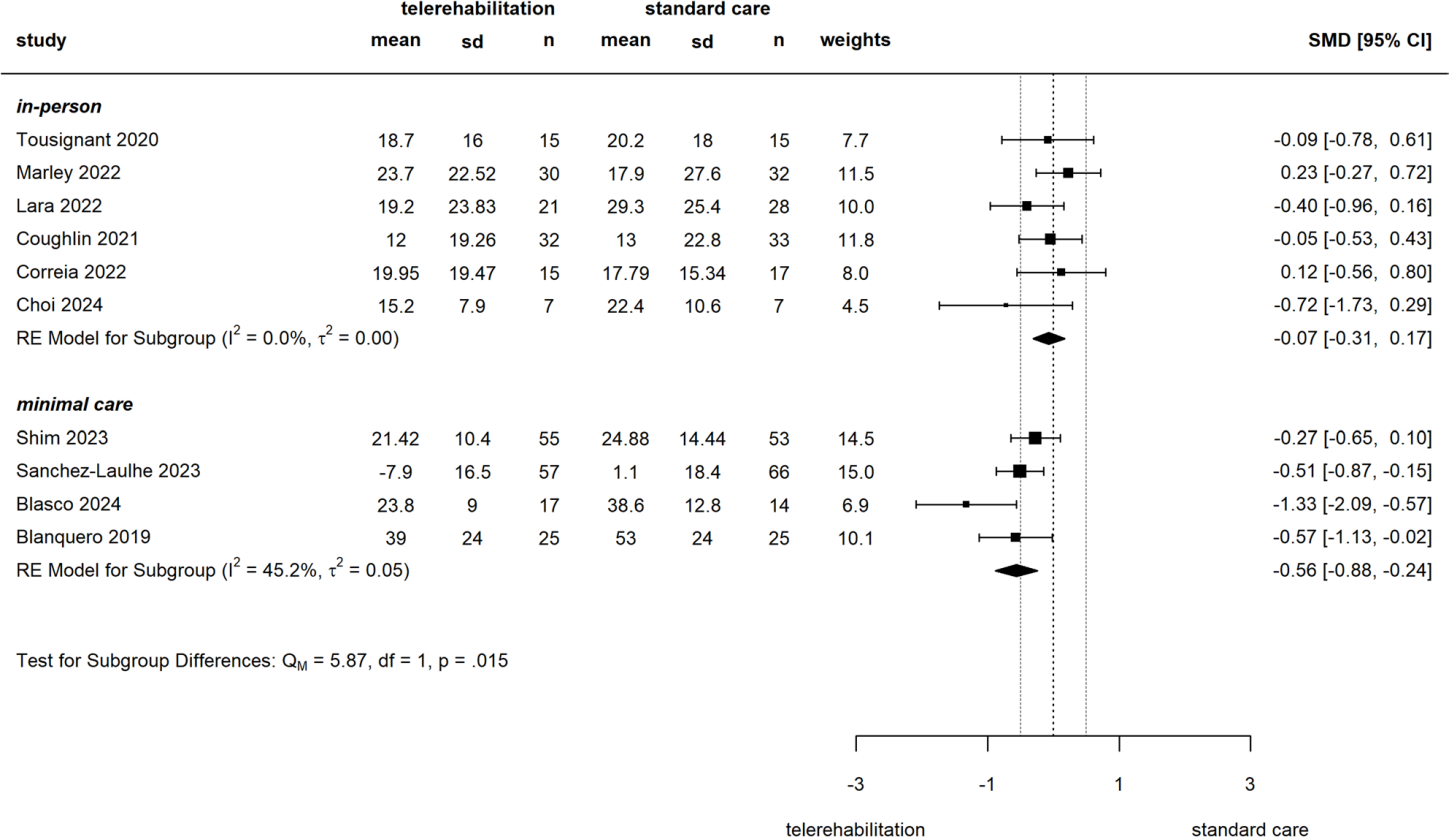
Meta-analysis of ADL (QuickDASH/DASH), telerehabilitation versus standard care SMD: Standardized mean difference, CI: confidence interval, sd: standard deviation, RE: random effects

**Fig. 6:**
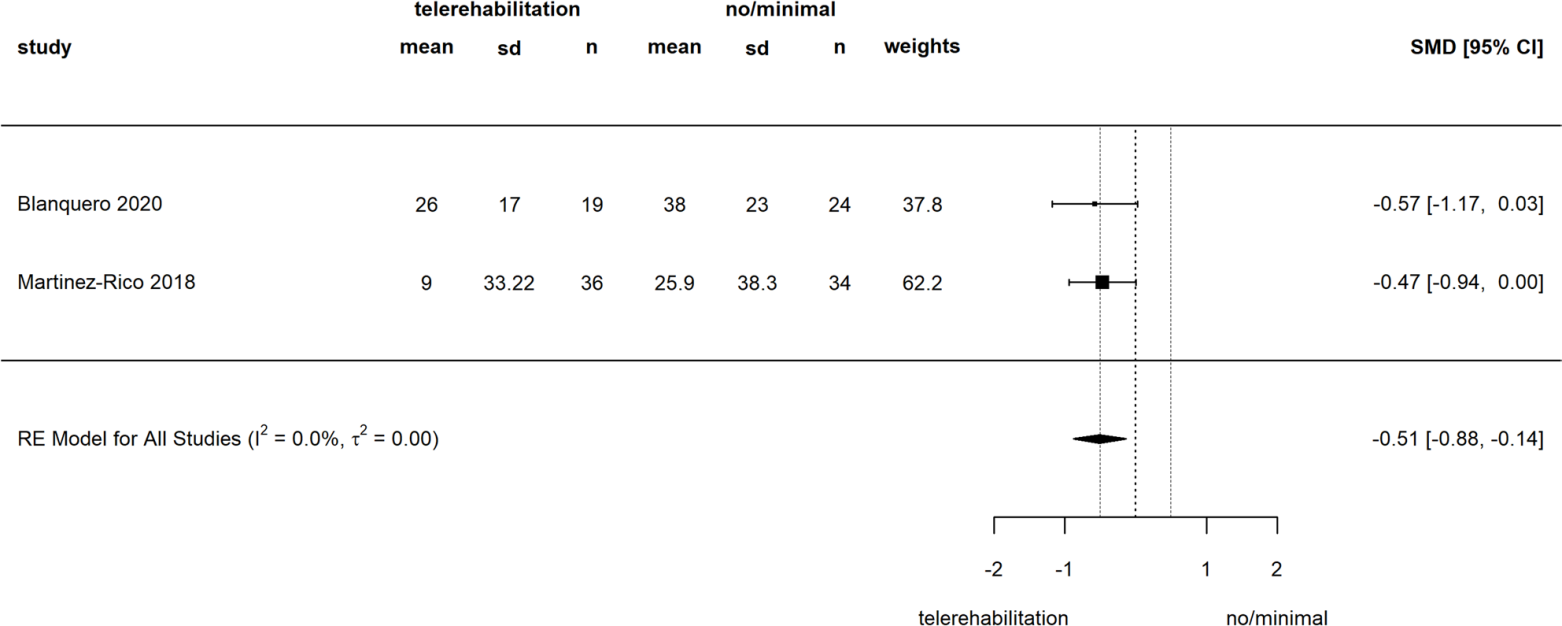
Meta-analysis of ADL (QuickDASH/DASH), telerehabilitation as adjunct versus no/minimal adjunct SMD: Standardized mean difference, CI: confidence interval, sd: standard deviation, RE: random effects

From 19 studies with report of ADL we were able to include 13 into meta-analyses. Comparing telerehabilitation to in-person care, our meta-analysis of the DASH and its short form yielded an SMD of -0.07 [-0.31 to 0.17] (see Fig. 5). All contributing studies were judged to have some concerns in risk of bias [48–51, 59], except for one study with high bias risk in several domains [47]. For CMS, pooled SMD was calculated with 0.11 [-0.32 to 0.54] (see Fig. 19 in Additional file 6), with all three studies with some concerns for risk of bias [48, 55, 59]. Compared to a minimal form of standard care, we derived a pooled SMD for QuickDASH/DASH of -0.56 [-0.88 to -0.24] (see Fig. 5). Two contributing studies had some concerns for risk of bias [45, 46] and another two were at high risk of bias [57, 58]. Two studies examining a tele-intervention as add-on [61, 62] were pooled for QuickDASH/DASH to an SMD of -0.51 [-0.88 to -0.14] (see Fig. 6). The risk of bias was judged high for both studies [61, 62]. Within subgroup analysis for telerehabilitation versus in-person care in QuickDASH/DASH effect favored telerehabilitation more in one study with high risk of bias [47] and in hand/wrist disorders, but no difference was found for intervention duration. In the subgroup analysis for telerehabilitation versus minimal care in QuickDASH/DASH, effects were more in favor of telerehabilitation for studies with some concerns of risk of bias. Results proved stable against exclusion of one small study [46] and for intervention duration and body region influence remained unclear (for subgroup analysis see Additional file 6, Figs. 11, 12, 13, 14, 15, 16, 17, 18, 19 and 20).

#### HrQol

**Fig. 7:**
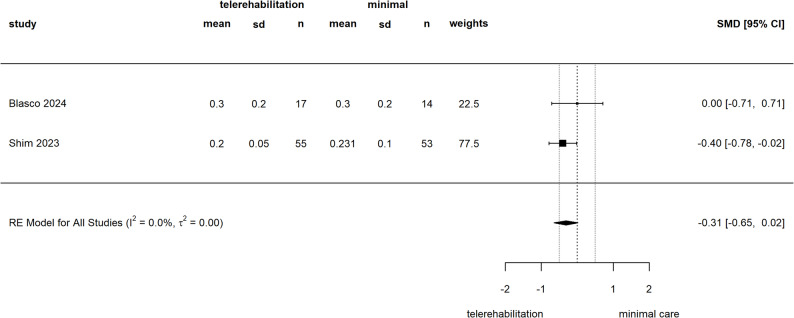
Meta-analysis of HrQol (EQ-5D-5 L), telerehabilitation versus standard care SMD: Standardized mean difference, CI: confidence interval, sd: standard deviation, RE: random effects

Four studies reported on HrQol [46, 50, 51, 58]. In two studies comparing telerehabilitation against in-person rehabilitation, one found no statistically significant difference between the groups on the EQ-VAS Scale [51], while another found an effect that tended to favor telerehabilitation on the Veterans RAND 12 (VR-12) physical scale [50]. In two studies comparing telerehabilitation to a brochure-based home exercise program, results also differed [46, 58]. One study found no between group difference on EQ-5D-5 L Score [46], while the second study found a significant effect in favor of telerehabilitation on EQ-5D-5 L Score [58]. Only two [46, 58] out of four studies could be pooled. For comparison of telerehabilitation against brochure-based home exercise, we found an SMD of -0.31 [-0.65 to 0.02] (see Fig. 7), with one high bias risk study [58] and one with some concerns for risk of bias [46].

### GRADE

GRADE ratings are presented in Table 2 (summary of findings). Since all NRSI studies were excluded from synthesis ultimately, the GRADE ratings are based on RCTs only. We downgraded all results one level for risk of bias because all studies were judged with at least some concern in at least one RoB 2 domain. In one case, rating was downgraded twice within the risk of bias domain, because of substantial concerns. As noted above common issues regarding risk of bias were related to unblinded outcome report. Additionally, missing data and insufficient outcome predefinition contributed to a reduced certainty of evidence. Industrial funding and conflicts of interest were not included within RoB 2 but were taken into account within GRADE domain publication bias. Herein, three results were downgraded primarily due to the influence of small studies and conflict of interest issues. Five results were downgraded for imprecision due to confidence intervals crossing the above defined margin and in one case, sample size was issued, because the calculated optimal information size was not reached. For pain, certainty of evidence was judged to be very low to low. For ADL, moderate certainty of evidence was found except for CMS (very low). Further, certainty of evidence was judged low for HrQol.

**Table 2:** Summary of findings

Instrument comparison	Effect with telerehabilitation SMD [95% CI] direction standardized: negative values favor telerehabilitation	*N* participants (studies)	GRADE rating (and interpretation)
	MD [95% CI] transformed MDs are presented along with instrument details^ʠ^		
Outcome domain: Pain			
VAS/NRS telerehabilitation versus in-person care	SMD: Mean pain score in SD units was 0.33 lower [1.16 lower to 0.5 higher]	63 (2)	**Very low** ^a, a, b^ We are uncertain whether telerehabilitation improves VAS/NRS pain scores to a similar extent compared to in-person care.
	MD: Mean pain score in VAS (0–10, lower is better) was 0.51 lower [1.80 lower to 0.78 higher]^ʄ^		
CMS pain scale telerehabilitation versus in-person care	SMD: Mean pain score in SD units was 0.38 lower [0.93 lower to 0.18 higher]	48 (2)	**Very low** ^a, c, d^ We are uncertain whether telerehabilitation improves CMS pain scores to a similar extent compared to in-person care.
	MD: Mean pain scores in CMS pain scale (0–12, higher is better) was 0.85 higher [0.4 lower to 2.11 higher]^ʈ^		
VAS/NRS telerehabilitation versus minimal care	SMD: Mean pain score in SD units was 0.28 lower [0.58 lower to 0.03 higher]	312 (4)	**Low** ^a, b^ telerehabilitation may decrease pain VAS/NRS slightly when compared to minimal standard care
	MD: Mean pain scores NRS (0–10, lower is better) was 0.49 lower [1.04 lower to 0.06 higher]^ʅ^		
VAS/NRS telerehabilitation add-on versus no/minimal add-on	SMD: Mean pain score in SD units was 0.38 lower [0.71 lower to 0.05 lower]	257 (4)	**Low** ^a, d^ telerehabilitation as add-on to standard care may decrease pain VAS/NRS slightly when compared to no/minimal add-on
	MD: Mean pain scores VAS (0–10, lower is better) was 0.75 lower [1.41 lower to 0.09 higher] ^ʋ^		
Outcome domain: ADL			
QuickDASH/DASH telerehabilitation versus in-person care	SMD: Mean functional score in SD units was 0.07 lower [0.32 lower to 0.17 higher]	252 (6)	**Moderate** ^a^ We are uncertain whether telerehabilitation improves QuickDASH/DASH scores to a similar extent compared to in-person care.
	MD: Mean functional score in QuickDASH (0-100, lower is better) was 0.91 lower [4.15 lower to 2.32 higher] ^ʏ^		
CMS telerehabilitation versus in-person care	SMD: Mean functional score in SD units was 0.11 higher [0.32 lower to 0.54 higher]	80 (3)	**Very low** ^a, b,d^ We are uncertain whether telerehabilitation improves CMS scores to a similar extent compared to in-person care.
	MD: Mean functional scores in CMS (0-100, higher is better) was 1.47 lower [7.08 lower to 4.14 higher]^ʮ^		
QuickDASH/DASH telerehabilitation versus minimal care	SMD: Mean functional score in SD units was 0.56 lower [0.88 lower to 0.24 lower]	312 (4)	**Moderate** ^a^ telerehabilitation likely improves QuickDASH/DASH scores when compared to minimal standard care
	MD: Mean functional scores in units of QuickDASH (0-100, lower is better) was 9.71 lower [15.29 lower to 4.12 lower] ^ʡ^		
QuickDASH/DASH telerehabilitation add-on versus no/minimal add-on	SMD: Mean functional score in SD units was 0.51 lower [0.88 lower to 0.14 lower]	113 (2)	**Moderate** ^a^ telerehabilitation as add-on to standard care likely decreases QuickDASH/DASH scores when compared to no/minimal add-on
	MD: Mean functional score in QuickDASH (0-100, lower is better) was 10.32 lower [17.85 lower to 2.78 lower] ^¥^		
Outcome domain: HrQol			
VR-12 physical scale telerehabilitation versus in-person care	SMD: Mean score in SD units was 0.26 lower [0.82 lower to 0.30 higher]	51 (1)	**Low** ^a, b^ We are uncertain whether telerehabilitation improves VR-12 scores to a similar extent compared to in-person care.
	MD: Mean HrQol score in VR-12 (0-100, higher is better) was 3.05 higher [3.58 lower to 9.68 higher]^¢^		
EQ-5D-5 L telerehabilitation versus minimal care	SMD: Mean score in SD units was 0.31 lower [0.65 lower to 0.02 higher]	139 (2)	**Low** ^a, b^ telerehabilitation may improve HrQol slightly when compared to minimal standard care
	MD: Mean HrQol score in EQ-5D-5 L (0–1, higher is better) was 0.04 higher [0.00 lower to 0.09 higher] ^£^		
HrQol telerehabilitation add-on versus no/minimal add-on	Outcome was not measured	NA	NA

*SMD* Standardized mean difference, *SD* standard deviation, *MD* mean difference, *CI* confidence interval, *NA* not applicable, *VAS* visual analogue scale, *NRS* numeric rating scale, *CMS* Constant-Murley score, *ADL* activities of daily living, *DASH* Disabilities of the Arm, Shoulder and Hand questionnaire, *HrQol* health-related quality of life, *VR-12* Veterans RAND 12-Item Health Survey, *EQ-5D-5 L* EuroQol five dimensions five levels measurement

^ʠ^transformation of SMD to MD is based on following estimation of SD, which are derived from studies within the same meta-analysis measuring in the MD unit to which transformation is applied: ^ʄ^1.55 ^ʈ^2.27 ^ʅ^1.79 ^ʋ^1.98 ^ʏ^13.26 ^ʡ^17.36 ^ʮ^13.1^¥^20.35 ^¢^11.86 ^£^0.14

^a^downgrade one level for risk of bias

^b^downgrade one level for imprecision due to wide confidence intervals

^c^downgrade one level for imprecision due to small sample size

^d^downgrade one level for publication bias, if effect was influenced by small studies with at least one having conflicts of interest issues

## Discussion

We conducted a systematic review with meta-analysis to assess the effectiveness of telerehabilitation in different implementation modes on the outcomes pain, activities of daily living and health-related quality of life. Final synthesis comprised 20 RCTs. Pooled effects tended to support similar improvements with telerehabilitation compared to in-person rehabilitation but certainty of evidence ranged from very low to moderate. Telerehabilitation improved outcomes compared to minimal care or when used as adjunct to standard care with low to moderate certainty of evidence.

### Pain

For pain, the results from primary studies and pooled analysis suggested that telerehabilitation leads to similar improvements compared to in-person rehabilitation. Due to small number of studies and influence of risk of bias, these results were rated to be of very low certainty of evidence using GRADE. In addition, the study from Pak and colleagues [53], which only had small concerns for risk of bias, but of which data could not be integrated into meta-analysis, reported a small effect favoring in-person rehabilitation for average pain. Compared to a minimal form of standard care, we judged certainty of evidence low that telerehabilitation reduces pain. Herein, we downgraded one level for imprecision, because the confidence interval slightly overlapped the zero margin. In general, it could be argued that the zero margin is not sufficiently conservative, because no downgrade is applied if a negligible effect size in favor of teleinterventions falls within in the confidence interval; a margin of -0.2 would therefore have been more conservative. Hence, there is some doubt remaining that the effect size we found is relevant. In their systematic review, Molina-Garcia and colleagues reported an SMD of -0.19 [-0.49 to 0.11] for hand and wrist disorders, which is comparable to our results [64]. In shoulder disorders, Huang and colleagues [65] compared telerehabilitation to home based exercise in their systematic review. For pain, they found an SMD of -0.19 [-0.60 to 0.23], which is close to our result, although reviews substantially differ in terms of inclusion criteria (included diagnoses) and grouping (standard care could be either in-person or minimal care). The authors subgrouped for length of intervention, which dissolved high statistical heterogeneity, and at the same time resulted in a statistically significant effect for > 12 weeks of intervention duration. This pattern could actually be reproduced within our review yielding a null effect for studies with duration shorter than 12 weeks [45, 58] and a statistically significant effect in favor of telerehabilitation for intervention duration of 12 weeks or longer [46, 57]. In other outcomes though, different patterns were observed.

For telerehabilitation as add-on to standard care, we found low certainty of evidence in pain reduction. Again, our margin of zero for GRADE domain imprecision is debatable. Within this comparison, the study with most concerns of risk of bias favored the control group, which underlines the robustness of our findings against risk of bias. The study from Martinez-Rico and colleagues [61] stood out because it was the only study with a large effect (SMD: -0.81 [-1.30 to -0.33]). Two reasons may explain this: First, this study used a synchronous mode of telerehabilitation, with patient and carer communicating over telephone. This personal interaction may be more motivating than rehabilitation support over a technical device. Second, the study population was substantially younger (mean age: 28 years) than in the other studies, which may have led to this result. Since underlying mechanism are unexplained these hypotheses remain speculative. In previous reviews, separate analysis of adjunct telerehabilitation is rare, which makes direct comparison with these results difficult. Gava and colleagues [66] found low certainty of evidence that there is no difference between telerehabilitation and home exercising in shoulder disorders, on basis of the study from Choi and colleagues [63]. We could include further three studies into the analysis, also due to our broader scope of diagnoses, which led to a statistically significant finding. Only including shoulder disorders decreased effect size to a small degree.

### ADL

For the outcome ADL we found very low to moderate certainty of evidence that telerehabilitation leads to similar improvements compared to in-person care. Of note, the most problematic study in regard to risk of bias [47] yielded the highest effect in favor of telerehabilitation for QuickDASH/DASH. Sensitivity analysis without this study only led to small changes of upper CI level, therefore, no further downgrade was undertaken. On the other hand, the study from Pak and colleagues [53], not integrated within the meta-analysis, found tendency for better results with in-person care, questioning robustness of the meta-analysis. For CMS, certainty of evidence was very low due to downgrades in several domains. Gava and colleagues [66] reported very low certainty of evidence for no difference between groups in disability, including Roddey et al. and Marley et al. into their analysis [51, 56]. In our analysis, more studies were included, mainly because of a broader diagnosis scope. This led to a narrower confidence interval for ADL and subsequently to no downgrade for imprecision. Within separate analysis for diagnosis, effect size tended more towards in-person care in shoulder disorders, leading to imprecise CIs in shoulder disorders. Comparing telerehabilitation to minimal care, we found moderate certainty of evidence for improved ADL. Herein, the study with least risk of bias issues [45] contributed a moderate and statistically significant effect, strengthening certainty of the evidence. Our result, which represents a moderate effect size, was a little bit higher than findings from Huang and colleagues for DASH (MD:-4.51 [-8.70 to -0.32], on a 0-100 scale) [65]. This may be explained by our separate grouping for in-person and minimal care, although direct comparison of reviews is difficult due to differences in diagnosis scope. In hand and wrist disorders, Molina-Garcia and colleagues [64] found an SMD of 0.33 [0.14 to 0.52] (positive values favor telerehabilitation), but based only on one study, and for disability, three studies yielded an SMD of 0.57 [0.16 to 0.98] (positive values favor telerehabilitation). Especially the latter result is very similar to our finding of QuickDASH/DASH, which were stable when analyzing only hand disorders. Moderate certainty of evidence was found in ADL for tele-intervention in addition to standard care derived from only two studies. Compared to the outcome pain, effect size was larger, reaching a moderate size.

### HrQol

HrQol was rarely investigated within included studies. We found low evidence for similar improvements with telerehabilitation compared to in-person care, but this evidence is based on a sole study, limiting the generalizability of the findings. Compared to minimal care, we judged evidence low for improved HrQol. The result was downgraded for imprecision because the 95% CI overlapped the zero margin. Huang and colleagues reported a MD of 0.04 [0.01 to 0.07] (on 0–1 scale, positive values favor telerehabilitation), which is in tendency comparable to our finding of a small effect size. No study implementing a tele add-on reported on this outcome.

### Telerehabilitation compared to in-person rehabilitation

The results from primary studies and pooled analysis suggested that there was no difference between telerehabilitation and in-person rehabilitation. Applying the GRADE approach, the certainty of evidence was limited for similar improvements with telerehabilitation compared to in-person rehabilitation: Ratings ranged from very low (pain, ADL by CMS) to low (HrQol), and moderate (ADL by QuickDASH/DASH). The reasons for downgrading were mostly risk of bias concerns and imprecision due to CIs crossing the 0.2 margin, but concerns for publication bias were also issued. Further, one trial with a fully remote telerehabilitation protocol was stopped halfway because nearly all participants crossed over to in-person rehabilitation [60]. The data from this study could not be included into meta-analysis, but authors conclude that patients’ preference may be directed towards in-person rehabilitation. They explain this with patients missing the hands-on component, the personnel contact and direct exercise correction. The authors suggest that telerehabilitation protocols need to be further improved. To this end, they propose blended interventions (e.g. mix of telerehabilitation and in-person sessions) as an alternative telerehabilitation mode. In our review we could only include one study using a blended interventions [48]. Since stakeholders in rehabilitation have repeatedly requested that telerehabilitation may be combined with in-person sessions [67–69] this mode may be particularly worthwhile to study in future trials.

In comparison to in-person rehabilitation, telerehabilitation is used as an alternative delivery mode. This suggests that studies should investigate whether telerehabilitation is non-inferior to in-person care. Within this review only a subset of the included trials made use of such an approach. Others indirectly implied equivalence (e.g., based on absence of statistically significant results), but without specifying non-inferiority hypotheses or comparing results to predefined margins. Further, investigation of non-inferiority requires a specific risk of bias assessment [70]. This includes the assessment of control interventions, for which effectiveness should be established. Because we did not undertake such an assessment and because primary studies used mixed approaches, we cannot provide robust conclusions on non-inferiority. Trials that compare telerehabilitation to in-person rehabilitation applying sound non-inferiority methodology therefore are needed.

Other reviews have investigated telerehabilitation compared to in-person rehabilitation. The guideline of the American Physical Therapy Association found critical outcomes from included studies to be similar between telerehabilitation and in-person rehabilitation, examining a broad diagnostic scope. Recommendation strength was upgraded from low to moderate due to consistency of findings and the practical inability of blinding within the interventions. The latter problem was also an object of debate within our risk of bias assessment. However, following RoB 2 instructions, the judgement of at least some concerns in risk of bias was upheld, and in four cases, it influenced the overall RoB 2 judgement. In most cases though, bias risks were raised in more than one domain, confirming concerns. In shoulder disorders, the review from Gava and colleagues [66] stated very low certainty of evidence for no difference in pain intensity and disability. This is in line with our findings for pain, but differing in the outcome ADL (as outlined above). A recent Cochrane review on neck pain [71] stated very low certainty of evidence for little/no difference between telerehabilitation versus matched control intervention for pain and function. Although some outcomes within our review were rated with moderate certainty of evidence others received a very low certainty of evidence rating. Therefore we have to repeat preceding calls for primary studies in this field, adequately sampled and using a matched control intervention reflecting evidence-based care [11]. Findings from this review should be taken into account and be weighed against considerations of cost and adverse events. In respect to cost, evidence from a recent systematic review [20] demonstrated cost-effectiveness for telerehabilitation versus in-person rehabilitation under specific conditions: probability for cost-effectiveness was 90% at a willingness-to-pay threshold of $30,000 per quality adjusted life year (QALY). In regard to adverse events, the outcome is rarely investigated within systematic reviews of telerehabilitation [11]. However, a recent review has found telerehabilitation to be safe [72].

Because of remaining uncertainty in the evidence, telerehabilitation should best be considered in situations, where an in-person rehabilitation is restricted, whether because of individual (e.g. long travel distance, patients’ preference) or other reasons (e.g. pandemic situation, restricted resources). Some of these barriers to rehabilitation may be particularly pronounced in the working population. For instance, employed individuals may be less likely to complete a rehabilitation program [73]. Furthermore, stakeholders in rehabilitation of occupational injury of the upper limb have stated that time savings and the flexible integration of telerehabilitation are relevant beneficial factors [68].

### Telerehabilitation compared to a minimal form of standard care

In our review, telerehabilitation improved outcomes compared to brochure-based exercising with low (pain, HrQol) to moderate (ADL) certainty of evidence. As stated above, effect sizes remained low. This is particularly relevant because many advantages related to telerehabilitation like cost savings, saving of personal resources, and savings of carbon emissions are not applicable in these situations. Therefore, the rather slight beneficial effects have to be weighed against its costs in this specific implementation setting under consideration of possible adverse effects. However, evidence for telerehabilitation’s cost-effectiveness is primarily based on the comparison to in-person care [20], limiting transferability to the comparison to minimal care. Of note, the trials within this review focused on exercise interventions and rarely used comprehensive rehabilitation approaches, which could have limited effectiveness of the telerehabilitation intervention. However, a systematic review on work participation interventions for upper limb MSDs found a positive association between exercise interventions and work-related outcomes, whereas evidence was insufficient for multidisciplinary interventions [74].

#### Telerehabilitation as adjunct to standard care compared to no/minimal adjunct

Lastly, this review found low (pain) to moderate (ADL) certainty of evidence for the benefit of telerehabilitation as adjunct to standard care. Within this comparison the study of Martinez-Rico et al. [61] found a statistically significant beneficial effect of a telerehabilitation coaching-intervention via telephone. Additionally, the study of Blanquero and colleagues, using an app-based and therapist-monitored intervention, yielded considerable improvements [62]. In contrast, the mere usage of reminder messages did not lead to group differences [12]. This may suggest that more intensive tele-interventions with implemented therapist-feedback are promising, whereas minimal, technology-driven interventions may not be worthwhile. Only four studies could be included within this comparison. Hence, future studies investigating the benefit of a telerehabilitation-adjunct are needed and should include report on HrQol, for which evidence was lacking. Within this comparison, the same considerations apply as for telerehabilitation versus minimal care: The benefit has to outweigh additional costs and possible harms. A study investigating the benefit of an additional telerehabilitation aftercare program after total hip or knee replacement found no difference in key functional outcomes [75]. However, return-to-work rates were significantly higher in the intervention group. The added value of teleinterventions in the occupational context may therefore be more evident in the time needed to resume work.

### Health equity

Studies within this review often excluded patients with language or technological barriers and such criteria are coherent considering feasibility. However, given the promise of increased care-access associated with telerehabilitation [17, 18], future studies should consider participation of the different groups within a society, thereby ensuring that these interventions decrease health-inequality, instead of aggravating it further. This could be achieved through participative intervention development and the delivery of telerehabilitation in different languages [23, 76, 77]. From a global perspective it has to be pointed out that research on telerehabilitation remains highly desirable in low- and middle-income countries, where there is a pronounced lack of rehabilitation service coverage [78]. As we set our focus on high-income countries only, the external validity of our results is limited and results may not be transferred to low or middle-income economies.

### Strengths & limitations

The strengths of this review lie in our rigorous literature search and sound methodology. Furthermore, distinguishing between different application models of telerehabilitation is a strength of this review, as it shows that these models are associated with varying degrees of effectiveness. However, some limitations should be considered in the interpretation of the findings. Tele-interventions as well as control interventions comprised a variety of modalities in terms of technologies used, rehabilitative content, intervention length, and dosage. We made efforts to cluster the analyses in a meaningful way and to provide subgroup analysis for relevant variables. However, we were not able to address all possible factors that may have introduced heterogeneity. Our restriction to publications in English and German may introduce publication bias [79]. Nevertheless, we consider this influence to be small, because no language filters were applied within the search strings and databases almost completely provide titles and abstracts in English. One citation was excluded during full-text screening due to its language, but this study also did not meet the high-income country criteria. When transferring the results to a working population, the high mean age of included patients (56 years) should be reflected, because pronounced barriers in telerehabilitation usage may be expected in older ages [23], which may have lowered effect estimates. Furthermore, meta-analysis was compromised due to the limited number of studies within each comparison. This hindered subgroup analysis to inform the influence of disease type, age, gender or intervention details on the results. Lastly, our standardization of effect direction (i.e., negative values for SMDs favor telerehabilitation, regardless of underlying outcome or instrument) is uncommon, but was carried out to facilitate readability. This may be misleading specifically for instruments (e.g. within HrQol), which are predominantly directed towards positive values indicating benefit. However, the transformation is transparently reported and repeatedly indicated.

### Future directions & clinical implications

The limited certainty of evidence across all comparisons and outcomes underscores the need for further high-quality research in this field. This is particularly evident for comparisons with in-person rehabilitation, and for the outcome pain, where certainty of evidence included very low ratings. Future studies comparing telerehabilitation to in-person rehabilitation should employ non-inferiority designs using adequate methodology. Furthermore, very few studies investigated health-related quality of life or the usage of teleinterventions as adjunct to standard care.

The studies included in this review predominantly demonstrated comparable improvements for telerehabilitation relative to in-person rehabilitation, as well as modest improvements when rehabilitation was augmented with teleinterventions. Therefore, from an effectiveness point of view, broader implementation appears justifiable, particularly given that the safety of telerehabilitation so far is considered to be acceptable [72]. Nevertheless, given the persistently limited certainty of evidence for effectiveness identified in this review, and the unresolved question of equal uptake across different social groups [22, 77, 80, 81], teleinterventions should preferably be proposed as an optional mode, based on the preference of the patient. The German Pension Insurance, for instance, currently provides rehabilitation aftercare programs in both outpatient and telemedicine formats [82]. Furthermore, previous studies and findings from one trial within this review indicate that teleinterventions should be therapist-guided and that a minimum number of in-person meetings may be crucial for a successful adaptation [60, 67–69]. A distinct and ongoing challenge remains the development of inclusive teleinterventions, which could contribute to improving overall access to rehabilitation services [23].

## Conclusion

The field of telerehabilitation has been the subject of extensive research in recent years. The complexity of telerehabilitation interventions allows for various modalities, including different content, modes, and levels of technology involvement. Further, it can be investigated within a wide spectrum of diseases and in different comparison modalities. This variability hampers comparisons between different studies and has produced a spectrum of systematic reviews. However, these reviews have predominantly suggested that telerehabilitation is superior or equal to standard care, although methodological quality of the evidence is overall limited. This review contributes to the existing body of evidence in the rehabilitation of upper limb musculoskeletal disorders by providing findings on the effectiveness of telerehabilitation in various implementation settings. These include the comparison to in-person rehabilitation, replacing of brochure-based home exercising, and usage of a tele-adjunct to standard care. For comparison to in-person rehabilitation, effects tended to show similar improvements with telerehabilitation but certainty of evidence is limited with very low to moderate ratings. For comparison to brochure-based exercising and for the usage of tele-adjunct, our findings suggest slight improvements with telerehabilitation (low to moderate certainty of evidence). Future trials are needed investigating non-inferiority of telerehabilitation against in-person rehabilitation and the value of telerehabilitation protocols as adjunct to standard care. Furthermore, HrQol outcomes should be included within primary studies. In the implementation of telerehabilitation, findings from this review should be thoroughly balanced against considerations of harms, costs and patients’ preference within the specific rehabilitation context.

## Supplementary Information

Additional file 1: PRISMA 2020 Checklist.

Additional file 2: Database search strategy: search strings for electronic databases MEDLINE, Embase and AMED.

Additional file 3: List of exclusions during full text screen: List of excluded studies within full-text screening along with reason for exclusion and reference.

Additional file 4: Risk of Bias Assessment: RoB 2 assessment of individual results on domain level, ROBINS-I assessments for included NRSIs.

Additional file 5: Main characteristics and results from included RCTs: Characteristics and results from RCTs for time point directly after intervention.

Additional file 6: Results from meta-analysis: Forest plots of meta-analysis results including subgroup analysis.

Additional file 7: Results from studies reporting on follow up later than end of intervention.

Additional file 8: Characteristics and results of Non-Randomized Studies of Interventions (NRSIs).

## Data Availability

The datasets used and analyzed during the current study are available from the corresponding author on reasonable request.

## Abbreviations

ADL: Activities of daily living
AMED: Allied and Complementary Medicine Database
CMS: Constant-Murley score
DASH: Disabilities of the Arm, Shoulder and Hand questionnaire
Embase: Excerpta Medica database
EQ-5D-5L: EuroQol five dimensions five levels measurement
EQ-VAS: EuroQol visual analog scale
HrQol: Health-related quality of life
IQR: Interquartile range
MEDLINE: Medical Literature Analysis and Retrieval System Online
MD: Mean difference
MSDs: Musculoskeletal disorders
NRS: Numeric rating scale
NRSI: Non randomized studies of interventions
OSIS: Oxford Shoulder Instability Score
PICOS: Population, Intervention, Comparison, Outcome, Study design
QuickDASH: Quick Disabilities of the Arm, Shoulder and Hand questionnaire
QALY: Quality adjusted life years
RCT: Randomized controlled trials
RoB 2: Revised Cochrane risk-of-bias tool for randomized trials
ROBINS-I V2: The Risk of Bias in Non-randomized Studies – of Interventions, Version 2
SD: Standard deviation
SMD: Standardized mean difference
VAS: Visual analogue scale
