# Supplementary material for: The effectiveness of telerehabilitation in upper limb musculoskeletal disorders: a systematic review
Source: BMC Musculoskelet Disord. 2026 May 28;27:462. doi: 10.1186/s12891-026-10008-7 (PMC13220470; doi:10.1186/s12891-026-10008-7)
Supplement: Supplementary file 2 — Additional file 2: Database search strategy: search strings for electronic databases MEDLINE, Embase and AMED. [file 12891_2026_10008_MOESM2_ESM.docx]

Database search strategy

27.11.2024

**References for Medline (via Ovid) string:**

For concept “controlled trial” the search string was adopted with small adjustments from:

Cochrane Effective Practice and Organization of Care (EPOC). How to develop a search strategy for an intervention review. EPOC Resources for review authors, 2017. https://zenodo.org/records/5106292 (accessed 07.11.2024), p.7.

For concept “telerehabilitation” the search string was partly adopted from:

Cox, N. S., Dal Corso, S., Hansen, H., McDonald, C. F., Hill, C. J., Zanaboni, P., Alison, J. A., O'Halloran, P., Macdonald, H., & Holland, A. E. (2021). Telerehabilitation for chronic respiratory disease. *The Cochrane database of systematic reviews*, *1*(1), CD013040. https://doi.org/10.1002/14651858.CD013040.pub2

| Database: Medline via Ovid, version: MEDLINE®ALL | |
| --- | --- |
| concept | string |
| telerehabilitation | 1. (Telemedicine or Distance Counseling or Mental Health Teletherapy or Telerehabilitation or Remote Consultation or Digital Health or Telemetry or Remote Sensing Technology or Telephone or Videoconferencing or Webcasts as Topic or Cell Phone or Smartphone or Computers, Handheld or Microcomputers or Text Messaging or Electronic Mail or Internet-Based Intervention or Fitness Trackers or Wearable Electronic Devices or Video Games or Exergaming or Mobile Applications or Gamification).sh 2. (telemedic* or tele-medic* or telemetry or telerehab* or tele-rehab* or telehealth or tele-health or telecare or tele-care or telecoaching or tele-coaching or tele-exercis* or tele-educat* or teletherap* or tele-therap*).tw. 3. (ehealth or e-health or mobile health or mhealth or m-health or digital health).tw. 4. ((remote* or distanc* or distant* or online or virtual or digital* or telephone* or phone* or video* or internet* or computer* or web* or email* or e-mail* or text messag* or cellphone* or cell-phone* or app* or smartphone* or exergam* or game or gaming or fitness tracker? or activity tracker?) adj3 (rehab* or therap* or treat* or exercis* or care or intervention or educat*)).tw. 5. or/1-4 |
| diagnoses upper extremity | 1. exp Shoulder Injuries/ or exp Arm Injuries/ or exp Hand Injuries/ or exp Median Neuropathy/ or exp Ulnar Neuropathies/ or exp Ulnar Nerve Compression Syndromes/ or exp Elbow Tendinopathy/ or Radial Neuropathy.sh. or tennis-elbow.tw. or adhesive capsulitis.tw. or frozen shoulder.tw. or carpal-tunnel syndrome.tw. or cubital-tunnel syndrome.tw. or rhizarthrosis.tw. or dupuytren.tw. 2. exp Upper Extremity/ or exp Bones of Upper Extremity/ or exp Hand Joints/ or Rotator Cuff.sh. or Elbow Joint.sh. or Scapula.sh. or Shoulder Joint.sh. or Sternoclavicular Joint.sh. or Sternocostal Joints.sh. or Acromioclavicular Joint.sh. or (arm not (one-arm or two-arm or single-arm or study-arm or intervention-arm)).tw. or (arms not (one-arms or two-arms or single-arms or study-arms or intervention-arms)).tw. or carpal*.tw. or carpus.tw. or clavicle.tw. or clavicula*.tw. or elbow?.tw. or finger?.tw. or forearm*.tw. or hand.tw. or hands.tw. or humeral.tw. or humeri.tw. or humerus.tw. or metacarp*.tw. or radial*.tw. or radius.tw. or rotator-cuff.tw. or scapula*.tw. or shoulder*.tw. or subacromial.tw. or thumb*.tw. or ulna*.tw. or upper-extremit*.tw. or upper-limb?.tw. or wrist?.tw. 3. exp Musculoskeletal Diseases/ or exp Orthopedic Procedures/ or exp Arthroscopy/ or exp Arthroplasty, Replacement/ or (Orthopedics or Occupational Injuries).sh. or arthrosis.tw. or arthritis.tw. or arthroses.tw. or arthritides.tw. or osteoarthrosis.tw. or osteoarthroses.tw. or osteoarthritis.tw. or musculoskeletal.tw. or muskuloskeletal.tw. or injur*.tw. or trauma.tw. or fracture.tw. or surgery.tw. or arthroplast*.tw. or arthroscop*.tw. or tendinopathy.tw. or impingement.tw. or epicondylitis.tw. or bursitis.tw. or tendinitis.tw. or post-operativ*.tw. or postoperativ*.tw. or compression neuropath*.tw. or neural compression.tw. or nerv* compression.tw. or compression syndrome?.tw. 4. 6 or (7 adj3 8) |
| controlled trial | 1. randomized controlled trial.pt. 2. controlled clinical trial.pt. 3. pragmatic clinical trial.pt. 4. multicenter study.pt. 5. Non Randomized Controlled Trials As Topic.sh. 6. (randomis* or randomiz* or randomly).tw. 7. groups.ab. 8. (trial or multicenter or multi center or multicentre or multi centre).ti. 9. (intervention? or effect? or impact? or controlled or control group? or (before adj5 after) or (pre adj5 post) or ((pretest or pre test) and (posttest or post test)) or quasiexperiment* or quasi experiment* or evaluat*).tw. 10. exp Animals/ 11. Humans.sh. 12. 19 not (19 and 20) 13. review.pt. 14. meta analysis.pt. 15. news.pt. 16. comment.pt. 17. editorial.pt. 18. cochrane database of systematic reviews.jn. 19. comment on.cm. 20. (systematic review or literature review).ti. 21. (case study or case report or case series).ti. 22. or/21-30 23. or/10-18 24. 32 not 31 |
|  | 1. 5 and 9 and 33 |
|  | 1. limit 34 to yr="2000 -Current" |

**References for Embase (via Ovid) string:**

For concept “controlled trial” the search string was adopted with small adjustments from:

Lefebvre C, Glanville J, Briscoe S, Featherstone R, Littlewood A, Metzendorf M-I, Noel-Storr A, Paynter R, Rader T, Thomas J, Wieland LS. Chapter 4: Searching for and selecting studies [last updated March 2025]. In: Higgins JP, Thomas J, Chandler J, Cumpston M, Li T, Page MJ, et al, editor(s). Cochrane Handbook for Systematic Reviews of Interventions version 6.5.1 Cochrane, 2025. Available from *cochrane.org/handbook*.

For concept “telerehabilitation” the search string was partly adopted from:

Cox, N. S., Dal Corso, S., Hansen, H., McDonald, C. F., Hill, C. J., Zanaboni, P., Alison, J. A., O'Halloran, P., Macdonald, H., & Holland, A. E. (2021). Telerehabilitation for chronic respiratory disease. *The Cochrane database of systematic reviews*, *1*(1), CD013040. https://doi.org/10.1002/14651858.CD013040.pub2

| Database: Embase via Ovid, version: Embase 1974 to 2024 November 26 | |
| --- | --- |
| concept | string |
| telerehabilitation | 1. (telemedicine or telehealth or teleconsultation or telediagnosis or telemonitoring or telepsychology or telerehabilitation or teletherapy or video consultation or e-counseling or telemetry or remote sensing or digital health or digital health technology or telephone or teleconsultation or electronic consultation or videoconferencing or webcast or smartphone or mobile phone or smart device or microcomputer or minicomputer tablet computer or wearable device or wearable sensor or smart watch or wrist-worn-device or wearable computer or activity tracker or actigraph or pedometer or wrist-worn accelerometer or text messaging or e-mail or web-based intervention or video game or exergaming or mobile health application or self-care software).sh. 2. (telemedic* or tele-medic* or telemetry or telerehab* or tele-rehab* or telehealth or tele-health or telecare or tele-care or telecoaching or tele-coaching or tele-exercis* or tele-educat* or teletherap* or tele-therap* videoconferenc* or video-conferenc* or videoconsultation or video-consultation).tw. 3. (ehealth or e-health or mobile health or mhealth or m-health or digital health).tw. 4. ((remote* or distanc* or distant* or online or virtual or digital* or telephone* or phone* or video* or internet* or computer* or web* or email* or e-mail* or text messag* or cellphone* or cell-phone* or app* or smartphone* or exergam* or game or gaming or fitness tracker? or activity tracker?) adj3 (rehab* or therap* or treat* or exercis* or care or intervention or educat*)).tw. 5. or/1-4 |
| diagnoses upper extremity | 1. exp arm fracture/ or exp rotator cuff injury/ or exp shoulder injury/ or exp arm injury/ or exp arm disease/ or exp shoulder surgery/ or exp wrist surgery/ or exp elbow arthroplasty/ or exp finger arthroplasty/ or exp shoulder pain/ or tennis-elbow.tw. or adhesive capsulitis.tw. or frozen shoulder.tw. or carpal-tunnel syndrome.tw. or cubital-tunnel syndrome.tw. or dupuytren.tw. or rhizarthrosis.tw. 2. exp upper limb/ or (arm not (one-arm or two-arm or single-arm or study-arm or intervention-arm)).tw. or (arms not (one-arms or two-arms or single-arms or study-arms or intervention-arms)).tw. or carpal*.tw. or carpus.tw. or clavicle.tw. or clavicula*.tw. or elbow?.tw. or finger?.tw. or forearm*.tw. or hand.tw. or hands.tw. or humeral.tw. or humeri.tw. or humerus.tw. or metacarp*.tw. or radial*.tw. or radius.tw. or rotator-cuff.tw. or scapula*.tw. or shoulder*.tw. or subacromial.tw. or thumb*.tw. or ulna*.tw. or upper-extremit*.tw. or upper-limb?.tw. or wrist?.tw. 3. exp orthopedic surgery/ or exp occupational disease/ or exp musculoskeletal disease/ or (orthopedics or nerve compression).sh. or arthrosis.tw. or arthritis.tw. or arthroses.tw. or arthritides.tw. or osteoarthrosis.tw. or osteoarthroses.tw. or osteoarthritis.tw. or musculoskeletal.tw. or muskuloskeletal.tw. or injur*.tw. or trauma.tw. or fracture.tw. or surgery.tw. or tendinopathy.tw. or tendinitis.tw. or impingement.tw. or epicondylitis.tw. or bursitis.tw. or arthroplast*.tw. or arthroscop*.tw. or post-operativ*.tw. or postoperativ*.tw. or compression neuropath*.tw. or nerv* compression.tw. or neural compression.tw. or compression syndrome?.tw. 4. 6 or (7 adj3 8) |
| controlled trial | 1. exp randomized controlled trial/ 2. controlled clinical trial.sh. 3. random*.tw. 4. randomization.sh. 5. intermethod comparison.sh. 6. placebo.tw. 7. (compare or compared or comparison).ti. 8. ((evaluated or evaluate or evaluating or assessed or assess) and (compare or compared or comparing or comparison)).ab. 9. (open adj label).tw. 10. ((double or single or doubly or singly) adj (blind or blinded or blindly)).tw. 11. double blind procedure.sh. 12. parallel group?.tw. 13. (crossover or cross over).tw. 14. ((assign* or match or matched or allocation) adj5 (alternate or group? or intervention? or patient? or subject? or participant?)).tw. 15. (assigned or allocated).tw. 16. (controlled adj7 (study or design or trial)).tw. 17. controlled study.sh. 18. pretest posttest control group design.sh. 19. (volunteer or volunteers).tw. 20. human experiment.sh. 21. trial.ti. 22. (intervention? or effect? or impact? or controlled or control group? or (before adj5 after) or (pre adj5 post) or ((pretest or pre test) and (posttest or post test)) or quasiexperiment* or quasi experiment* or evaluat*).tw. 23. or/10-31 24. (random* adj sampl* adj7 ("cross section*" or questionnaire? or survey* or database?)).tw. not ((comparative study or controlled study).sh. or randomi?ed controlled.tw. or randomly assigned.tw.) 25. cross-sectional study.sh. not (exp randomized controlled trial/ or controlled clinical trial.sh. or controlled study.sh. or randomi?ed controlled.tw. or control group?.tw.) 26. (((case adj control*) and random*) not randomi?ed controlled).tw. 27. systematic review.tw. not (trial or study).ti. 28. "random field*".tw. 29. (random cluster adj3 sample*).tw. 30. (review.ab. and review.pt.) not trial.ti. 31. "we searched".ab. and (review.ti. or review.pt.) 32. "update review".ab. 33. (databases adj4 searched).ab. 34. (rat or rats or mouse or mice or swine or porcine or murine or sheep or lambs or pigs or piglets or rabbit or rabbits or cat or cats or dog or dogs or cattle or bovine or monkey or monkeys or trout or marmoset?).ti. and animal experiment.sh. 35. (animal experiment not (human experiment or human)).sh. 36. (case study or case report or case series).ti. 37. or/33-45 38. 32 not 46 |
|  | 1. 5 and 9 and 47 |
|  | 1. limit 48 to yr="2000 -Current" |

**References for AMED (via Ovid) string:**

See references for Medline and Embase string.

| Database: AMED (Allied and Complementary Medicine) 1985 to October 2024 | |
| --- | --- |
| concept | string |
| telerehabilitation | 1. (Telemedicine or Telephone or Internet or Virtual Reality or Expert Systems or Therapy Computer Assisted).sh. or exp Computers/ 2. (telemedic* or tele-medic* or telemetry or telerehab* or tele-rehab* or telehealth or tele-health or telecare or tele-care or telecoaching or tele-coaching or tele-exercis* or tele-educat* or teletherap* or tele-therap*).tw. 3. (ehealth or e-health or mobile health or mhealth or m-health or digital health).tw. 4. ((remote* or distanc* or distant* or online or virtual or digital* or telephone* or phone* or video* or internet* or computer* or web* or email* or e-mail* or text messag* or cellphone* or cell-phone* or app* or smartphone* or exergam* or game or gaming or fitness tracker? or activity tracker?) adj3 (rehab* or therap* or treat* or exercis* or care or intervention or educat*)).tw. 5. or/1-4 |
| diagnoses upper extremity | 1. exp Arm Injuries/ or exp Hand Injuries/ or exp Radius Fractures/ or Shoulder Dislocation.sh. or Tennis Elbow.sh. or Dupuytrens Contracture.sh. or Shoulder Pain.sh. or Carpal Tunnel Syndrome.sh. or tennis-elbow.tw. or adhesive capsulitis.tw. or frozen shoulder.tw. or carpal-tunnel syndrome.tw. or cubital tunnel syndrome.tw. or rhizarthrosis.tw. or dupuytren.tw. 2. exp Arm/ or exp Arm Bones/ or exp Hand Bones/ or exp Brachial Plexus/ or Elbow Joint.sh. or Hand Joint.sh. or Shoulder Joint.sh. or Rotator Cuff.sh. or (arm not (one-arm or two-arm or single-arm or study-arm or intervention-arm)).tw. or (arms not (one-arms or two-arms or single-arms or study-arms or intervention-arms)).tw. or carpal*.tw. or carpus.tw. or clavicle.tw. or clavicula*.tw. or elbow?.tw. or finger?.tw. or forearm?.tw. or hand.tw. or hands.tw. or humeral.tw. or humeri.tw. or humerus.tw. or metacarp*.tw. or radial*.tw. or radius.tw. or rotator-cuff.tw. or scapula*.tw. or shoulder*.tw. or subacromial.tw. or thumb*.tw. or ulna*.tw. or upper-extremit*.tw. or upper-limb?.tw. or wrist?.tw. 3. exp Orthopedics/ or Occupational Injuries.sh. or exp Musculoskeletal Diseases/ or Arthroscopy.sh. or arthrosis.tw. or arthritis.tw. or arthroses.tw. or arthritides.tw. or osteoarthrosis.tw. or osteoarthroses.tw. or osteoarthritis.tw. or musculoskeletal.tw. or muskuloskeletal.tw. or injur*.tw. or trauma.tw. or fracture.tw. or surgery.tw. or arthroplast*.tw. or arthroscop*.tw. or tendinopathy.tw. or impingement.tw. or epicondylitis.tw. or bursitis.tw. or tendinitis.tw. or post-operativ*.tw. or postoperativ*.tw. or compression neuropath*.tw. or neural compression.tw. or nerv* compression.tw. or compression syndrome?.tw. 4. 6 or (7adj3 8) |
| controlled trial | 1. randomized controlled trial.pt. 2. controlled clinical trial.pt. 3. multicenter study.pt. 4. (clinical trials or randomized controlled trials or random allocation).sh. 5. (randomis* or randomiz* or randomly).tw. 6. groups.ab. 7. (trial or multicenter or multi center or multicentre or multi centre).ti. 8. (intervention? or effect? or impact? or controlled or control group? or (before adj5 after) or (pre adj5 post) or ((pretest or pre test) and (posttest or post test)) or quasiexperiment* or quasi experiment* or evaluat*).tw. 9. trial.ti. 10. (crossover or cross over).tw. 11. (compare or compared or comparison).ti. 12. ((evaluated or evaluate or evaluating or assessed or assess) and (compare or compared or comparing or comparison)).ab. 13. (open adj label).tw. 14. ((double or single or doubly or singly) adj (blind or blinded or blindly)).tw. 15. parallel group?.tw. 16. ((assign* or match or matched or allocation) adj5 (alternate or group or intervention? or patient? or subject? or participant?)).tw. 17. (assigned or allocated).tw. 18. (controlled adj7 (study or design or trial)).tw. 19. (volunteer or volunteers).tw. 20. or/10-28 21. exp Animals 22. Humans.sh. 23. 30 not 31 24. (random* adj sampl*).tw. adj7 (("cross section*" or questionnaire? or survey* or database?).tw. not (Comparative Study/ or Controlled Study/ or randomi?ed controlled.tw. or randomly assigned.tw.)) 25. (((case adj control*) and random*) not randomi?ed controlled).tw. 26. (systematic review not (trial or study)).ti. 27. (nonrandom* not random*).tw. 28. (random cluster adj3 sampl*).tw. 29. (review.ab. and review.pt.) not trial.ti. 30. "we searched".ab. and (review.ti. or review.pt.) 31. "update review".ab. 32. (databases adj4 searched).ab. 33. meta analysis.pt. 34. news.pt. 35. comment.pt. 36. editorial.pt. 37. (systematic review or literature review).ti. 38. (case study or case report or case series).ti. 39. or/32-47 40. 29 not 48 |
|  | 1. 5 and 9 and 49 |
|  | 1. limit 50 to yr="2000 -Current" |
