# Supplementary material for: The effectiveness of telerehabilitation in upper limb musculoskeletal disorders: a systematic review
Source: BMC Musculoskelet Disord. 2026 May 28;27:462. doi: 10.1186/s12891-026-10008-7 (PMC13220470; doi:10.1186/s12891-026-10008-7)
Supplement: Supplementary file 3 — Additional file 3: List of exclusions during full text screen: List of excluded studies within full-text screening along with reason for exclusion and reference. [file 12891_2026_10008_MOESM3_ESM.docx]

**List of exclusions during full text screen**

| **Reference** | **Reason for exclusion** |
| --- | --- |
| Albanese et al., 2021 | Intervention not remote |
| Albiol-Pérez et al., 2016 | Intervention not remote |
| Anan T. et al., 2021 | Not upper extremity diagnosis |
| Anthony C.A. et al., 2020 | Not upper extremity diagnosis |
| Arman et al., 2024 | Intervention not remote |
| Bateman et al., 2024 | Intervention criteria not met |
| Blasco J.M. et al., 2024 | Abstract only - full text not retrieved |
| Bressler et al., 2024 | Intervention not remote |
| Calner et al., 2017 | Not upper extremity diagnosis |
| Carbonaro et al., 2018 | Intervention criteria not met |
| Celik & Tuncer, 2024 | Not high-income country |
| Daf & Patil, 2024 | Intervention not remote |
| Dahl-Popolizio et al., 2014 | Intervention not remote |
| de Oliveira A.K.A. et al., 2022 | Intervention not remote |
| Eberl et al., 2006 | Intervention criteria not met |
| Eriksson L et al., 2009 | Diagnoses included rheumatoid arthritis |
| Eriksson L. et al., 2011 | Diagnoses included rheumatoid arthritis |
| Fabres Martin et al., 2023 | Intervention not remote |
| Ganjiwale D. et al., 2019 | Not high-income country |
| Gilmer G. et al., 2024 | Intervention criteria not met |
| Gunawan D. et al., 2020 | Intervention not remote |
| Haidamous G. et al., 2023 | Abstract only - full text not retrieved |
| Holmgren et al., 2012 | Intervention criteria not met |
| Hopewell et al., 2021 | Intervention criteria not met |
| Horoz et al., n.d. | Not high-income country |
| Hsu et al., 2011 | Intervention criteria not met |
| Hutting et al., 2015 | Diagnosis criteria not met |
| Inal & Tuncer, 2022 | Not high-income country |
| Jha et al., 2024 | Intervention criteria not met |
| Kanat et al., 2024 | Not high-income country |
| Kane et al., 2020 | Intervention criteria not met |
| Kapsigay B. et al., 2017 | Abstract only - full text not retrieved |
| Keulers et al., 2007 | Intervention criteria not met |
| Khoshrounejad et al., 2022 | Not high-income country |
| Kosterink et al., 2010 | Diagnosis criteria not met |
| Kroger et al., 2021 | Intervention not remote |
| Lambert et al., 2017 | Diagnosis criteria not met |
| Larsman et al., 2010 | Diagnosis criteria not met |
| Lin et al., 2024 | Study design not controlled |
| Malliaras et al., 2020 | comparison also remote |
| Matamala-Gomez et al., 2022 | Intervention not remote |
| Menek et al., 2022 | Intervention not remote |
| Miller J.S. et al., 2004 | Intervention criteria not met |
| Muschol et al., 2023 | Intervention criteria not met |
| Muschol J. et al., 2022 | Intervention criteria not met |
| Naqvi et al., 2022 | Not high-income country |
| Naqvi W. & Quershi M.I., 2024 | Abstract only - full text not retrieved |
| Nica A.S. et al., 2013 | Intervention not remote |
| Nordin et al., 2016 | Diagnosis criteria not met |
| Nunez-Cortes et al., 2023 | Intervention criteria not met |
| O’Donnell et al., 2022 | Intervention criteria not met |
| Ongvisatepaiboon K. et al., 2016 | Intervention not remote |
| Osteras N. et al., 2014 | Intervention criteria not met |
| Ozlu et al., 2024 | Intervention not remote |
| Parel et al., 2023 | Intervention not remote |
| Parwaiz et al., 2022 | Intervention criteria not met |
| Pech-Arguelles R.C. et al., 2024 | Not German or English language |
| Pekyavas & Ergun, 2017 | Intervention criteria not met |
| Pekyavas N.O. & Ergun N., 2014 | Intervention criteria not met |
| Provinciali L et al., 2000 | Intervention criteria not met |
| Rizzato A. et al., 2023 | Intervention not remote |
| Rizzo et al., 2017 | Intervention not remote |
| Sabbagh et al., 2023 | Intervention criteria not met |
| Salisbury et al., 2013 | Diagnosis criteria not met |
| Sandsjo L. et al., 2010 | Diagnosis criteria not met |
| Santello et al., 2020 | Intervention criteria not met |
| Schick et al., 2023 | Intervention criteria not met |
| Schmidt et al., 2024 | Diagnosis criteria not met |
| Schwartz et al., 2022 | Intervention criteria not met |
| Semjonova et al., 2020 | Intervention criteria not met |
| Seven et al., 2024 | Not high-income country |
| Sharma R. et al., 2024 | Intervention criteria not met |
| Sveistrup et al., 2003 | Intervention not remote |
| Then et al., 2020 | Not high-income country |
| Tokgoz et al., 2023 | Intervention not remote |
| Tsvyakh et al., 2021 | Not high-income country |
| Tsvyakh A.I. & Hospodarskyy A.J., 2019 | Abstract only - full text not retrieved |
| Tsvyakh & Hospodarskyy, 2018 | Abstract only - full text not retrieved |
| Turkmen et al., 2020 | Not high-income country |
| van den Heuvel et al., 2003 | Intervention criteria not met |
| van Eck C. et al., 2018 | Intervention criteria not met |
| Walter et al., 2023 | Study design not controlled |
| Wiederhold B.K. & Wiederhold M.D., 2006 | Intervention not remote |
| Xiao X. et al., 2018 | Intervention not remote |
| Clark et al., 2019 | Intervention: IG received telerehabilitation + coping intervention in person, therefore no isolated effect of teleintervention |
| Anderson et al., 2021 | No primary outcome reported |
| Svingen et al., 2021 | No primary outcome reported |
| Chen et al., 2020 | NRSI -> excluded within ROBINS-I V2 assessment, see supplemental material 4 |
| Mayer et al., 2021 | NRSI -> excluded within ROBINS-I V2 assessment, see supplemental material 4 |
| Palm et al., 2023 | NRSI -> excluded within ROBINS-I V2 assessment, see supplemental material 4 |
| Suero-Pineda et al., 2023 | NRSI -> excluded within ROBINS-I V2 assessment, see supplemental material 4 |

NRSI: non randomized studies of interventions, ROBINS-I: ROBINS-I V2: The Risk of Bias in Non-randomized Studies – of Interventions Version 2, IG: intervention group

**References**

Albanese GA, Taglione E, Gasparini C, Grandi S, Pettinelli F, Sardelli C, Catitti P, Sandini G, Masia L, & Zenzeri J. Efficacy of wrist robot-aided orthopedic rehabilitation: A randomized controlled trial. Journal of neuroengineering and rehabilitation. 2021; doi: 10.1186/s12984-021-00925-0

Albiol-Pérez S, Mena-Cajas J, Escobar-Anchaguano IP, Pruna-Panchi EP & Zumbana P. Virtual fine rehabilitation in patients with carpal tunnel syndrome using low-cost devices. In Proceedings of the 4th Workshop on ICTs for Improving Patients Rehabilitation Research Techniques. 2016. pp. 61-64.

Anan T, Kajiki S, Oka H, Fujii T, Kawamata K, Mori K, & Matsudaira K. Effects of an Artificial Intelligence-Assisted Health Program on Workers With Neck/Shoulder Pain/Stiffness and Low Back Pain: Randomized Controlled Trial. JMIR mHealth and uHealth. 2021; doi: 10.2196/27535.

Anderson L, Kinsman S, & Oberlander M. Postoperative Compliance and Return to Work After Rotator Cuff Repair: Value of an Interactive Online Rehabilitation Program Among Patients Treated Under Workers’ Compensation. Orthopedics. 2021; doi: 10.3928/014774477-20201119-07.

Anthony CA, Rojas EO, Keffala V, Glass NA, Shah AS, Miller BJ, Hogue M, Willey MC, Karam M, & Marsh JL. Acceptance and commitment therapy delivered via a mobile phone messaging robot to decrease postoperative opioid use in patients with orthopedic trauma: Randomized controlled trial. Journal of Medical Internet Research. 2020; doi: 10.2196/17750.

Arman S, Menekseoglu AK, Sezgin B, Ozgur B, Capan N, & Oral A. The effects of virtual reality-mediated tendon and nerve gliding exercises in the conservative management of carpal tunnel syndrome: A double-blind randomized placebo controlled trial. European journal of physical and rehabilitation medicine. 2024; doi: 10.23736/S1973-9087.24.08432-6.

Bateman M, Skeggs A, Whitby E, Fletcher-Barrett V, Stephens G, Dawes M, Davis D, Beckhelling J, Cooper K, Saunders B, Littlewood C, Vicenzino B, Foster NE, & Hill JC. Optimising physiotherapy for people with lateral elbow tendinopathy—Results of a mixed-methods pilot and feasibility randomised controlled trial (OPTimisE). Musculoskeletal science & practice. 2024; doi: 10.1016/j.msksp.2023.102905.

Blasco J.M., Diaz-Diaz B., Perez-Maletzki J., Hernandez-Guillen D., Navarro-Bosch M., Aroca J.-E., & Roig-Casasus S. A natural language processing tool to promote adherence to home rehabilitation after major joint replacement surgeries in osteoarthritis. Osteoarthritis and Cartilage, 2024; doi: 10.1016/j.joca.2024.03.089.

Bressler M, Merk J, Gohlke T, Kayali F, Daigeler A, Kolbenschlag J, & Prahm C. A Virtual Reality Serious Game for the Rehabilitation of Hand and Finger Function: Iterative Development and Suitability Study. JMIR serious games. 2024; doi: 10.2196/54193.

Calner T, Nordin C, Eriksson MK, Nyberg L, Gard G, & Michaelson P. Effects of a self-guided, web-based activity programme for patients with persistent musculoskeletal pain in primary healthcare: A randomized controlled trial. European journal of pain. 2017; doi: 10.1002/ejp.1012.

Carbonaro N, Lucchesi I, Lorusssi F & Tognetti A. Tele-monitoring and tele-rehabilitation of the shoulder muscular-skeletal diseases through wearable systems. Annual International Conference of the IEEE Engineering in Medicine and Biology Society. IEEE Engineering in Medicine and Biology Society. Annual International Conference. 2018; doi: 10.1109/EMBC.2018.8513371.

Celik EB & Tuncer A. Comparing the Efficacy of Manual Therapy and Exercise to Synchronized Telerehabilitation with Self-Manual Therapy and Exercise in Treating Subacromial Pain Syndrome: A Randomized Controlled Trial. Healthcare. 2024; doi: 10.3390/healthcare12111074.

Clark H, Bassett S & Siegert R. The effectiveness of web-based patient education and action and coping plans as an adjunct to patient treatment in physiotherapy: A randomized controlled trial. Physiotherapy Theory and Practice. 2019; doi: 10.1080/09593985.2018.1467521.

Daf A & Patil DS. Impact of Virtual Rehabilitation in Adjunct to Conventional Physical Therapy on Proximal Humerus Fracture: A Randomized Controlled Trial. Cureus. 2024; doi: 10.7759/cureus.56022.

Dahl-Popolizio S, Loman J & Cordes CC. Comparing Outcomes of Kinect Videogame-Based Occupational/Physical Therapy Versus Usual Care. Games for health journal. 2014; doi: 10.1089/g4h.2014.0002.

de Oliveira AKA, da Costa KSA, de Lucena GL, de Oliveira Sousa C, Filho JFM, & Brasileiro JS. Comparing exercises with and without electromyographic biofeedback in subacromial pain syndrome: A randomized controlled trial. Clinical Biomechanics. 2022; doi: 10.1016/j.clinbiomech.2022.105596.

Eberl R, Kaminski A, Reckwitz N, Muhr G & Clasbrummel B. [The tele-visit as a telemedical technique in daily clinical practice. First results for elbow joint arthrolysis]. Der Unfallchirurg. 2006; doi: 10.1007/s00113-006-1062-0.

Eriksson L, Ekenberg L, Gard G, Lindstrom B & Lysholm J. Effects and patients’ experiences of telerehabilitation at home after shoulder joint replacement. Physiotherapy. 2011; doi: 10.1016/j.physio.2011.04.003.

Fabres Martin C, Ventura Parellada C, Herrero Anton de Vez H, Ordonez Urgiles CE, Alonso-Rodriguez Piedra J & Mora Guix JM. Telemedicine approach for patient follow-up after total knee and reverse total shoulder arthroplasty: A pilot study. International journal of computer assisted radiology and surgery. 2023; doi: 10.1007/s11548-022-02784-z.

Ganjiwale D, Pathak R, Dwivedi A, Ganjiwale J & Parekh S. Occupational therapy rehabilitation of industrial setup hand injury cases for functional independence using modified joystick in interactive computer gaming in Anand, Gujarat. National Journal of Physiology, Pharmacy and Pharmacology. 2019; doi: 10.5455/njppp.2019.9.06202112018001.

Gilmer G, Cluts L, Gibbons JL, & Fowler J. A Randomized-Controlled Trial Evaluating the Impact of a Web Tutorial on Perceptions and Usage of Opioids Post-Carpal Tunnel Release Surgery. Journal of Hand Surgery Global Online. 2024; doi: 10.1016/j.jhsg.2024.03.010.

Gunawan D, Kusharyaningsih RH & Handajani NI. Association between stretching exercise with virtual reality game and over head pulley of frozen shoulder patients Indian Journal of Forensic Medicine and Toxicology. 2020. 14(2), 1507EP – 1512

Haidamous G, Couchara L, Simon P, Christmas K, Lee W, Jensen M, & Frankle M. Effects of hypnosis therapy on pain and opioid use following shoulder replacement surgery. JSES International. 2023; doi: 101016/jjseint202306003.

Holmgren T, Oberg B, Sjoberg I & Johansson K. Supervised strengthening exercises versus home-based movement exercises after arthroscopic acromioplasty: A randomized clinical trial Journal of rehabilitation medicine. 2012; doi: 102340/16501977-0889.

Hopewell S, Keene DJ, Marian IR, Dritsaki M, Heine P, Cureton L, Dutton SJ, Dakin H, Carr A, Hamilton W, Hansen Z, Jaggi A, Littlewood C, Barker KL, Gray A & Lamb SE. Progressive exercise compared with best practice advice, with or without corticosteroid injection, for the treatment of patients with rotator cuff disorders (GRASP): A multicentre, pragmatic, 2 x 2 factorial, randomised controlled trial Lancet. 2021; doi; 101016/S0140-6736(21)00846-1.

Horoz L, Karaçay BÇ, Ceylan İ & Çakmak MF. Is home-based real-time video conferencing telerehabilitation as effective as conventional face-to-face rehabilitation in patients with operated for distal radius fracture? A single-blind, randomized prospective study. Turkish Journal of Physical Medicine and Rehabilitation. 2024, 70(4), 506.

Hsu JK, Thibodeau R, Wong SJ, Zukiwsky D, Cecile S & Walton DM. A „Wii“ bit of fun: The effects of adding Nintendo Wii( R) Bowling to a standard exercise regimen for residents of long-term care with upper extremity dysfunction Physiotherapy theory and practice. 2011; doi: 103109/09593985201048326727.

Hutting N, Staal JB, Engels JA, Heerkens YF, Detaille SI & Nijhuis-van der Sanden, MWG. Effect evaluation of a self-management programme for employees with complaints of the arm, neck or shoulder: A randomised controlled trial Occupational and environmental medicine. 2015; doi: 101136/oemed-2015-103089.

Inal , & Tuncer B. Telephone-based joint protection education in lateral epicondylitis: A randomized controlled trial Work. 2022; doi: 103233/WOR-211002.

Jha CK, Shukla Y, Mukherjee R, Rathva P, Joshi M & Jain D. A Glove-Based Virtual Hand Rehabilitation System for Patients With Post-Traumatic Hand Injuries. IEEE transactions on bio-medical engineering. 2024; doi: 101109/TBME20243360888.

Kanat C, Ugras GA, Unal R, Donmez SC, Tasdelen B & Oztuna FV. The effect of video-assisted training on upper extremity problems and functions after rotator cuff repair: A randomized controlled trial Turkish journal of medical sciences. 2024; doi: 1055730/1300-01445777.

Kane LT, Thakar O, Jamgochian G, Lazarus MD, Abboud JA, Namdari S & Horneff JG. The role of telehealth as a platform for postoperative visits following rotator cuff repair: A prospective, randomized controlled trial Journal of shoulder and elbow surgery. 2020; doi: 101016/jjse201912004.

Kapsigay B, Sari Z, Kavlak B, Aras I & Tanhan A. HPR effects of virtual rehabilitation on shoulder periarthritis Annals of the Rheumatic Diseases. 2017; doi: 101136/annrheumdis-2017-eular5772.

Keulers BJ, Welters CFM, Spauwen PHM & Houpt P. Can face-to-face patient education be replaced by computer-based patient education? A randomised trial Patient education and counseling. 2007; doi: 101016/jpec200703012.

Khoshrounejad F, Tabesh H, Aalaei S, Khoshrounejad S, Moradi A & Eslami S. Effect of a Text Message-Based Support Program on Outcomes of Patients After Flexor Tendon Injury Repair The Journal of hand surgery. 2022; doi: 101016/jjhsa202207012.

Kosterink SM, Huis in ’t Veld RMHA, Cagnie B, Hasenbring M & Vollenbroek-Hutten MMR. The clinical effectiveness of a myofeedback-based teletreatment service in patients with non-specific neck and shoulder pain: A randomized controlled trial Journal of telemedicine and telecare. 2010; doi: 101258/jtt2010006005.

Kroger I, Nerz C, Schwickert L, Scholch S, Musig JA, Studier-Fischer S, Nolte PC, Becker C & Augat P. Robot-assisted training after proximal humeral fracture: A randomised controlled multicentre intervention trial Clinical rehabilitation. 2021; doi: 101177/0269215520961654.

Lambert TE, Harvey LA, Avdalis C, Chen LW, Jeyalingam S, Pratt CA, Tatum HJ, Bowden JL & Lucas BR . An app with remote support achieves better adherence to home exercise programs than paper handouts in people with musculoskeletal conditions: A randomised trial Journal of physiotherapy. 2017; doi: 101016/jjphys201705015.

Larsman P, Hasenbring M, Sandsjo L, Huis in ’t Veld RMHA, Witvrouw E, Kosterink SM, Kadefors R & Vollenbroek-Hutten MMR. Prognostic factors for the effect of a myofeedback-based teletreatment service Journal of telemedicine and telecare. 2010; doi: 101258/jtt2010006008.

Lin YJ, Chung CY, Chen CPC, Hsieh YW, Wang CF & Chen CC. The Feasibility and Efficacy of Remote App-Guided Home Exercises for Frozen Shoulder: A Pilot Study Healthcare. 2024; doi: 103390/healthcare1211109.

Malliaras P, Cridland K, Hopmans R, Ashton S, Littlewood C, Page R, Harris I, Skouteris H & Haines T. Internet and Telerehabilitation-Delivered Management of Rotator Cuff-Related Shoulder Pain (INTEL Trial): Randomized Controlled Pilot and Feasibility Trial JMIR mHealth and uHealth. 2020; doi: 102196/24311

Matamala-Gomez M, Slater M & Sanchez-Vives MV. Impact of virtual embodiment and exercises on functional ability and range of motion in orthopedic rehabilitation Scientific reports. 2022; doi: 101038/s41598-022-08917-3.

Mayer N, Portnoy S, Palti R & Levanon Y. The efficacy of tele-rehabilitation program for improving upper limb function among adults following elbow fractures: A pilot study Applied Sciences. 2021, 11(4), 1708

Menek B, Tarakci D, Tarakci E & Menek MY. Investigation on the Efficiency of the Closed Kinetic Chain and Video-Based Game Exercise Programs in the Rotator Cuff Rupture: A Randomized Trial Games for health journal. 2022; doi: 101089/g4h20210210.

Miller JS, Stanley I & Moore K. Videotaped exercise instruction: A randomised controlled trial in musculoskeletal physiotherapy Physiotherapy Theory and Practice. 2004; doi: 101080/09593980490487375.

Muschol J, Heinrich M, Heiss C, Hernandez AM, Knapp G, Repp H, Schneider H, Thormann U, Uhlar J, Unzeitig K & Gissel C. Digitization of Follow-Up Care in Orthopedic and Trauma Surgery With Video Consultations: Health Economic Evaluation Study From a Health Provider’s Perspective Journal of medical Internet research. 2023; doi: 102196/46714.

Muschol J, Heinrich M, Heiss C, Knapp G, Repp H, Schneider H, Thormann U, Uhlar J, Unzeitig K & Gissel C. Assessing Telemedicine Efficiency in Follow-up Care With Video Consultations for Patients in Orthopedic and Trauma Surgery in Germany: Randomized Controlled Trial Journal of Medical Internet Research. 2022; doi: 102196/36996.

Naqvi WM, Qureshi MI, Nimbulkar G & Umate L. Gamification for Distal Radius Fracture Rehabilitation: A Randomized Controlled Pilot Study Cureus. 2022; doi: 107759/cureus29333.

Naqvi W & Quershi MI. Impact of Gamification on Pain, Range of Motion, Muscle Strength, and Functional Independence Post Distal Radius Fracture Archives of Physical Medicine and Rehabilitation. 2024; doi: 101016/japmr202402575.

Nica AS, Brailescu CM & Scarlet RG. Virtual reality as a method for evaluation and therapy after traumatic hand surgery. Annual Review of Cybertherapy and Telemedicine. 2013, 48-52.

Nordin CA, Michaelson P, Gard G & Eriksson MK. Effects of the Web Behavior Change Program for Activity and Multimodal Pain Rehabilitation: Randomized Controlled Trial Journal of medical Internet research. 2016; doi: 102196/jmir5634.

Nunez-Cortes R, Cruz-Montecinos C, Torreblanca-Vargas S, Tapia C, Gutierrez-Jimenez M, Torres-Gangas P, Calatayud J & Perez-Alenda S. Effectiveness of adding pain neuroscience education to telerehabilitation in patients with carpal tunnel syndrome: A randomized controlled trial Musculoskeletal science & practice. 2023; doi: 101016/jmsksp2023102835.

O’Donnell EA, Haberli JE, Martinez AM, Yagoda D, Kaplan RS & Warner JJP. Telehealth Visits After Shoulder Surgery: Higher Patient Satisfaction and Lower Costs Journal of the American Academy of Orthopaedic Surgeons Global research & reviews. 2022; doi: 105435/JAAOSGlobal-D-22-00119.

Ongvisatepaiboon K, Vanijja V, Chignell M, Mekhora K & Chan JH. Smartphone-based audio-biofeedback system for shoulder joint tele-rehabilitation Journal of Medical Imaging and Health Informatics. 2016; doi: 101166/jmihi20161810.

Osteras N, Hagen K, Sand-Svartrud AL, Grotle M & Kjeken I. Exercises with telephone follow-up versus usual care in people with hand osteoarthritis: A randomised controlled trial Osteoarthritis and Cartilage. 2014; 22(SUPPL 1), S456.

Ozlu A, Ustundag S, Bulut Ozkaya D & Menekseoglu AK. Effect of Exergame on Pain, Function, and Quality of Life in Shoulder Impingement Syndrome: A Prospective Randomized Controlled Study Games for health journal. 2024; doi: 101089/g4h20230108.

Palm KB, Blazar PE, Manna JC, Serig AS, Phillips EA, Bay CP, Casey EJ & Earp BE. Feasibility, effectiveness and patient satisfaction of telerehabilitation after thumb carpometacarpal arthroplasty and reverse total shoulder arthroplasty: A pilot study Journal of Telemedicine and Telecare. 2023; doi: 101177/1357633X21999578.

Parel I, Padolino A, Candoli V, Filippi MV, Merolla G, Sanniti S, Paladini P & Cutti AG. Rehabilitation with Kinematic Biofeedback Improves Shoulder Function in Patients Surgically Treated for Rotator Cuff Tear: Indications from a Randomized Controlled Trial. 2023.

Parwaiz H, Trew C, Sheriff M & Langdon I. Patient Satisfaction with Face-to-Face Follow-Up versus Telephone Follow-Up after Elective Day Case Hand Surgery The journal of hand surgery Asian-Pacific volume. 2022; doi: 101142/S2424835522500072.

Pech-Arguelles RC, Miranda-Ortiz YJ, Velazquez-Hernandez HE, Dominguez-Cordero R, Ruiz-Pacheco C, Figueroa-Garcia J, & Rojano-Mejia D. Tele-rehabilitation program in patients with distal radius fracture: A controlled clinical trial Cirugia y Cirujanos. 2024; doi: 1024875/CIRU22000328.

Pekyavas NO & Ergun N. Comparison of virtual reality exergaming and home exercise programs in patients with subacromial impingement syndrome and scapular dyskinesis: Short term effect Acta orthopaedica et traumatologica turcica. 2017; doi: 101016/jaott201703008.

Pekyavas NO & Ergun N. Effects of different exercise and kinesiotaping application on pain, flexibility, strength and range of motion in patients with subacromial impingement syndrome Fizyoterapi Rehabilitasyon. 2014, 25(1 SUPPL 1), S31.

Provinciali L, Giattini A, Splendiani G & Logullo F. Usefulness of hand rehabilitation after carpal tunnel syndrome Muscle and Nerve. 2000, 23(2), 211–216.

Rizzato A, Pizzichemi M, Gobbi E, Gerardi A, Fortin C, Copcia A, Paoli A & Marcolin G. Effectiveness and therapeutic compliance of digital therapy in shoulder rehabilitation: A randomized controlled trial Journal of NeuroEngineering and Rehabilitation. 2023; doi: 101186/s12984-023-01188-7.

Rizzo JR, Thai P, Li EJ, Tung T, Hudson TE, Herrera J & Raghavan P. Structured Wii protocol for rehabilitation of shoulder impingement syndrome: A pilot study Annals of physical and rehabilitation medicine. 2017; doi: 101016/jrehab201610004.

Sabbagh R, Shah N, Jenkins S, Macdonald J, Foote A, Matar R, Steffensmeier A & Grawe B. The COVID-19 pandemic and follow-up for shoulder surgery: The impact of a shift toward telemedicine on validated patient-reported outcomes Journal of telemedicine and telecare. 2023; doi: 101177/1357633X21990997.

Salisbury C, Montgomery AA, Hollinghurst S, Hopper C, Bishop A, Franchini A, Kaur S, Coast J, Hall J, Grove S & Foster NE. Effectiveness of PhysioDirect telephone assessment and advice services for patients with musculoskeletal problems: Pragmatic randomised controlled trial BMJ. 2023; doi: 101136/bmjf43.

Sandsjo L, Larsman P, Huis in’t Veld RMHA & Vollenbroek-Hutten MMR. Clinical evaluation of a myofeedback-based teletreatment service applied in the workplace: A randomized controlled trial Journal of Telemedicine and Telecare. 2010; doi: 101258/jtt2010006007.

Santello G, Rossi DM, Martins J, Libardoni T de C & de Oliveira AS. Effects on shoulder pain and disability of teaching patients with shoulder pain a home-based exercise program: A randomized controlled trial Clinical rehabilitation. 2020; doi: 101177/0269215520930790.

Schick S, Elphingstone J, Paul K, He JK, Arguello A, Catoe B, Roberson T, Momaya A, Brabston E & Ponce B. Home-based physical therapy results in similar outcomes to formal outpatient physical therapy after reverse total shoulder arthroplasty: A randomized controlled trial Journal of shoulder and elbow surgery. 2023; doi: 101016/jjse202303023.

Schmidt D, Fritsch J, Feil K, Weyland S, Rittmann L-M & Jekauc D. Impact of digital and conventional rehabilitation aftercare on physical and mental health in orthopedic patients in Germany Frontiers in public health. 2024; doi: 103389/fpubh20241344063.

Schwartz I, Safran O, Karniel N, Abel M, Berko A, Seyres M, Tsoar T & Portnoy S. Positive Effect of Manipulated Virtual Kinematic Intervention in Individuals with Traumatic Stiff Shoulder: A Pilot Study Journal of clinical medicine. 2022; 103390/jcm11133919.

Semjonova G, Vetra J, Cauce V, Oks A, Katashev A & Eizentals P. Improving the Recovery of Patients with Subacromial Pain Syndrome with the DAid Smart Textile Shirt Sensors. 2020; doi: 103390/s20185277.

Seven B, Gokkurt A, Koc M, Kupeli B & Oskay D. Investigation of the barriers to and functional outcomes of telerehabilitation in patients with hand injury Journal of Hand Therapy. 2024; doi: 101016/jjht202310003.

Sharma R, Saxena S & Ranjan R. Short term effects of virtual reality exergaming in frozen shoulder patients: a pilot study. Biochemical and Cellular Archives. 2024; doi: 1051470/bca20242422775.

Suero-Pineda A, Oliva-Pascual-Vaca Á, Durán MR-P, Sánchez-Laulhé PR, García-Frasquet MÁ & Blanquero J. Effectiveness of a telerehabilitation tablet app in combination with face-to-face physiotherapy for people with wrist, hand or finger injuries: A pragmatic multicentre clinical trial Journal of Telemedicine and Telecare. 2025; doi: 101177/1357633X231172245.

Sveistrup H, McComas J, Thornton M, Marshall S, Finestone H, McCormick A, Babulic K & Mayhew A. Experimental studies of virtual reality-delivered compared to conventional exercise programs for rehabilitation Cyberpsychology & behavior : the impact of the Internet, multimedia and virtual reality on behavior and society. 2003; doi: 101089/109493103322011524.

Svingen J, Rosengren J, Turesson C & Arner M. A smartphone application to facilitate adherence to home-based exercise after flexor tendon repair: A randomised controlled trial Clinical Rehabilitation. 2021; doi: 101177/0269215520962287.

Then J W, Shivdas S, Tunku Ahmad Yahaya TS, Ab Razak NI & Choo PT. Gamification in rehabilitation of metacarpal fracture using cost-effective end-user device: A randomized controlled trial Journal of hand therapy : official journal of the American Society of Hand Therapists. 2020; doi: 101016/jjht202003029.

Tokgoz P, Wahnert D, Elsner A, Schack T, Cienfuegos Tellez MA, Conrad J, Vordemvenne T & Dockweiler C. Virtual Reality for Upper Extremity Rehabilitation-A Prospective Pilot Study Healthcare 2023; doi: 103390/healthcare11101498.

Tsvyakh AI, Hospodarskyy AY, Kopytchak IR, Marchenkova NO, Kostjuk VP, Gdanskyi SM, Petriuk BV & Babiy MP. Polytrauma Patients Telerehabilitation after Injuries of the Elbow Joint International Journal Dental and Medical Sciences. 2021; 3(5), 312–316

Tsvyakh AI & Hospodarskyy AJ. Telerehabilitation of patients with injuries of the elbow joint of the upper extremities Minerva Medica. 2019; 110(Supplement 1), 12.

Tsvyakh & Hospodarskyy A. Telerehabilitation of patients with injuries of the elbow joint of the upper extremities Annals of Physical and Rehabilitation Medicine. 2018; doi: 101016/jrehab2018051201.

Turkmen E, Analay Akbaba Y & Altun S. Effectiveness of video-based rehabilitation program on pain, functionality, and quality of life in the treatment of rotator cuff tears: A randomized controlled trial Journal of hand therapy: official journal of the American Society of Hand Therapists. 2020; doi: 101016/jjht201908004.

van den Heuvel SG, de Looze MP, Hildebrandt VH & The KH. Effects of software programs stimulating regular breaks and exercises on work-related neck and upper-limb disorders Scandinavian journal of work, environment & health. 2003; doi: 105271/sjweh712.

van Eck C, Toor A, Banffy MB & Gambardella RA. Web-Based Education Prior to Outpatient Orthopaedic Surgery Enhances Patient Satisfaction Scores: A Prospective, Randomized Controlled Study Arthroscopy - Journal of Arthroscopic and Related Surgery. 2018; doi: 101016/jarthro201810080.

Walter MM, Sirard P, Nero H, Horder H, Dahlberg LE, Tveter AT, Kjeken I & Kiadaliri A. Digitally delivered education and exercises for patients with hand osteoarthritis-An observational study Musculoskeletal care. 2023; doi: 101002/msc1796.

Wiederhold BK & Wiederhold MD. Evaluation of virtual reality therapy in augmenting the physical and cognitive rehabilitation of war veterans International Journal on Disability and Human Development. 2006; 5(3), 211EP – 215.

Xiao X, Huang J, Chen Z, Xia X, Wang S & Yang Z. Effects of computer-assisted wrist/hand training on the improvement of hand function in traumatic hand injuries International Journal of Clinical and Experimental Medicine. 2018; 11(2), 1208EP – 1216.
