# Supplementary material for: The effectiveness of telerehabilitation in upper limb musculoskeletal disorders: a systematic review
Source: BMC Musculoskelet Disord. 2026 May 28;27:462. doi: 10.1186/s12891-026-10008-7 (PMC13220470; doi:10.1186/s12891-026-10008-7)
Supplement: Supplementary file 4 — Additional file 4: Risk of Bias Assessment: RoB 2 assessment of individual results on domain level, ROBINS-I assessments for included NRSIs. [file 12891_2026_10008_MOESM4_ESM.docx]

**Risk of Bias Assessment**

Table 1: ROBINS-I V2 assessment of NRSI studies

| **study** | **Risk of Bias judgement (derived from ROBINS-I V2 questions B1 to B3)** | **Overall judgement ROBINS-I V2** |
| --- | --- | --- |
| **Suero-Pineda et al., 2023** | Allocation was based on access to tablet device;  No information on SES, no adjustment for confounding | Critical risk of bias |
| **Palm et al., 2023** | Allocation was based on patients’ preferences for telerehabilitation;  Groups differed in age, no information on SES, no adjustment for confounding | Critical risk of bias |
| **Chen et al., 2020** | Allocation was based on patients’ familiarity with digital media;  Groups probably differed in SES (small sample size), and in baseline measures of outcome | Critical risk of bias |
| **Mayer et al., 2021** | Allocation was matched for age and fracture type;  Nevertheless, there was a considerable difference between groups in age, no information on SES, confounding not adequately controlled | Critical risk of bias |

ROBINS-I V2: The Risk of Bias in Non-randomized Studies – of Interventions, Version 2, NRSI: non randomized studies of interventions, SES: socioeconomic status,

Table 2: RoB 2 assessment for outcome domain ADL

| **Studie** | **D1** | **D2** | **D3** | **D4** | **D5** | **Overall** |
| --- | --- | --- | --- | --- | --- | --- |
| **Blanquero 2020, QuickDASH** | Low | Low | High | High | Low | High |
| **Chen 2017, SST** | High | Some concerns | Some concerns | Some concerns | Some concerns | High |
| **Martinez-Rico 2018, DASH** | Low | Low | Low | High | Some concerns | High |
| **Barrett 2024, PROMIS UE** | Low | Low | High | Some concerns | Low | High |
| **Blanquero 2019, QuickDASH** | Low | Low | Low | Some concerns | Low | Some concerns |
| **Blasco 2024, QuickDASH** | Some concerns | Low | Low | Some concerns | Some concerns | Some concerns |
| **Choi 2024 QuickDASH** | Some concerns | High | High | High | Some concerns | High |
| **Correia 2022 QuickDASH** | Some concerns | Low | Some concerns | Some concerns | Low | Some concerns |
| **Correia 2022 CMS** | Some concerns | Low | Some concerns | Some concerns | Low | Some concerns |
| **Coughlin 2021, DASH** | Low | Low | Some concerns | Some concerns | Low | Some concerns |
| **Lara 2022, QuickDASH** | Low | Low | Low | Some concerns | Some concerns | Some concerns |
| **Marley 2022 DASH** | Some concerns | Low | Low | Some concerns | Low | Some concerns |
| **Meijer 2024, PRWE** | Low | Low | Some concerns | Some concerns | Low | Some concerns |
| **Pak 2023, QuickDASH** | Low | Low | Low | Some concerns | Low | Some concerns |
| **Pastora-Bernal 2018, CMS** | Low | Low | Low | Some concerns | Some concerns | Some concerns |
| **Roddey 2002, SPADI** | Some concerns | Low | High | Some concerns | Some concerns | High |
| **Sánchez-Laulhé 2023, DASH** | Low | Low | High | High | Low | High |
| **Shim 2023, DASH** | Low | Low | Some concerns | High | Low | High |
| **Tousignant 2020, CMS** | Some concerns | Low | Low | Some concerns | Low | Some concerns |
| **Tousignant 2020, DASH** | Some concerns | Low | Low | Some concerns | Low | Some concerns |
| **Vasavada 2024, ASES** | Some concerns | High | High | High | Low | High |

Rob2: revised Cochrane risk-of-bias tool for randomized trials, ADL: activities of daily living, DASH: disabilities of the arm, shoulder and hand questionnaire, PRWE: patient-rated wrist evaluation, CMS: Constant-Murley score, ASES score: American shoulder and elbow surgeons score, SST: simple shoulder test, PROMIS UE: patient reported outcomes measurement information system upper extremity, SPADI: shoulder pain and disability index, D1: bias arising from the randomization process, D2: bias due to deviations from intended interventions, D3: bias due to missing outcome data, D4: bias in measurement of the outcome, D5: bias in selection of the reported result

Table 3: RoB 2 assessment for outcome domain pain

| **Studie** | **D1** | **D2** | **D3** | **D4** | **D5** | **Overall** |
| --- | --- | --- | --- | --- | --- | --- |
| **Blanquero 2020, VAS** | Low | Low | High | High | Low | High |
| **Chen 2017, VAS** | High | Some concerns | Some concerns | Some concerns | Some concerns | High |
| **Choi 2019, VAS** | Low | Low | Low | High | Low | High |
| **Martinez-Rico 2018, VAS** | Low | Low | Low | High | Some concerns | High |
| **Blanquero 2019, VAS** | Low | Low | Low | Some concerns | Low | Some concerns |
| **Blasco 2024, NRS** | Some concerns | Low | Low | Some concerns | Some concerns | Some concerns |
| **Choi 2024, VAS** | Some concerns | High | High | High | Some concerns | High |
| **Correia 2022, CMS pain** | Some concerns | Low | Some concerns | Some concerns | Low | Some concerns |
| **Lara 2022, VAS** | Low | Low | Low | Some concerns | Some concerns | Some concerns |
| **Meijer 2024, NRS** | Low | Low | Some concerns | Some concerns | Low | Some concerns |
| **Pastora-Bernal 2018, CMS pain** | Low | Low | Low | Some concerns | Some concerns | Some concerns |
| **Sánchez-Laulhé 2023, NRS** | Low | Low | High | High | Low | High |
| **Shim 2023, NRS** | Low | Low | Some concerns | High | Low | High |
| **Tousignant 2020, CMS pain** | Some concerns | Low | Low | Some concerns | Low | Some concerns |
| **Pak 2023, NRS** | Low | Low | Low | Some concerns | Low | Some concerns |

Rob2: revised Cochrane risk-of-bias tool for randomized trials, VAS: visual analogue scale, NRS: numeric rating scale, CMS: Constant-Murley score, D1: bias arising from the randomization process, D2: bias due to deviations from intended interventions, D3: bias due to missing outcome data, D4: bias in measurement of the outcome, D5: bias in selection of the reported result

Table 4: RoB 2 assessment for outcome domain HrQol

| **Studie** | **D1** | **D2** | **D3** | **D4** | **D5** | **Overall** |
| --- | --- | --- | --- | --- | --- | --- |
| **Blasco 2024, EQ-5D-5L** | Some concerns | Low | Low | Some concerns | Some concerns | Some concerns |
| **Lara 2022, VR-12 physical** | Low | Low | Low | Some concerns | Some concerns | Some concerns |
| **Marley 2022, EQ-VAS** | Some concerns | Low | Low | Some concerns | Low | Some concerns |
| **Shim 2023, EQ-5D-5L** | Low | Low | Some concerns | High | Low | High |

Rob2: revised Cochrane risk-of-bias tool for randomized trials, HrQol: health-related quality of life, VR-12: Veterans RAND 12-item health survey, EQ‑5D‑5L: EuroQol five dimensions five levels measurement, EQ-VAS: EuroQol visual analogue scale
