# Supplementary material for: The effectiveness of telerehabilitation in upper limb musculoskeletal disorders: a systematic review
Source: BMC Musculoskelet Disord. 2026 May 28;27:462. doi: 10.1186/s12891-026-10008-7 (PMC13220470; doi:10.1186/s12891-026-10008-7)
Supplement: Supplementary file 5 — Additional file 5: Main characteristics and results from included RCTs: Characteristics and results from RCTs for time point directly after intervention. [file 12891_2026_10008_MOESM5_ESM.docx]

**Main characteristics of included RCTs**

**Telerehabilitation versus standard care (subgroup standard care = in-person care)**

| **Study**  **(Country)** | **Population,**  **age mean (sd),**  **% female/male,**  **N: total (IG/CG),**  **setting** | **Intervention:**  **description (de), dose (do), duration (du), timing (t)** | **Control intervention: De, duration, timing, dose** | **Time point from baseline** | **Instrument: name, scale, direction** | **Point estimates for groups mean (sd)*** | **Standardized effect measure**  **SMD [95% CI],**  **negative values favor telerehab^§^** |
| --- | --- | --- | --- | --- | --- | --- | --- |
| **Barrett et al., 2024**  **(USA)** | adults scheduled for primary thumb CMC arthroplasty with digital access, native speakers, exclusion of concomitant procedures  Age: 61.0 (9.5)  % female/male: 86/14  N: 67 (31/36)  Single centre, urban clinic | De: video-based instruction to home exercise protocol  Do: 3 videos (4 minutes each), instructed exercise dosage same as CG  Du: 8 weeks  T: 4 weeks post op | De: in-person instruction to home exercise protocol  Do: 7 in-person sessions with hand therapist once weekly, 30 minutes per session, instructed exercise dosage same as IG  Du: 8 weeks  T: 4 weeks post op | 12 weeks | **ADL:**  PROMIS UE  (0-100), higher is better | **ADL:**  IG: 40.9 (10.5)  CG: 39.8 (7.5) | -0.12 [-0.63 to 0.39] |
| **Correia et al., 2022**  **(Portugal)** | adults with repair surgery after rotator cuff tear, complex tears and limiting comorbidities excluded  Age: 60.71 (6.9)  % female/male: 78/22  N: 50 (27/23)  Single centre, urban clinic | De: guided exercise program using tablet app in combination with motion sensors providing real-time audio and video biofeedback during exercise sessions. Evaluation and weekly remote adjustment by the assigned therapist + home-based one-to-one physical therapy sessions.  Do: daily exercise with SWORD app (15-30 minutes) for 5 times/week, + 13 sessions in-person, 60 minutes/session  Du: 9 weeks  T: 3 weeks po | De: home-based rehabilitation provided by a physical therapist. Instruction to unsupervised exercising twice weekly.  Do: 30 sessions in-person, 3 times/week, 60 minutes/session + unsupervised exercising for at least 2 days/week  Du: 9 weeks T: 3 weeks po | 12 weeks | **Pain:**  CMS subscale pain (0-15), higher is better  **ADL:**  QuickDASH (0-100), lower is better  CMS total (0-100), higher is better | **Pain: median (SD)**  IG: 10 (10)  CG: 10 (10)  **ADL:**  QuickDASH:  IG: 19.95 (19.47)  CG: 17.79 (15.34)  CMS:  IG: 65.51 (14.83)  CG: 67.72 (11.57) | **pain: median difference [95 % CI]**  0 [-5 to 5]  **ADL:**  QuickDASH:  0.12 [-0.56 to 0.80]  CMS total:  0.16 [-0.51 to 0.84] |
| **Coughlin et al., 2021**  **(United Kingdom)** | adults with nonoperatively treated distal radius fractures, exclusion if unable to access video  Age: 49  % female/male: 68/32  N: 80 (40/40)  Single centre, urban clinic | De: instruction for exercise through videos  Do: 4 instruction videos, self-guided exercising for 6 weeks  Du: 6 weeks  T: five to seven weeks post- injury | De: face-to face instruction to self-guided exercising  Do: In-person sessions with instruction to exercises  Du: 6 weeks  T: five to seven weeks post- injury | 6 weeks | **ADL:**  DASH (0-100), lower is better | **ADL:**  Change in DASH:  IG: 12 (14)  CG: 13 (13) | **ADL:**  Change in DASH:  0.07 [-0.41 to 0.55] |
| **Lara et al, 2022**  **(USA)** | adults with operative treatment after distal radius fracture, fluent in English and compatible device  Age median (IQR):  IG: 54 (46-63)  CG: 58 (46-67)  % female/male: 63/37  N: 51 (22/29)  Single centre, urban clinic | De: video-based Instruction to self-guided exercising  Do: Instruction to daily exercising  Du: 10 weeks  T: 2 weeks after surgery | De: in-person instruction for self-guided exercising  Do: 2 sessions per week for 45 minutes, instruction for exercising same protocol and dosage as intervention group  Du: 10 weeks  T: 2 weeks after surgery | 12 weeks | **Pain:**  VAS (0-10), lower is better  **ADL:**  QuickDASH  (0-100), lower is better  **HrQol:**  VR 12 physical (0-100), higher is better | **Pain: mean (SE)**  IG: 2.3 (0.5)  CG: 2.3 (0.4)  **ADL: mean (SE)**  IG: 19.2 (5.2)  CG: 29.3 (4.8)  **HrQol: mean (SE)**  IG: 45.3 (2.5)  CG: 42.2 (2.3) | **Pain:**   1. [-0.57 to 0.57]   **ADL:**  -0.40 [-0.96 to 0.16]  **HrQol:**  -0.26 [-0.83 to 0.31] |
| **Pastora-Bernal et al., 2018a & Pastora-Bernal et al., 2018b**  **(Spain)** | Adults with operative treatment after subacromial impingement syndrome, technical device with internet access and email usage, exclusion for inability to use technological tools  age: 52.22  % female/male: 44.4/55.6  N: 18 (8/10)  Single centre, clinical rehabilitation service | De: customized (by physiotherapist) exercises program through web application, complemented by videoconference with therapist  Do: Digitally supported exercising 5 days/week, videoconferencing weekly for 30 min  Du: 12 weeks  T: Immediately po | De: face-to-face physical therapy (manual therapy, home exercise programs and other physiotherapy techniques)  Do: 5 sessions/week  Du: 12 weeks  T: Immediately po | 12 weeks | **pain:**  CMS subscale pain (0-15), higher is better  **ADL:**  CMS total (0-100), higher is better | **pain: mean (SE)**  IG: 11.38 (0.46)  CG: 10.3 (0.61)  **ADL: mean (SE)**  IG: 68.5 (0.86)  CG: 71.9 (2.22) | **pain:**  -0.61 [-1.56 to 0.34]  **ADL:**  0.59 [-0.32 to 1.49] |
| **Tousignant et al., 2020**  **(Canada)** | patients discharged with conservative treatment after proximal humerus fracture, access to high-speed internet, multiple or intraarticular fractures and surgical treatment excluded  Age: 63  % female/male: 93.3/6.7  N (randomized): 31  N (analyzed): 30 (15/15)  Single centre, urban clinic | De: combination of supervised exercise sessions including pain management and questions, via teleconferencing and unsupervised exercise  Do: 30 to 45-min exercise sessions, twice daily, either supervised in videoconference or unsupervised at home  Du: 8 weeks.  T: 2-3 weeks after fracture | De: Combination of supervised exercise sessions in-person and unsupervised exercise; same protocol as IG  Do: 30 to 45-min exercise sessions, twice daily, either supervised in person or unsupervised at home  Du: 8 weeks.  T: 2-3 weeks after fracture | 9 weeks | **pain:**  CMS subscale pain (0-15), higher is better  **ADL:**  CMS total (0-100), higher is better  DASH (0-100), lower is better | **pain:**  IG: 12.3 (2.9)  CG: 11.6 (2.9)  **ADL:**  CMS:  IG: 59.1 (9.3)  CG: 55.0 (17.8)  DASH:  IG: 18.7 (16.0)  CG: 20.2 (18.0) | **pain:**  -0.23 [-0.93 to 0.46]  **ADL:**  CMS:  -0.23 [-0.92 to 0.47]  DASH  -0.09 [-0.78 to 0.61] |
| **Vasavada et al., 2024**  **(USA)** | adults (40-80) undergoing rotator cuff repair, English proficiency  Age: 58  % female/male: 56/44  N: 32 (12/20)  Single centre, urban clinic | De: home exercise program through Internet-based telerehabilitation software, regular video conference appointments with physical therapist.  Do: unclear  Du: 6 months  T: Directly post-surgery | De: in person physical therapy, same protocol as IG  Do: unclear  Du: 6 months  T: Directly post-surgery | 24 weeks | **ADL:**  ASES score  (0-100), higher is better | **ADL:** (only visual report):  improved ASES scores in both groups at 6 months with higher values in intervention group. |  |
| **Pak et al., 2023**  **(USA)** | adults (18-80) with tendon related shoulder pain, severe comorbidities excluded, limited English proficiency excluded  Age: 50.25  % female/male: 52/48  N: 90 (46/44)  Single centre, urban clinic | De: fully remote, tailored program including home exercise, education, Cognitive behavioral therapy via web-app using motion sensors  Do: 3 units/week, 20 min/unit  Du: 8 weeks  T: unclear | De: supervised exercise, manual therapy, education, motivational interviewing and Cognitive behavioral therapy when appropriate  Do: 2 units/week, 30 min/unit  Du: 8 weeks  T: unclear | 8 weeks | **pain:**  NRS average (0-10), lower is better  **ADL:**  QuickDASH (0-100), lower is better | **pain: median (95% CI)**  IG: 2.1 [1.9 to 2.3]  CG: 1.5 [1.4 to 1.7]  **ADL: median (95% CI)**  IG: 15.5 [7.7 to 23.2]  CG: 13.1 [5.8 to 20.3] | **pain: median difference [95% CI]**  (positive values in favor of telerehabilitation):  -0.6 [-0.9 to -0.4]  **ADL: median difference [95% CI]** (positive values in favor of telerehabilitation):  -2.3 [-14.7 to 10.0] |
| **Marley et al., 2022**  **(United Kingdom)** | Adults (18-70) diagnosed with shoulder impingement syndrome and operative treatment, internet access and ability to use sensors, limiting comorbidities excluded  Age: 53.65  % female/male: 59/41  N: 64 (31/33)  Three specialist shoulder clinics | De: software-based exercise program including exergaming, tailored to patient's ability, weekly remote review of progress  Do: weekly remote sessions  Du: 12 weeks  T: 0 days po/ 21-28 days po (rotator cuff tear) | De: weekly physical therapy with assessment for progression and provision of standardized home exercise program.  Do: weekly in-person sessions  Du: 12 weeks  T: 0 days po/ 21-28 days po (rotator cuff tear) | 12 weeks | **ADL:**  DASH (0-100), lower is better  OSS (0-48), higher is better  **HrQol:**  EQ-VAS (0-100), higher is better | **ADL:**  DASH:  IG: 23.7  CG: 17.9  p between: 0.315  OSS:  IG: 35.6  CG: 37.6  p between: 0.462  **HrQol:**  Only p-value provided:  no significant difference between groups at any time point (p= 0.587) | **ADL:**  DASH:  0.23 [-0.27 to 0.72]  OSS:  0.17 [-0.33 to 0.66]  **HrQol:**  - |
| **Choi et al., 2024**  **(Republic of Korea)** | adults (>20 years) with distal radius fracture (operative treatment), able to adhere to intervention, limiting comorbidities excluded  Age: 63  % female/male: 85.7/14.3  N: 36 (21/15)  Single centre, urban clinic | De: home-based exercise with smart glove  Do: participants exercised for 1 h twice a day, at least 5 days a week  Du: 12 weeks  T: immediately post op | De: In-person rehabilitation + home exercise self-directed  Do: instructed to exercise for 1 h twice a day, at least 5 days a week, 6 sessions of in-person rehabilitation  Du: 12 weeks  T: immediately post op | 12 weeks | **pain:**  VAS (0-10), lower is better  **ADL:**  QuickDASH (0-100), lower is better  Mayo Score (0-100), higher is better | **pain:**  IG: 0.86 (0.8)  CG: 1.71 (1.0)  **ADL:**  QuickDASH:  IG: 15.2 (7.9)  CG: 22.4 (10.6)  Mayo Score:  IG: 71.4 (14.8)  CG: 66.4 (10.6) | **pain:**  -0.88 [-1.98 to 0.22]  **ADL:**  QuickDASH:  -0.72 [-1.73 to 0.29]  Mayo Score:  -0.36 [-1.35 to 0.63] |
| **Roddey et al., 2002**  **(USA)** | Adults (35-78) with rotator cuff tear, operative treatment  Age: 58  % female/male: 36/64  N: 108 (54/54)  Single centre, urban clinic | De: instruction to self-guided exercising via three videos  Do: Three videos  Du: 24 weeks  T: Unclear | De: in-person instruction to self-guided exercising  Do: 4 in-person instruction sessions à 15 minutes  Du: 24 weeks  T: Unclear | 24 weeks | **ADL:**  SPADI (0-100), lower is better  Penn score (0-100), higher is better | **ADL:**  SPADI:  IG: 18.1 (16.1)  CG: 15.3 (21.6)  Penn Score:  IG: 79.4 (15.5)  CG: 79.6 (17.3) | **ADL:**  SPADI  0.18 [-0.23 to 0.59]  Penn Score  0.01 [-0.40 to 0.42] |

RCT: randomized controlled trial, SMD: Standardized mean difference, CI: confidence interval, sd: standard deviation, SE: standard error, IG: intervention group, CG: control group, CMC: carpometacarpal, ADL: activities of daily living, HrQol: health-related quality of life, NSAID: non-steroidal anti-inflammatory drug, VAS: visual analogue scale, NRS: numeric rating scale, CMS: Constant-Murley score, DASH: disabilities of the arm, shoulder and hand questionnaire, VR-12: Veterans RAND 12-item health survey, EQ‑5D‑5L: EuroQol five dimensions five levels measurement, OSIS: Oxford shoulder instability score, SST: simple shoulder test, PROMIS UE: patient reported outcomes measurement information system upper extremity, OSS: Oxford shoulder score, EQ-VAS: EuroQol visual analogue scale, ASES score: American shoulder and elbow surgeons score, Mayo: modified Green-O’Brien score, SPADI: shoulder pain and disability index, Penn score: Pennsylvania shoulder score:, PRWE: patient-rated wrist evaluation, AUSCAN: Australian/Canadian osteoarthritis hand index

*data are presented from values of endpoint measures, if reported otherwise, data details are presented, ^§^if no SMD could be derived other effect estimates are presented with details

**Telerehabilitation versus standard care (subgroup standard care = minimal rehabilitation)**

| **Study**  **(Country)** | **Population,**  **age mean (sd),**  **% female/male,**  **N: total (IG/CG),**  **setting** | **Intervention:**  **description (de), dose (do), duration (du), timing (t)** | **Control intervention: De, duration, timing, dose** | **Time point from baseline** | **Instrument: name, scale, direction** | **Point estimates for groups mean (sd)*** | **Standardized effect measure**  **SMD [95% CI],**  **negative values favor telerehab^§^** |
| --- | --- | --- | --- | --- | --- | --- | --- |
| **Blanquero et al., 2019**  **(Spain)** | patients with carpal tunnel syndrome, operative treatment and access to tablet device, severe comorbidities excluded  Age: 50  % female/male: 82/18  N: 50 (25/25)  two urban clinics | De: tailored home exercise program using tablet app, weekly monitoring  Do: one session a day (25 minutes) on at least 5 days a week  Du: 4 weeks  T: Around 10 days po | De: paper-based instruction to self-guided home exercise, monitoring through weekly telephone call  Do: one session a day (25 minutes) on at least 5 days a week  Du: 4 weeks  T: Around 10 days po | 4 weeks | **pain:**  VAS (0-10), lower is better  **ADL:**  QuickDASH  (0-100), lower is better | **pain:**  IG: 4.0 (2.7)  CG: 5.0 (3.2)  **ADL:**  IG: 39 (24)  CG: 53 (24) | **pain:**  -0.33 [-0.89 to 0.23]  **ADL:**  -0.57 [-1.13 to -0.02] |
| **Blasco et al., 2024**  **(Spain)** | patients undergoing RSA, possession of and familiarity with smartphone, comorbidities excluded, Spanish proficiency  Age: 70.4 (3.6)  % female/male: 68/32  N: 31(17/14)  Single centre, urban clinic | De: rehabilitation program assisted with a chatbot: informative messages, recording of exercise sessions, record of symptoms, weekly report on progress and compliance  Do: one instruction session; instruction to perform exercises in 48-50 sessions  Du: 12 weeks  T: one week po | De: same rehabilitation protocol brochure based and one supervision session  Do: one instruction session and one additional supervision session; instruction to perform exercises in 48-50 sessions  Du: 12 weeks  T: one week po | 12 weeks | **pain:**  NRS (0-10), lower is better  **ADL:**  QuickDASH  (0-100), lower is better  CMS (0-100), higher is better  **HrQol:**  EQ5D5L (0-1), higher is better | **pain:**  IG: 1.9 (1.4)  CG: 2.2 (2.0)  **ADL:**  QuickDASH:  IG: 23.8 (9)  CG: 38.6 (12.8)  CMS:  IG: 63.5 (12.6)  CG: 58.4 (15.3)  **HrQol:**  IG: 0.7 (0.2)  CG: 0.7 (0.2) | **pain:**  -0.17 [-0.88 to 0.54]  **ADL:**  QuickDASH:  -1.33 [-2.09 to -0.57]  CMS:  -0.36 [-1.05 to 0.34]  **HrQol:**  EQ5D5L:   1. [-0.71 to 0.71] |
| **Meijer et al., 2024**  **(Netherlands)** | Adults (18 or older) with distal radius fracture, operative or conservative treatment, who own compatible smartphone or tablet, language barriers to questionnaires  Age mean (IQR): 52 (42-65)  % female/male: 82.8/17.2  N: 93 (47/46)  three regional hospitals, one academic hospital | De: smartphone or tablet-based exergaming. Upon request referral to hand-therapist  Do: Exergaming three to five times per day, for 10–15 min.  Du: 6 weeks  T: 3-5 days po or after cast removal | De: home-based unsupervised wrist exercises. Upon request or recommendation by clinician referral to hand-therapist  Do: Instruction to perform the exercises three to five times per day, for 10–15 min.  Du: 6 weeks  T: 3-5 days po or after cast removal | 6 weeks | **pain:**  NRS, (0-10), lower is better  **ADL:**  PRWE (0-100), lower is better | **pain:**  only reported visually: at week 6 values endpoint values for both groups are close to each other, confidence intervals overlapping  **ADL:**  Change in PRWE (higher is better) [95% CI]  IG: 23.9 [18.5 to 29.4]  CG: 24.8 [18.2 to 31.4] | **pain:**  -  **ADL:**  Change in PRWE:  0.16 [-0.24 to 0.57] |
| **Rodríquez-Sánchez-Laulhé et al., 2023**  **(Spain)** | Adults (18+) diagnosed with hand osteoarthritis, own smartphone or tablet with internet access, comorbidities excluded  Age: 63  % female/male: 67.5/32.5  N Participants: 74 (34/40)  N hands: 144 (66/78)  two rural community health centres | De: home exercise with tablet app (diary function, self-management recommendations, exercise instructions, symptom report); monthly follow-up phone calls  Do: 15-20 min 4 times/week  Du: 12 weeks  T: not reported | De: paper-based home exercise program. Introductory face-to-face session to explain the exercise program. Monitoring telephone calls once a month  Do: Instruction to exercise 15-20 min 4 times/week  Du: 12 weeks  T: not reported | 12 weeks | **pain:**  NRS (0-10), lower is better  AUSCAN pain (0-25), lower is better  **ADL:**  QuickDASH  (0-100), lower is better  Change in AUSCAN, lower is better | **pain:**  change in NRS:  IG: -0.7 (2.4)  CG: 0.4 (3.5)  Change in AUSCAN pain:  IG: -1.3 (4.6)  CG: -0.4 (5.1)  **ADL:**  Change in QuickDASH:  IG: -7.9 (16.1)  CG: 1.1 (24.9)  Change in AUSCAN:  IG: -2.9 (9.1)  CG: -0.5 (13.3) | **pain:**  change in NRS:  -0.59 [-0.95 to -0.23]  Change in AUSCAN pain:  -0.18 [-0.54 to 0.17]  **ADL:**  Change in QuickDASH:  -0.42 [-0.78 to -0.06]  Change in AUSCAN:  -0.21 [-0.56 to 0.15] |
| **Shim et al., 2023**  **(Republic of Korea)** | Adults (50+) with rotator cuff tear, operative treatment, limiting comorbidities excluded  Age (sd): 64  % female/male: 61/39  N: 115 (58/57)  Single centre, urban clinic | De: brochure-based exercise for 6 weeks followed by AR-based exercises for 6 weeks with real-time feedback on exercises and session performance, feedback at 6 and 12 weeks by physician.  Do: instruction to perform 3–5 sets of exercises with 10 repetitions of each set per day  Du: 6 weeks  T: Few days po (brochure-based exercises)  6 weeks: AR exercising | De: brochure-based home exercises, exercise diary, condition checked by weekly telephone call.  Do: instruction to perform 3–5 sets of exercises with 10 repetitions of each set per day  Du: 6 weeks  T: Few days po  (brochure-based exercises) | 12 weeks | **pain:**  NRS (0-10), lower is better  **ADL:**  SPADI (0-100), lower is better  DASH (0-100), lower is better  SST, 0-12, higher is better  **HrQol:**  EQ5D5L (0-1), higher is better | **pain:**  IG: 3.56 (1.98)  CG: 3.53 (1.83)  **ADL:**  SPADI:  IG: 26.9 (14.11)  CG: 27.89 (15.98)  DASH:  IG: 21.42 (10.40)  CG: 24.88 (14.44)  SST:  IG: 6.24 (2.63)  CG: 7.8 (3.14)  **HrQol:**  IG: 0.802 (0.053)  CG: 0.769 (0.103) | **pain:**  0.02 [-0.36 to 0.39]  **ADL:**  SPADI:  -0.07 [-0.44 to 0.31]  DASH:  -0.27 [-0.65 to 0.10]  SST:  -0.36 [-0.74 to 0.01]  **HrQol:**  -0.40 [-0.78 to -0.02] |

RCT: randomized controlled trial, SMD: Standardized mean difference, CI: confidence interval, sd: standard deviation, SE: standard error, IG: intervention group, CG: control group, CMC: carpometacarpal, ADL: activities of daily living, HrQol: health-related quality of life, NSAID: non-steroidal anti-inflammatory drug, VAS: visual analogue scale, NRS: numeric rating scale, CMS: Constant-Murley score, DASH: disabilities of the arm, shoulder and hand questionnaire, VR-12: Veterans RAND 12-item health survey, EQ‑5D‑5L: EuroQol five dimensions five levels measurement, OSIS: Oxford shoulder instability score, SST: simple shoulder test, PROMIS UE: patient reported outcomes measurement information system upper extremity, OSS: Oxford shoulder score, EQ-VAS: EuroQol visual analogue scale, ASES score: American shoulder and elbow surgeons score, Mayo: modified Green-O’Brien score, SPADI: shoulder pain and disability index, Penn score: Pennsylvania shoulder score:, PRWE: patient-rated wrist evaluation, AUSCAN: Australian/Canadian osteoarthritis hand index

*data are presented from values of endpoint measures, if reported otherwise, data details are presented, ^§^if no SMD could be derived other effect estimates are presented with details

**Telerehabilitation as add-on to standard care versus standard care without/minimal add-on**

| **Study**  **(Country)** | **Population,**  **age mean (sd),**  **% female/male,**  **N: total (IG/CG),**  **setting** | **Intervention:**  **description (de), dose (do), duration (du), timing (t)** | **Control intervention: De, duration, timing, dose** | **Time point from baseline** | **Instrument: name, scale, direction** | **Point estimates for groups mean (sd)*** | **Standardized effect measure**  **SMD [95% CI],**  **negative values favor telerehab^§^** |
| --- | --- | --- | --- | --- | --- | --- | --- |
| **Blanquero et al., 2020**  **(Spain)** | Adults (18-65) with work-related hand or finger injury, limiting comorbidities excluded  Age: 44  % female/male: 38/62  N: 74 (40/34)  Single centre, urban clinic | De: in-person sessions same in both groups, App-based home exercise program including reports for weekly monitoring with care provider  Do: In person sessions 3 days per week, for 30 to 60 minutes. 20-30 minutes home exercise  Du: 4 weeks  T: Directly after referral to rehabilitation service (cast removal or po clearance) | De: in-person sessions same in both groups, paper-based home exercise program, weekly supervision of HEP during in-person session  Do: In person sessions 3 days per week, for 30 to 60 minutes. 20-30 minutes home exercise  Du: 4 weeks  T: Directly after referral to rehabilitation service (cast removal or po clearance) | 4 weeks | **pain:**  VAS (0-10), lower is better  **ADL:**  QuickDASH  (0-100), lower is better | **pain:**  IG: 2.7 (1.7)  CG: 3.6 (2.0)  **ADL:**  IG: 26 (17)  CG: 38 (23) | **pain:**  -0.47 [-1.08 to 0.14]  **ADL:**  -0.57 [-1.17 to 0.03] |
| **Chen et al., 2017**  **(Taiwan)** | Patients with conservative treatment of frozen shoulder, smart-phone possession, Chinese proficiency  Age: 57.5 (8.5)  % female/male: 61.7/38.3  N: 66 (33/33)  Single centre | De: instruction to perform daily exercises, daily text messaging (reminders, encouragement, education)  Do: Instruction to perform exercises 10 minutes/day, 14 text messages  Du: 2 weeks  T: Unclear | De: instruction to perform daily exercises  Do: Instruction to perform exercises 10 minutes/day  Du: 2 weeks  T: Unclear | 2 weeks | **pain:**  VAS (0-100), lower is better  **ADL:**  SST (0-100), higher is better | **pain:**  IG: 12.38 (16.14)  CG: 13.73 (15.51)  **ADL:**  IG: 73.33 (12.99)  CG: 72.76 (17.88) | **pain:**  -0.08 [-0.59 to 0.42]  **ADL:**  0.04 [-0.46 to 0.54] |
| **Choi et al., 2019**  **(Republic of Korea)** | Adults with frozen shoulder  Age: 57.3  % female/male: 67.9/32.1  N: 84 (42/42)  Single centre, urban clinic | De: NSAIDs + education to self-guided exercise, app-based support of exercises with reminders and feedback  Do: Instruction to perform exercises 2-3 times daily  Du: 12 weeks  T: Directly after presentation at clinic | De: NSAIDs + education to self-guided exercise  Do: Instruction to perform exercises 2-3 times daily  Du: 12 weeks  T: Directly after presentation at clinic | 12 weeks | **pain:**  VAS (0-10), lower is better | **pain:**  IG: 1.8 (2.5)  CG: 2.2 (1.7) | **pain:**  -0.19 [-0.61 to 0.24] |
| **Martinez-Rico et al., 2018**  **(Spain)** | Adults with shoulder instability and Bankart repair  Age: 28  % female/male: 23/77  71 (36/35)  Single centre | De: 3 weeks of out-patient physical therapy with home exercise program + phone-based coaching to self-care  Do: + 3 phone calls/week during first month  Du: 4 weeks  T: 3-4 weeks po (at sling removal) | De: 3 weeks of out-patient physical therapy with home exercise program  Do: Same outpatient rehabilitation as IG  Du: 4 weeks  T: 3-4 weeks po (at sling removal) | 8 weeks (VAS)  16 weeks (DASH) | **pain:**  VAS (0-10), lower is better  **ADL:**  DASH (0-100), lower is better  OSIS (12-60), lower is better | **pain:**  IG: 1.7  CG: 4.5  P between: 0.001  **ADL:**  DASH  IG: 9.0  CG: 25.9  P between: 0.037  OSIS:  IG: 20.4  CG: 26.4  P between: 0.074 | **pain:**  -0.81 [-1.30 to -0.33]  **ADL:**  DASH:  -0.47 [-0.94 to 0.00]  OSIS:  -0.40 [-0.87 to 0.07] |

RCT: randomized controlled trial, SMD: Standardized mean difference, CI: confidence interval, sd: standard deviation, SE: standard error, IG: intervention group, CG: control group, CMC: carpometacarpal, ADL: activities of daily living, HrQol: health-related quality of life, NSAID: non-steroidal anti-inflammatory drug, VAS: visual analogue scale, NRS: numeric rating scale, CMS: Constant-Murley score, DASH: disabilities of the arm, shoulder and hand questionnaire, VR-12: Veterans RAND 12-item health survey, EQ‑5D‑5L: EuroQol five dimensions five levels measurement, OSIS: Oxford shoulder instability score, SST: simple shoulder test, PROMIS UE: patient reported outcomes measurement information system upper extremity, OSS: Oxford shoulder score, EQ-VAS: EuroQol visual analogue scale, ASES score: American shoulder and elbow surgeons score, Mayo: modified Green-O’Brien score, SPADI: shoulder pain and disability index, Penn score: Pennsylvania shoulder score:, PRWE: patient-rated wrist evaluation, AUSCAN: Australian/Canadian osteoarthritis hand index

*data are presented from values of endpoint measures, if reported otherwise, data details are presented, ^§^if no SMD could be derived other effect estimates are presented with details
