# Supplementary material for: The effectiveness of telerehabilitation in upper limb musculoskeletal disorders: a systematic review
Source: BMC Musculoskelet Disord. 2026 May 28;27:462. doi: 10.1186/s12891-026-10008-7 (PMC13220470; doi:10.1186/s12891-026-10008-7)
Supplement: Supplementary file 6 — Additional file 6: Results from meta-analysis: Forest plots of meta-analysis results including subgroup analysis. [file 12891_2026_10008_MOESM6_ESM.docx]

**Results from meta-analysis**

Table of contents

[Pain 2](#_Toc226714915)

[VAS/NRS telerehabilitation versus standard care 2](#_Toc226714916)

[Subgroup analysis: telerehabilitation versus minimal care, overall Risk of Bias 2](#_Toc226714917)

[Subgroup analysis: telerehabilitation versus minimal care, duration of intervention >12 weeks 3](#_Toc226714918)

[Subgroup analysis: telerehabilitation versus minimal care, diagnosis 3](#_Toc226714919)

[VAS/NRS telerehabilitation as add on versus no/minimal 4](#_Toc226714920)

[Sensitivity analysis: telerehabilitation as add-on, excluding Chen 2017 4](#_Toc226714921)

[Subgroup analysis: telerehabilitation as add-on, duration of intervention >12 weeks 5](#_Toc226714922)

[Subgroup analysis: telerehabilitation versus minimal care, diagnosis 5](#_Toc226714923)

[CMS Pain telerehabilitation versus standard care 6](#_Toc226714924)

[ADL 6](#_Toc226714925)

[QuickDASH/DASH telerehabilitation versus standard care 6](#_Toc226714926)

[Subgroup analysis: telerehabilitation versus in-person care, overall Risk of Bias 7](#_Toc226714927)

[Subgroup analysis: telerehabilitation versus in-person care, duration of intervention >12 weeks 7](#_Toc226714928)

[Subgroup analysis: telerehabilitation versus in-person care, diagnosis 8](#_Toc226714929)

[Subgroup analysis: telerehabilitation versus minimal care, overall Risk of Bias 8](#_Toc226714930)

[Subgroup analysis: telerehabilitation versus minimal care, duration of intervention >12 weeks 9](#_Toc226714931)

[Subgroup analysis: telerehabilitation versus minimal care, diagnosis 9](#_Toc226714932)

[Sensitivity analysis: telerehabilitation versus minimal care, without Blasco 10](#_Toc226714933)

[QuickDASH/DASH telerehabilitation as add on versus no add on 10](#_Toc226714934)

[CMS telerehabilitation versus standard care 11](#_Toc226714935)

[Subgroup analysis: telerehabilitation versus in-person care, overall Risk of Bias 11](#_Toc226714936)

[Subgroup analysis: telerehabilitation versus in-person care, Intervention duration 12](#_Toc226714937)

[Subgroup analysis: telerehabilitation versus in-person care, diagnosis 12](#_Toc226714938)

[HrQol: EQ5D5L VAS/NRS telerehabilitation versus standard care 12](#_Toc226714939)

# Pain

## VAS/NRS telerehabilitation versus standard care

Figure 1: forest plot of outcome pain (VAS/NRS), telerehabilitation versus standard care


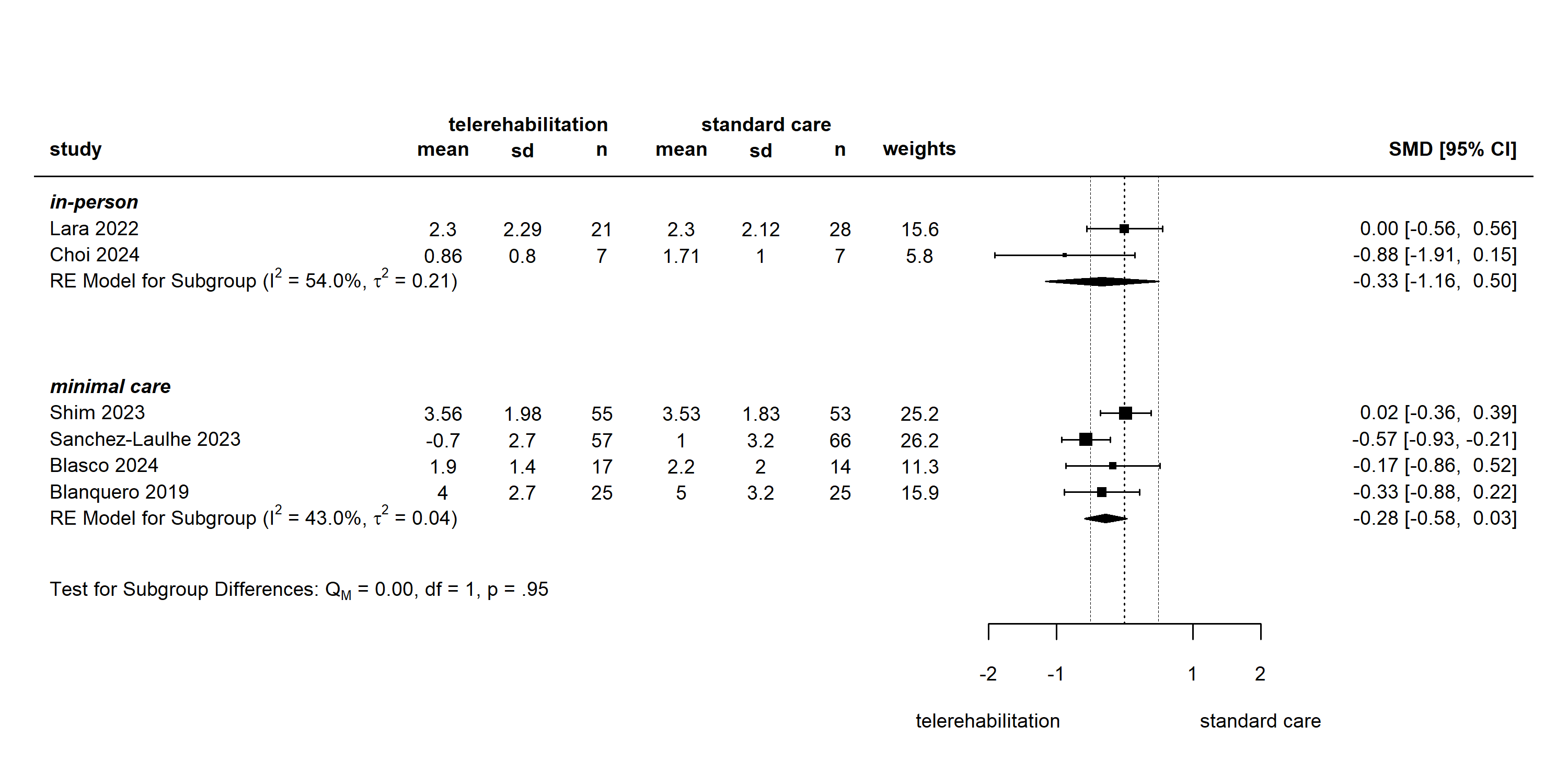


SMD: standardized mean difference, sd: standard deviation, CI: confidence interval, RE: random effects, VAS: visual analogue scale, NRS: numeric rating scale

### Subgroup analysis: telerehabilitation versus minimal care, overall Risk of Bias

Figure 2: forest plot subgroup analysis (VAS/NRS), risk of bias


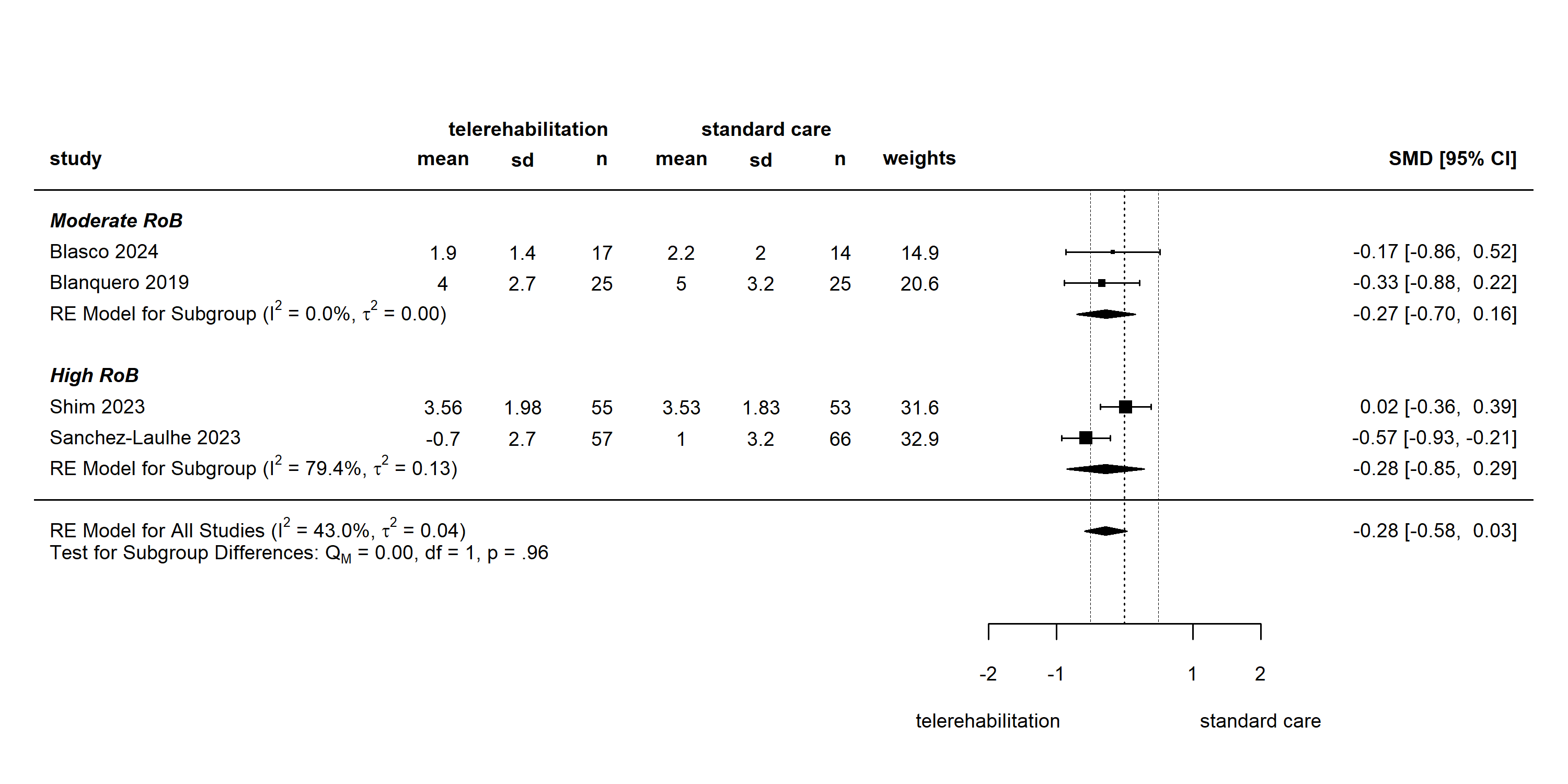


SMD: standardized mean difference, sd: standard deviation, CI: confidence interval, RoB: Risk of Bias, RE: random effects, VAS: visual analogue scale, NRS: numeric rating scale

### Subgroup analysis: telerehabilitation versus minimal care, duration of intervention >12 weeks

Figure 3: forest plot subgroup analysis (VAS/NRS), Intervention duration


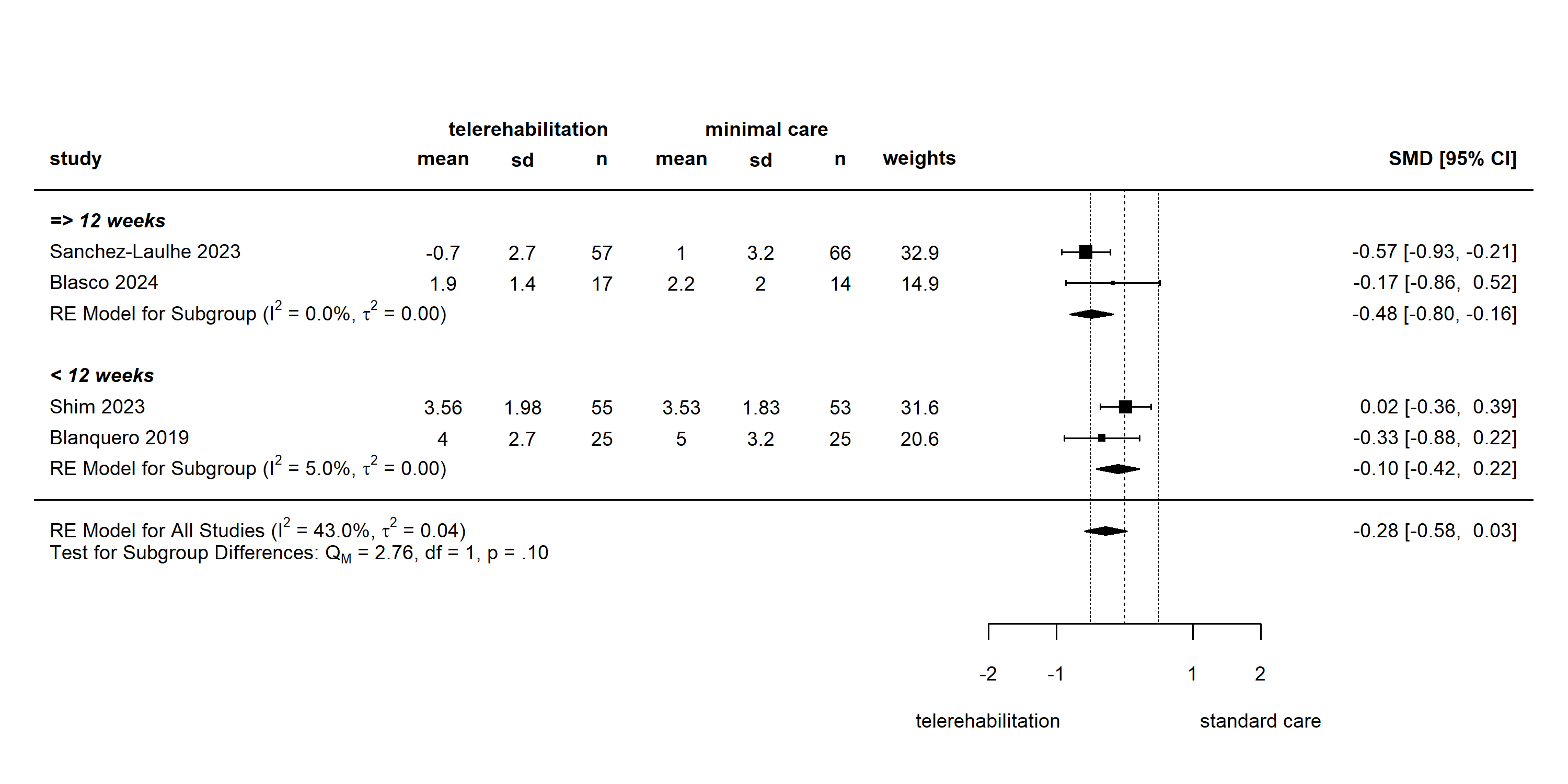


SMD: standardized mean difference, sd: standard deviation, CI: confidence interval, RE: random effects, VAS: visual analogue scale, NRS: numeric rating scale

### Subgroup analysis: telerehabilitation versus minimal care, diagnosis

Figure 4: forest plot subgroup analysis (VAS/NRS), diagnosis


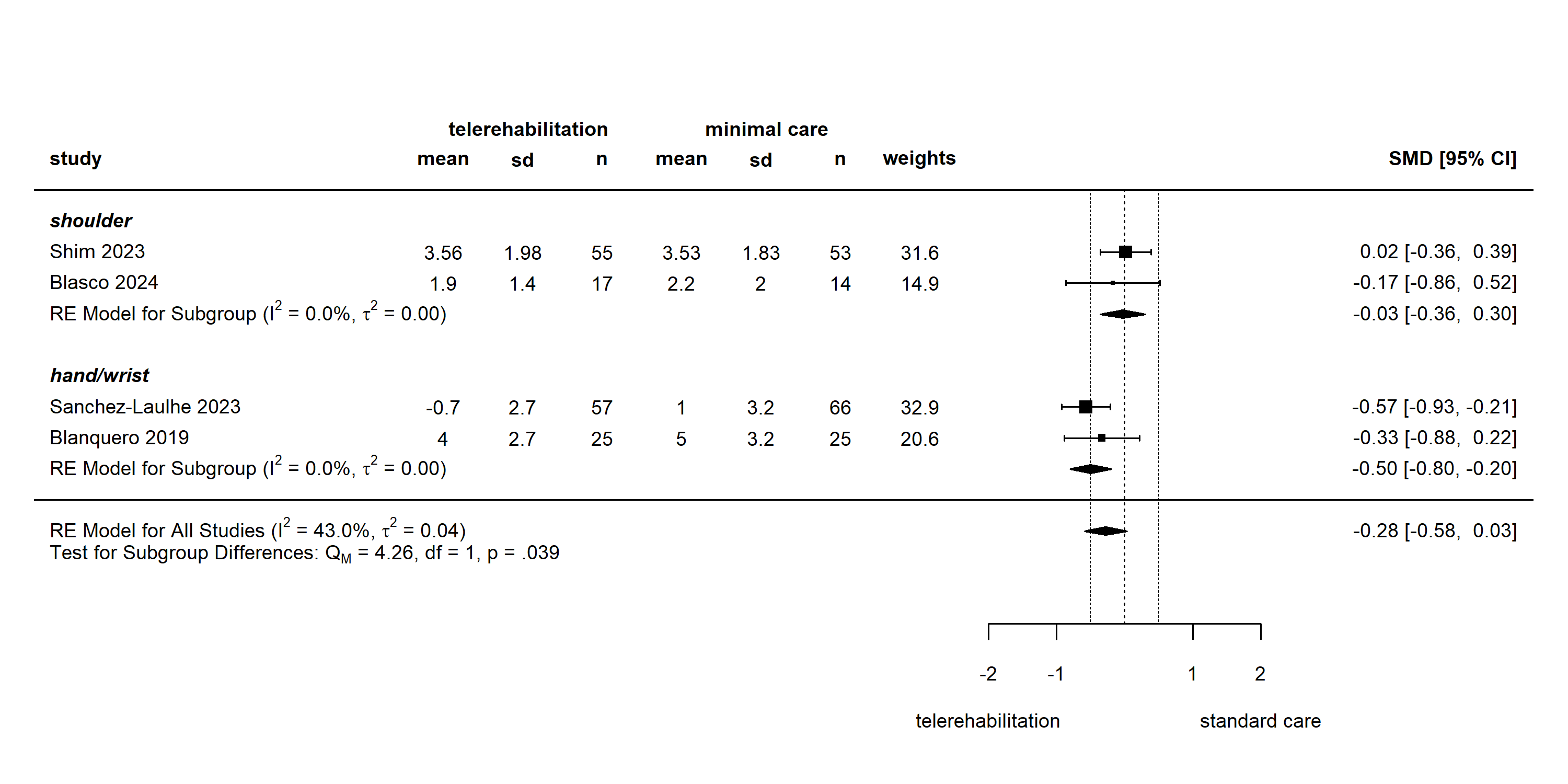


SMD: standardized mean difference, sd: standard deviation, CI: confidence interval, RE: random effects, VAS: visual analogue scale, NRS: numeric rating scale

## VAS/NRS telerehabilitation as add on versus no/minimal

Figure 5: forest plot of outcome pain (VAS/NRS), telerehabilitation as add on versus no/minimal add on


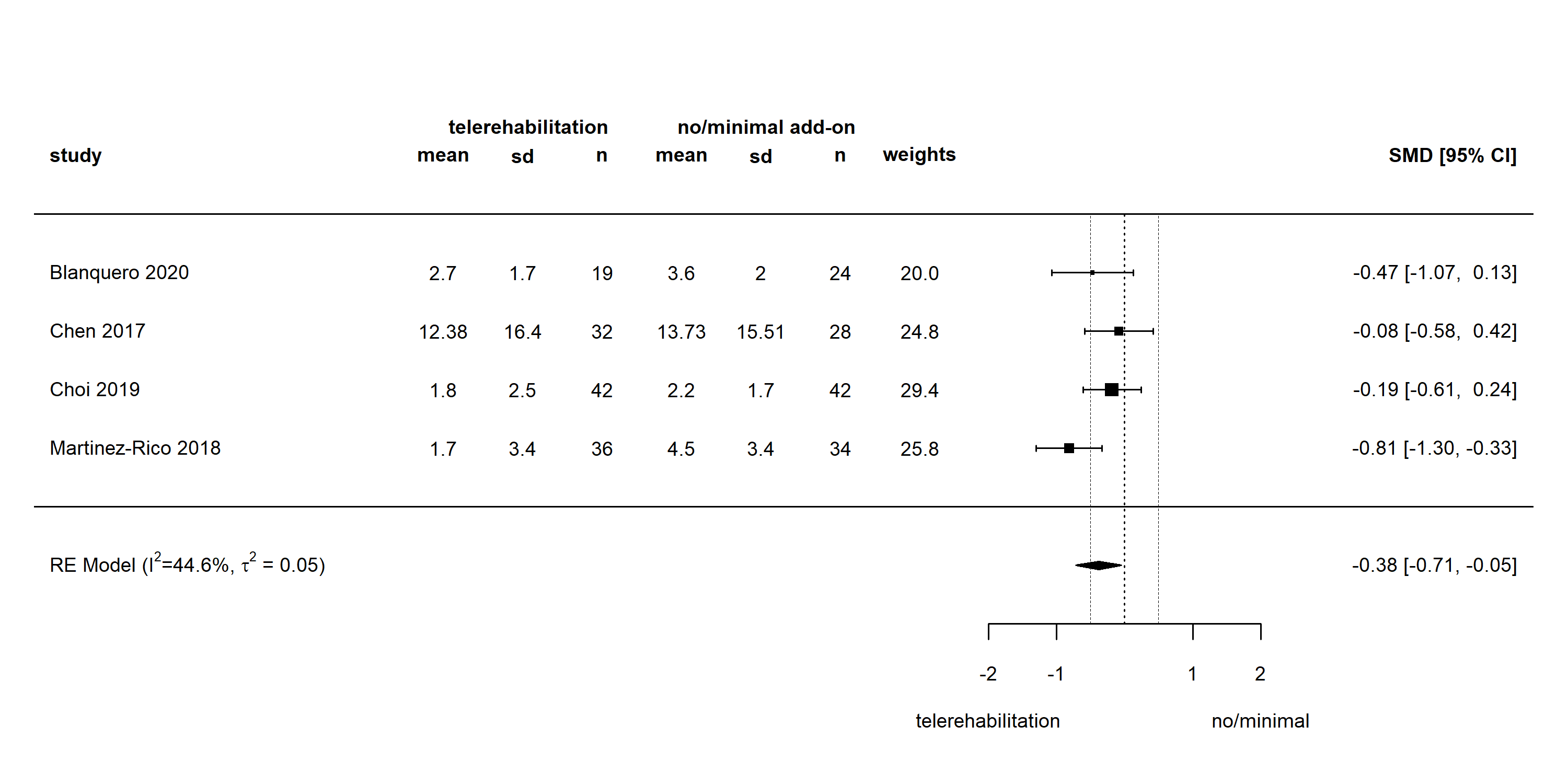


SMD: standardized mean difference, sd: standard deviation, CI: confidence interval, RE: random effects, VAS: visual analogue scale, NRS: numeric rating scale

### Sensitivity analysis: telerehabilitation as add-on, excluding Chen 2017

Figure 6: forest plot subgroup analysis (VAS/NRS), excluding Chen 2017 with risk of bias issues in five domains


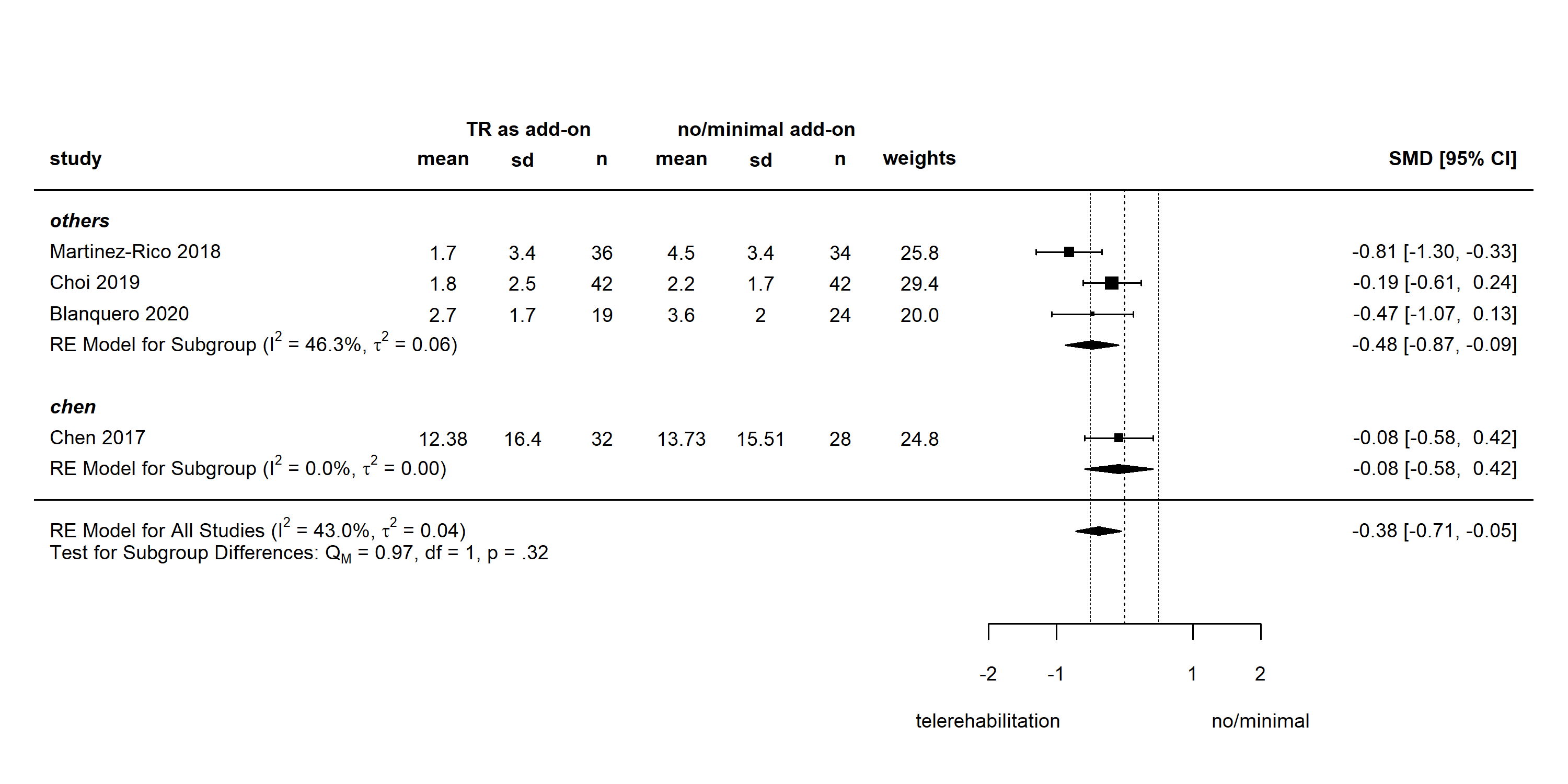


SMD: standardized mean difference, sd: standard deviation, CI: confidence interval, RE: random effects, VAS: visual analogue scale, NRS: numeric rating scale

### Subgroup analysis: telerehabilitation as add-on, duration of intervention >12 weeks

Figure 7: forest plot subgroup analysis (VAS/NRS), intervention duration


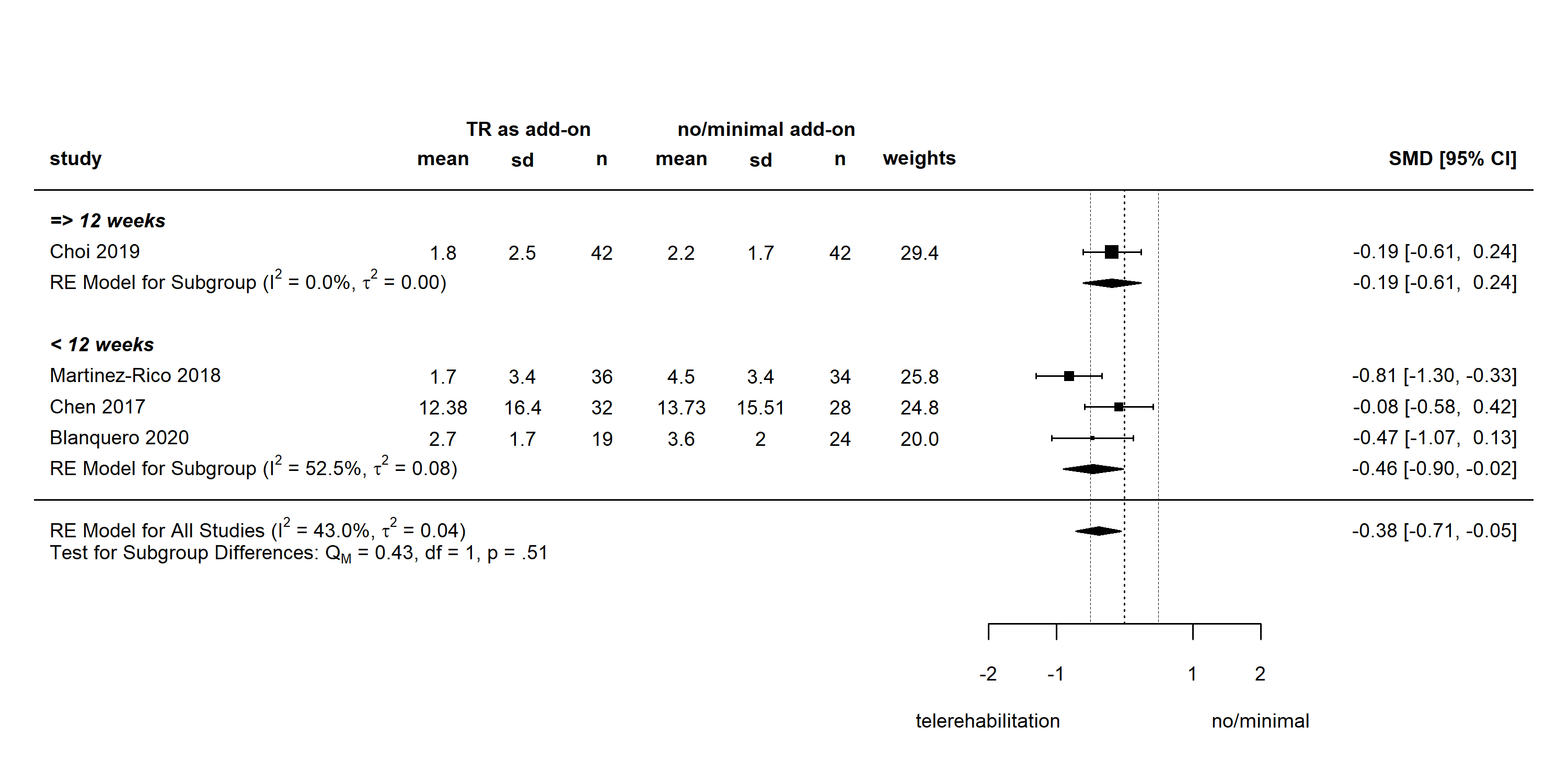


SMD: standardized mean difference, sd: standard deviation, CI: confidence interval, RE: random effects, VAS: visual analogue scale, NRS: numeric rating scale

### Subgroup analysis: telerehabilitation versus minimal care, diagnosis

Figure 8: forest plot subgroup analysis (VAS/NRS), diagnosis


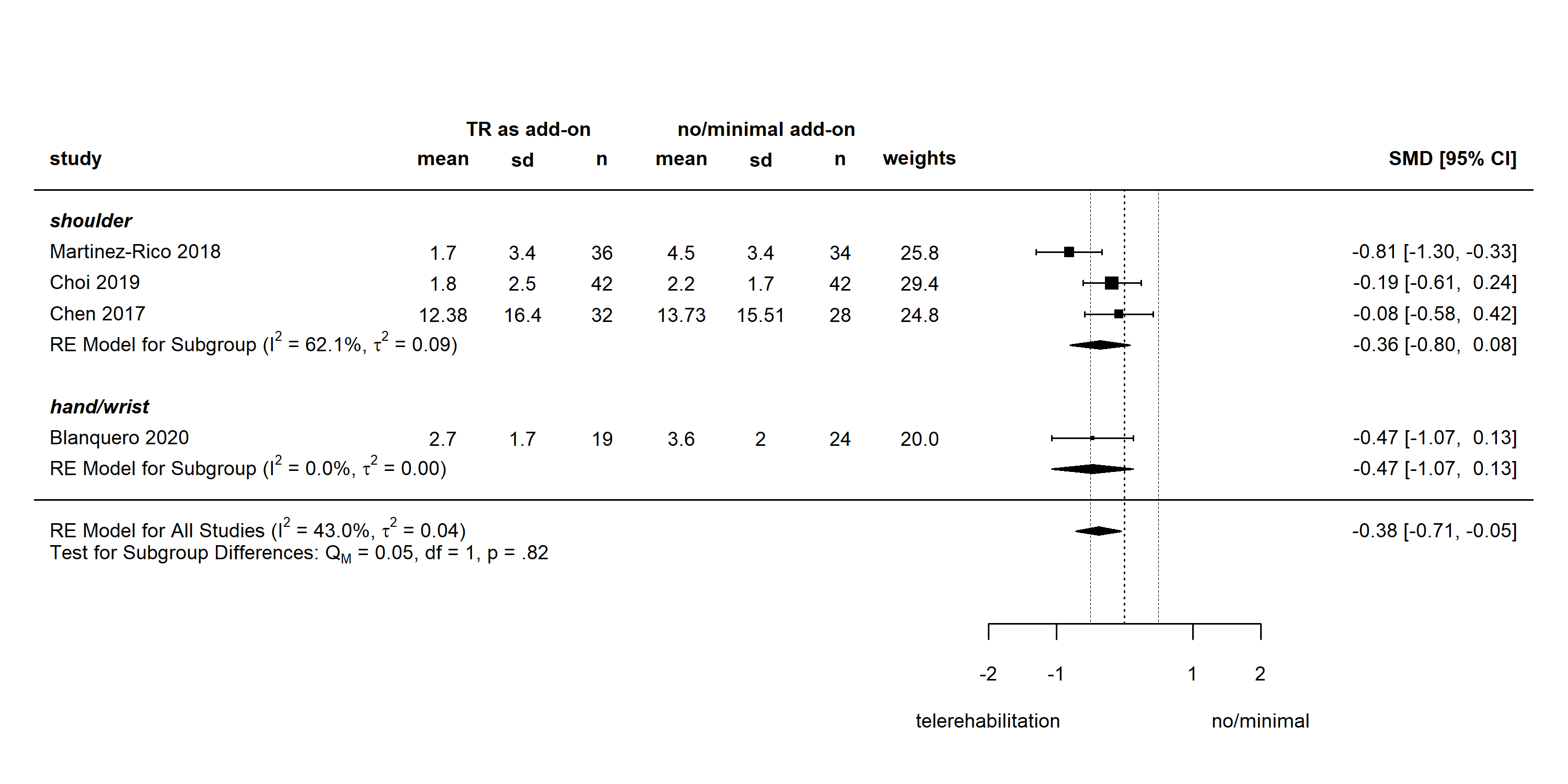


SMD: standardized mean difference, sd: standard deviation, CI: confidence interval, RE: random effects, VAS: visual analogue scale, NRS: numeric rating scale

## CMS Pain telerehabilitation versus standard care

Figure 9: forest plot of outcome pain (CMS pain), telerehabilitation versus in-person standard care


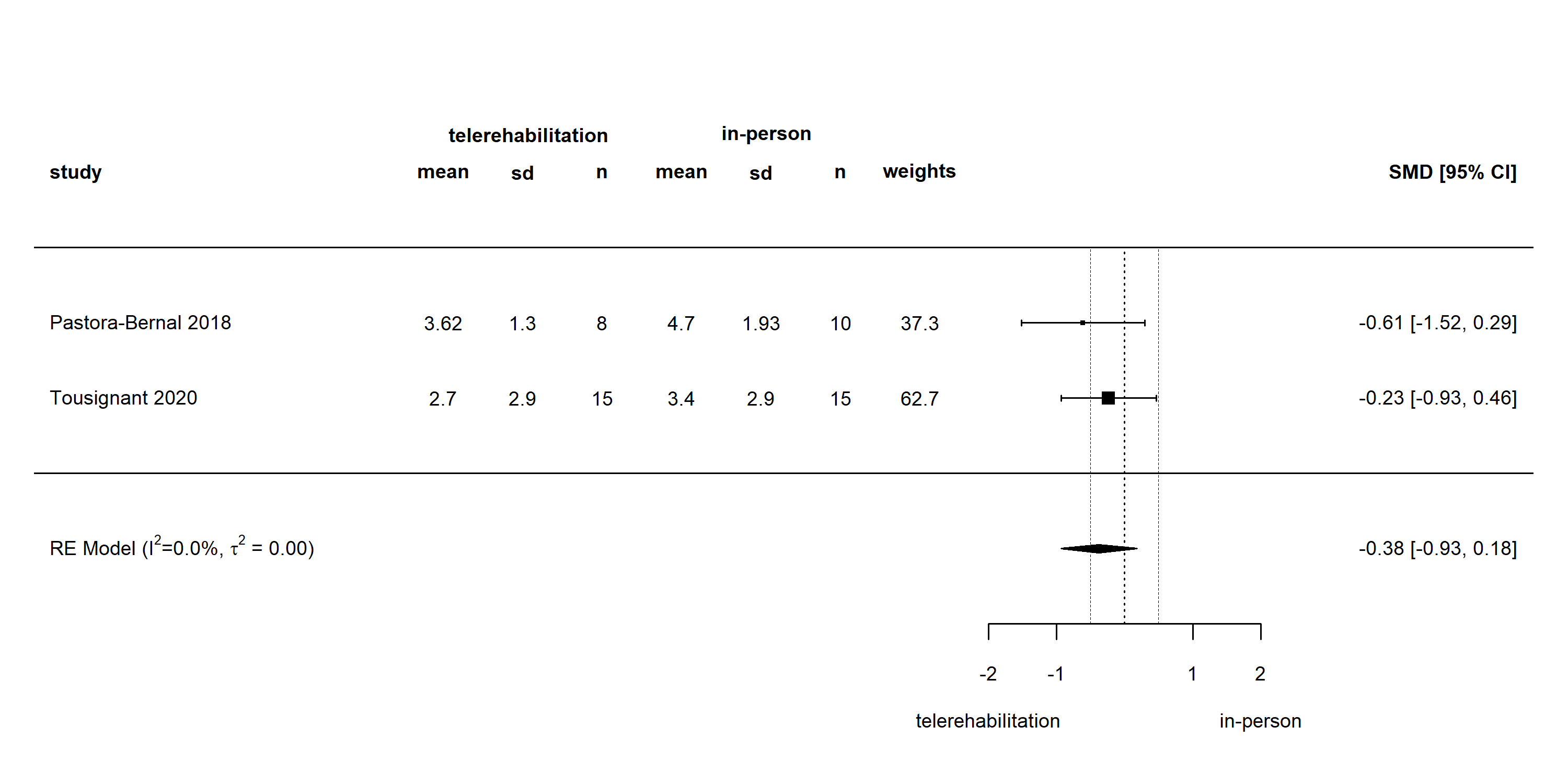


SMD: standardized mean difference, sd: standard deviation, CI: confidence interval, RE: random effects, CMS: Constant-Murley score

# ADL

## QuickDASH/DASH telerehabilitation versus standard care

Figure 10: forest plot of outcome ADL (QuickDASH/DASH), telerehabilitation versus standard care


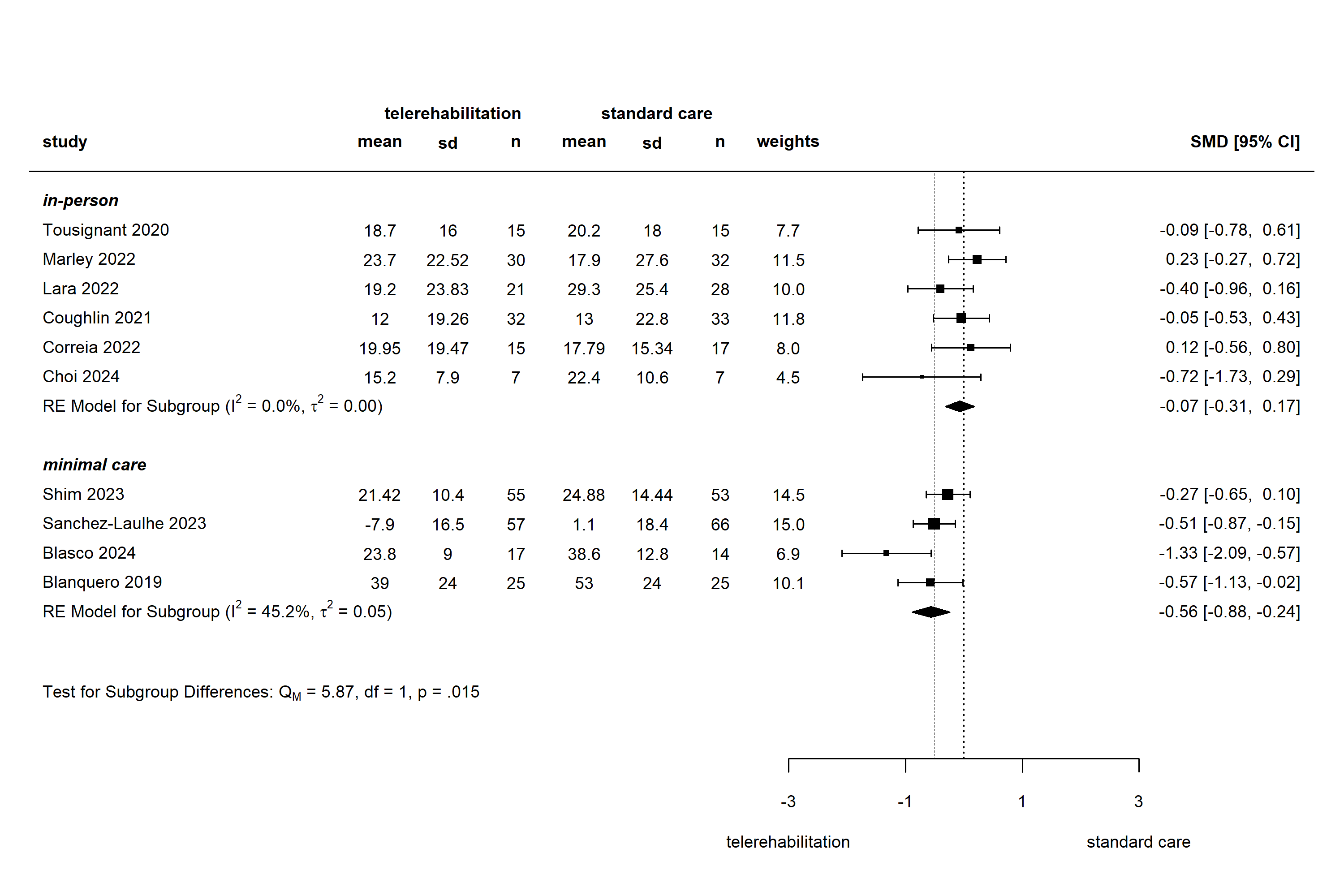


SMD: standardized mean difference, sd: standard deviation, CI: confidence interval, RE: random effects, DASH: disabilities of the arm, shoulder and hand questionnaire

### Subgroup analysis: telerehabilitation versus in-person care, overall Risk of Bias

Figure 11: forest plot subgroup analysis (QuickDASH/DASH), risk of bias


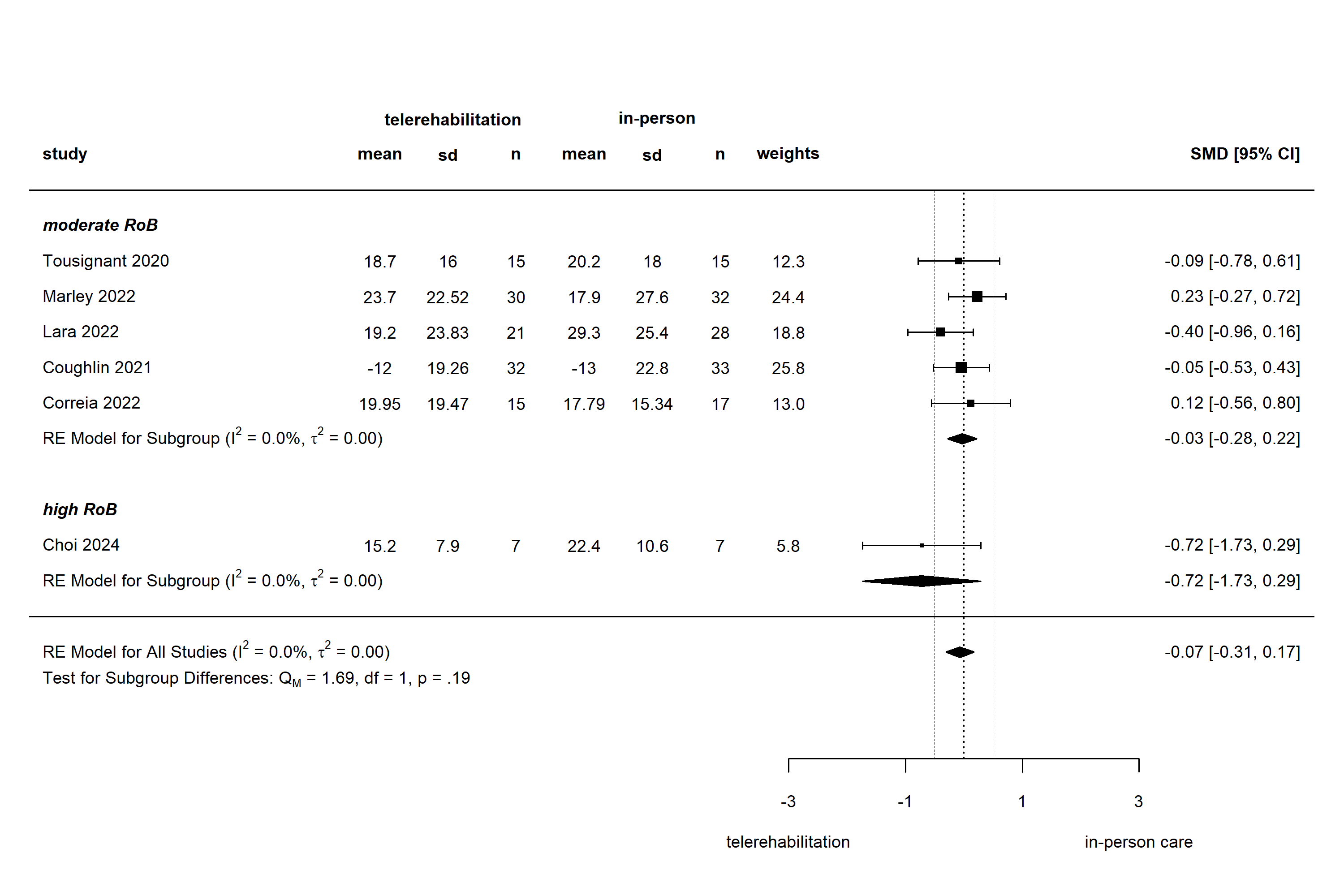


SMD: standardized mean difference, sd: standard deviation, CI: confidence interval, RoB: Risk of Bias, RE: random effects, DASH: disabilities of the arm, shoulder and hand questionnaire

### Subgroup analysis: telerehabilitation versus in-person care, duration of intervention >12 weeks

Figure 12: forest plot subgroup analysis (QuickDASH/DASH), intervention duration


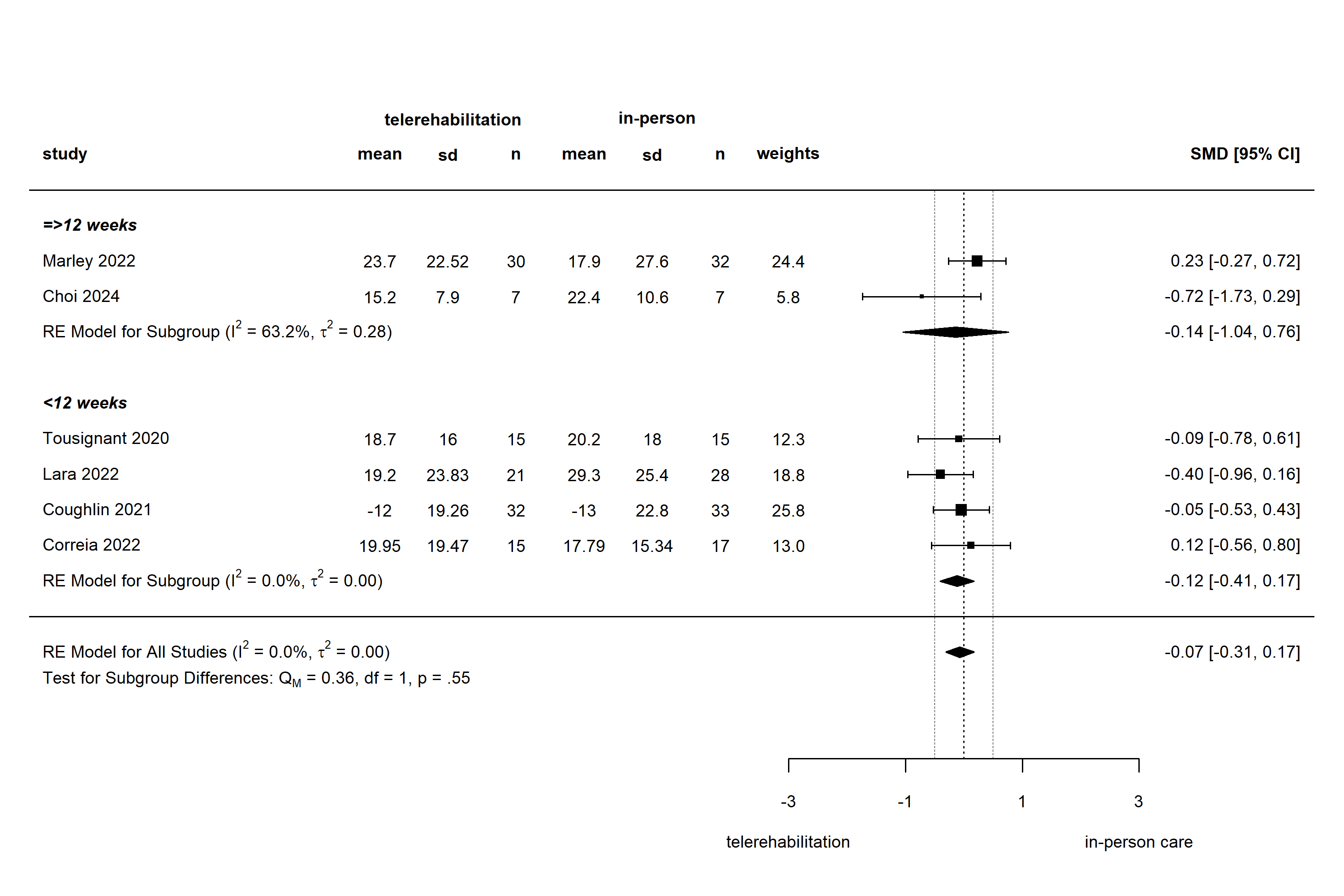


SMD: standardized mean difference, sd: standard deviation, CI: confidence interval, RE: random effects, DASH: disabilities of the arm, shoulder and hand questionnaire

### Subgroup analysis: telerehabilitation versus in-person care, diagnosis

Figure 13: forest plot subgroup analysis (QuickDASH/DASH), diagnosis


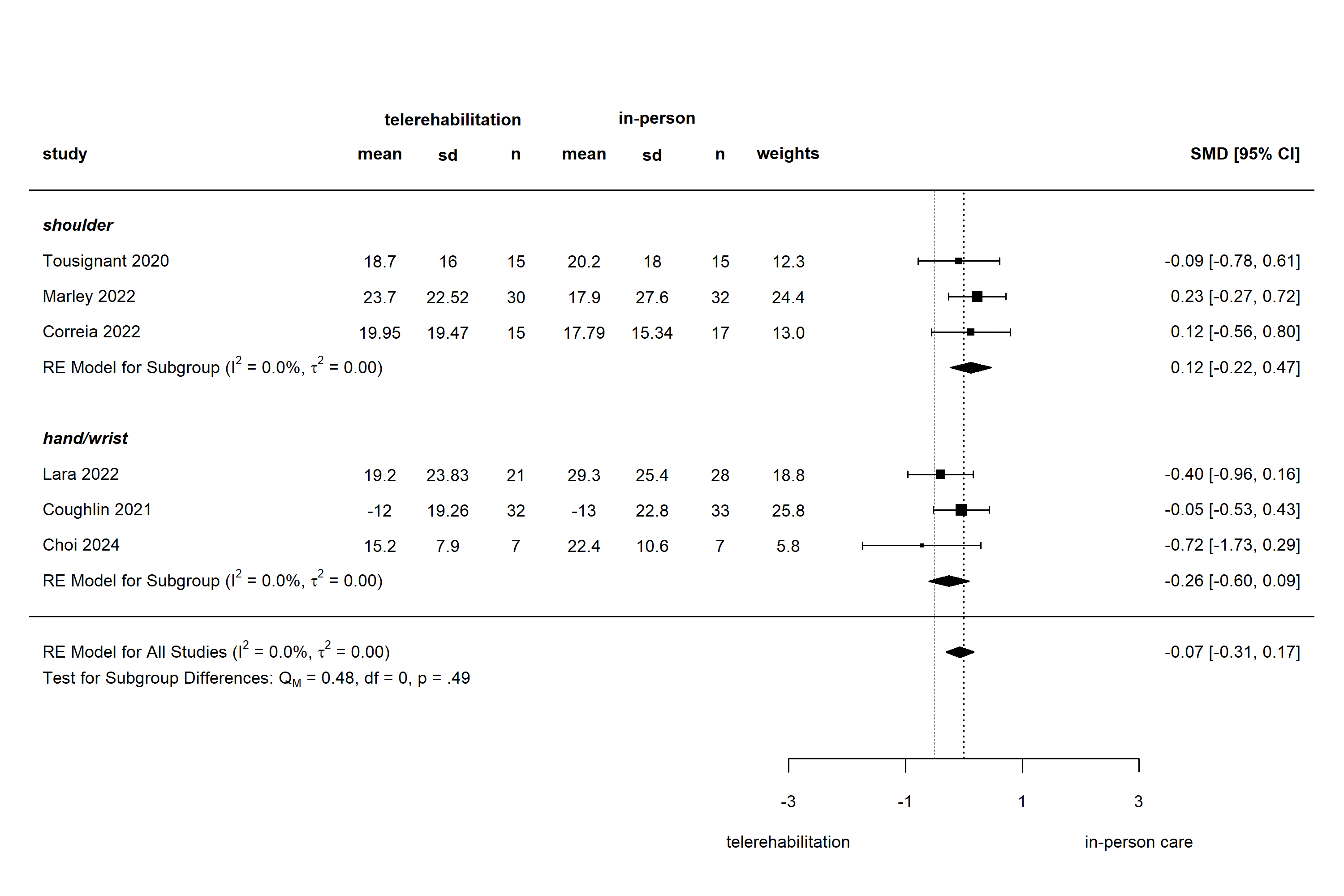


SMD: standardized mean difference, sd: standard deviation, CI: confidence interval, RE: random effects, DASH: disabilities of the arm, shoulder and hand questionnaire

### Subgroup analysis: telerehabilitation versus minimal care, overall Risk of Bias

Figure 14: forest plot subgroup analysis (QuickDASH/DASH), risk of bias


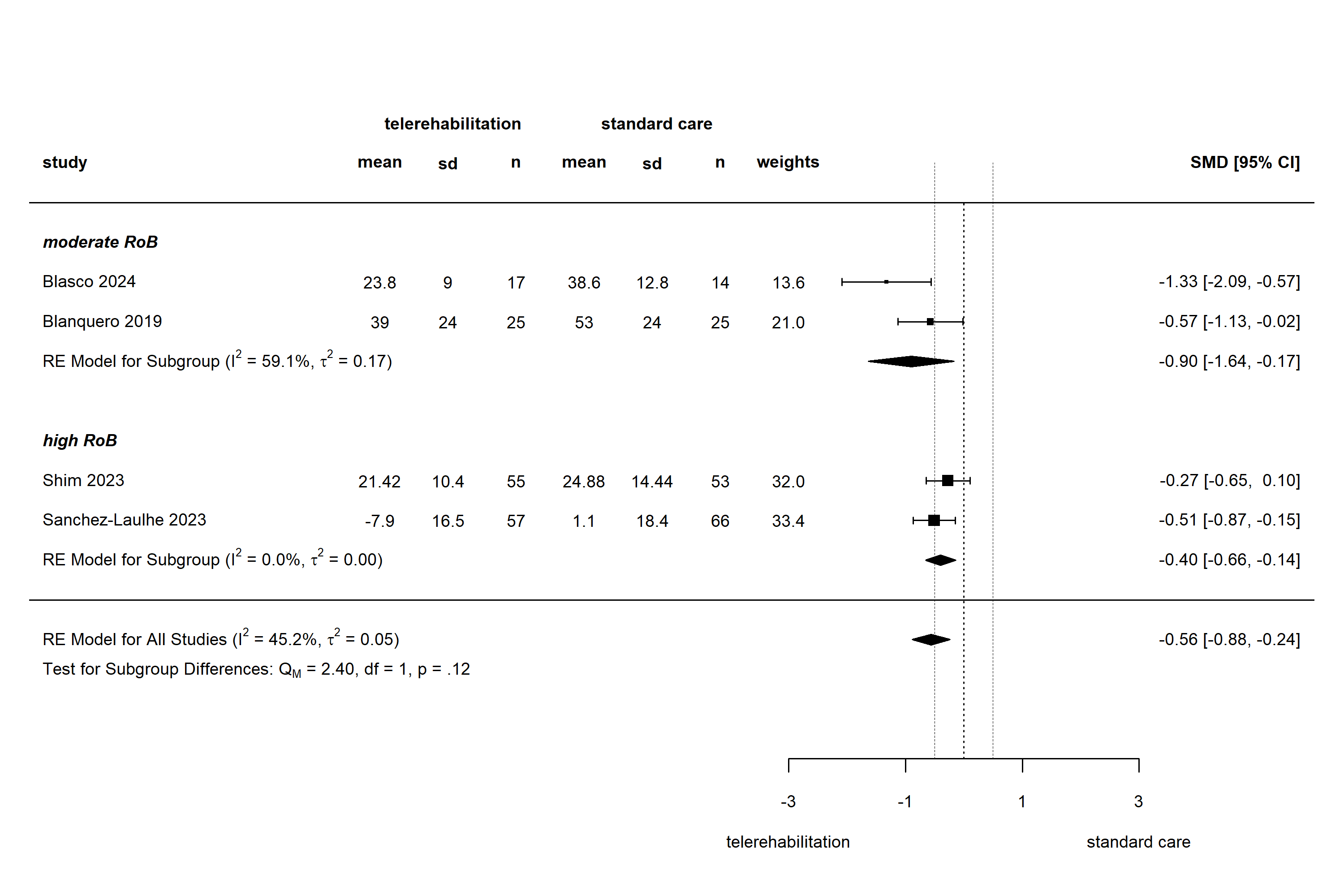


SMD: standardized mean difference, sd: standard deviation, CI: confidence interval, RoB: risk of bias, RE: random effects, DASH: disabilities of the arm, shoulder and hand questionnaire

### Subgroup analysis: telerehabilitation versus minimal care, duration of intervention >12 weeks

Figure 15: forest plot subgroup analysis (QuickDASH/DASH), intervention duration


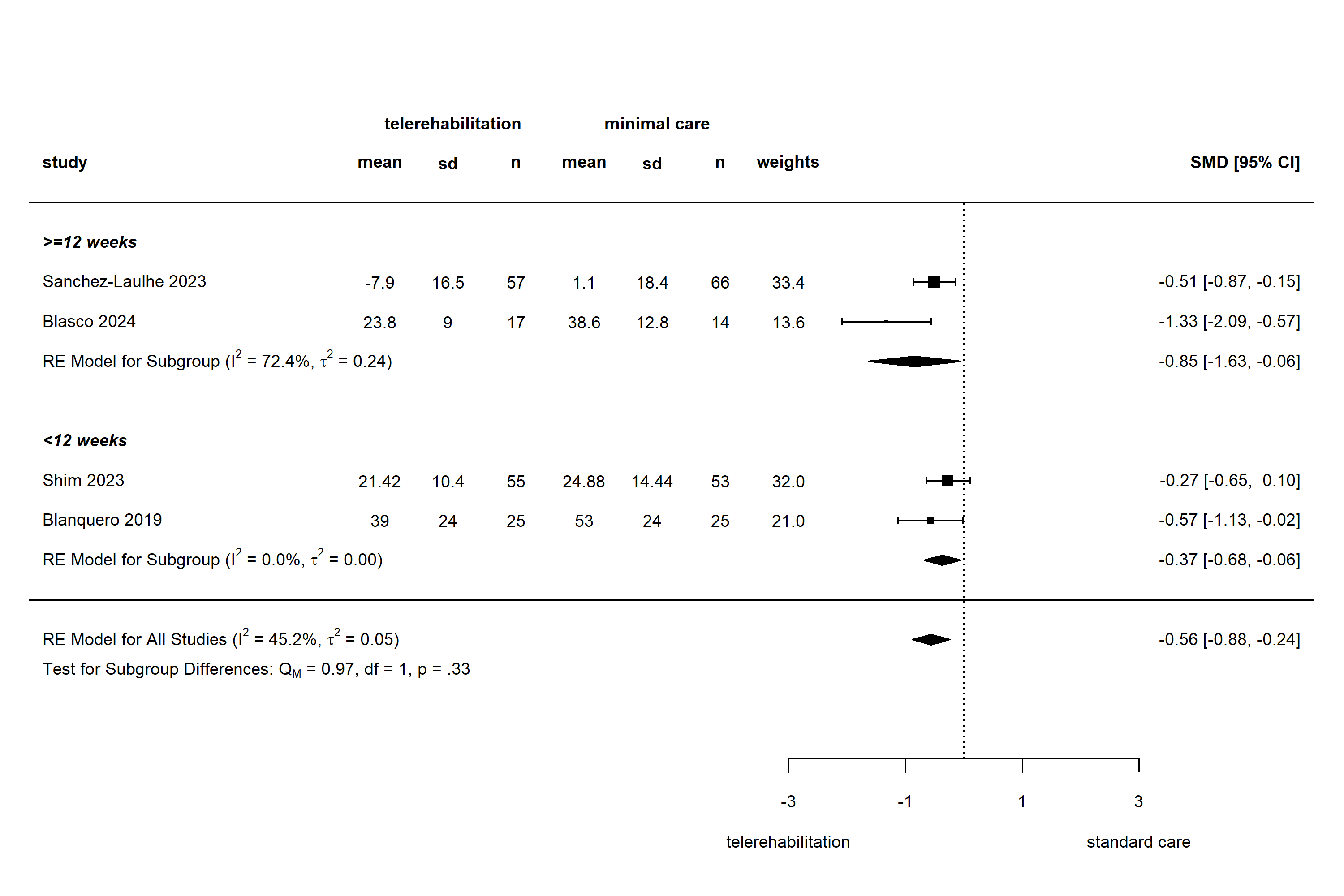


SMD: standardized mean difference, sd: standard deviation, CI: confidence interval, RE: random effects, DASH: disabilities of the arm, shoulder and hand questionnair

### Subgroup analysis: telerehabilitation versus minimal care, diagnosis

Figure 16: forest plot subgroup analysis (QuickDASH/DASH), diagnosis


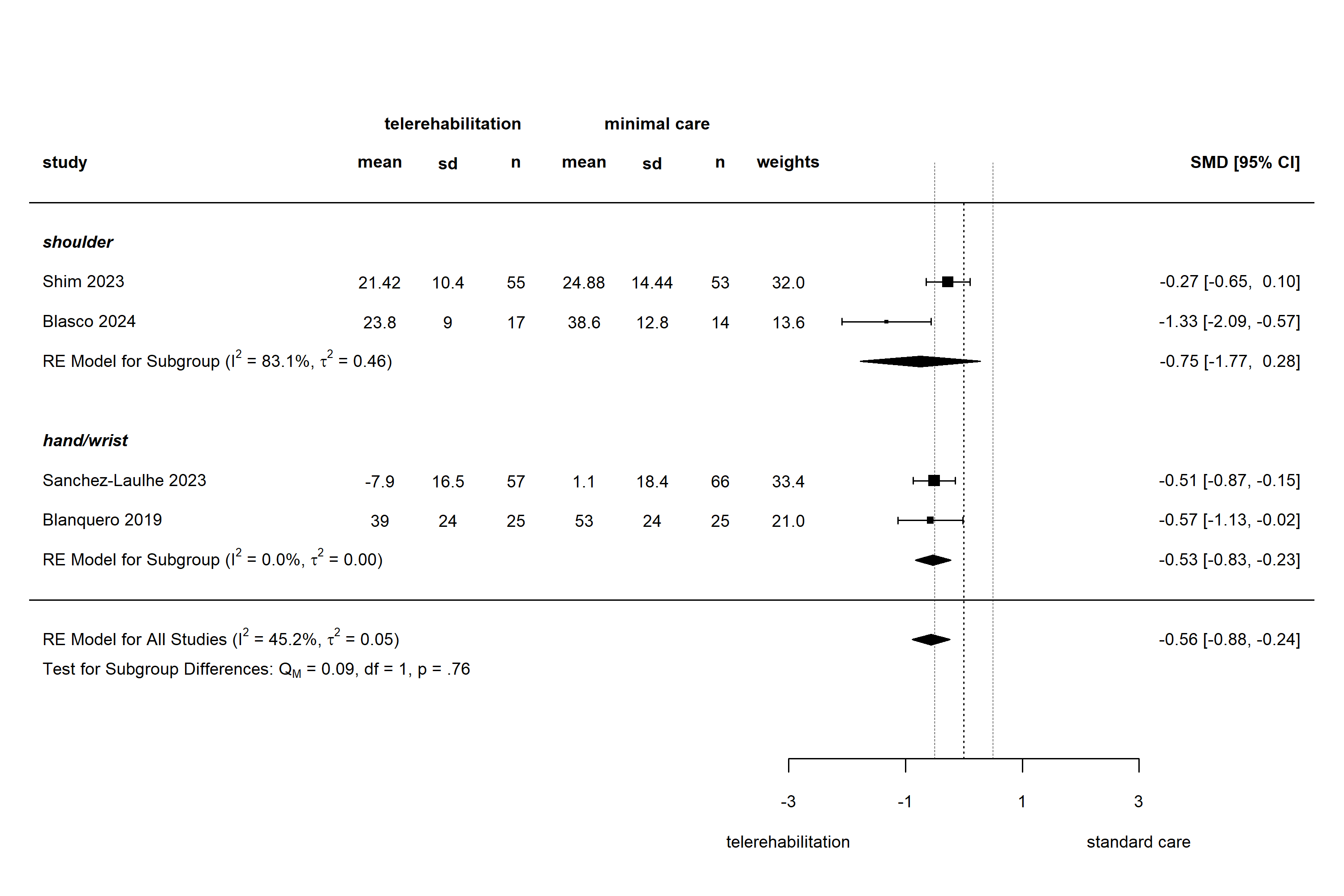


SMD: standardized mean difference, sd: standard deviation, CI: confidence interval, RE: random effects, DASH: disabilities of the arm, shoulder and hand questionnaire

### Sensitivity analysis: telerehabilitation versus minimal care, without Blasco

Figure 17: forest plot (QuickDASH/DASH), sensitivity analysis excluding small study


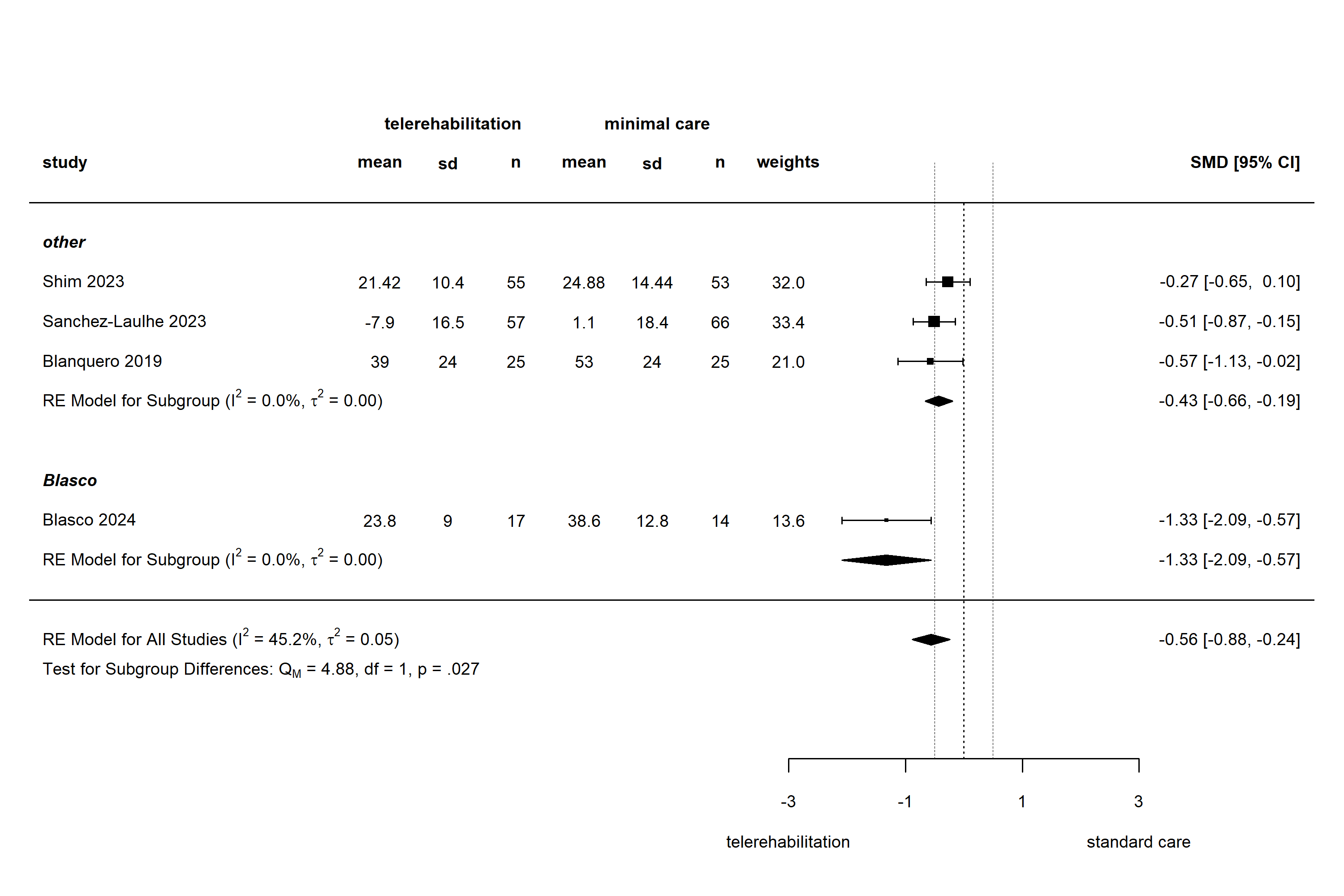


SMD: standardized mean difference, sd: standard deviation, CI: confidence interval, RE: random effects, DASH: disabilities of the arm, shoulder and hand questionnaire

## QuickDASH/DASH telerehabilitation as add on versus no add on

Figure 18: forest plot (QuickDASH/DASH), telerehabilitation as add-on versus no/minimal add-on


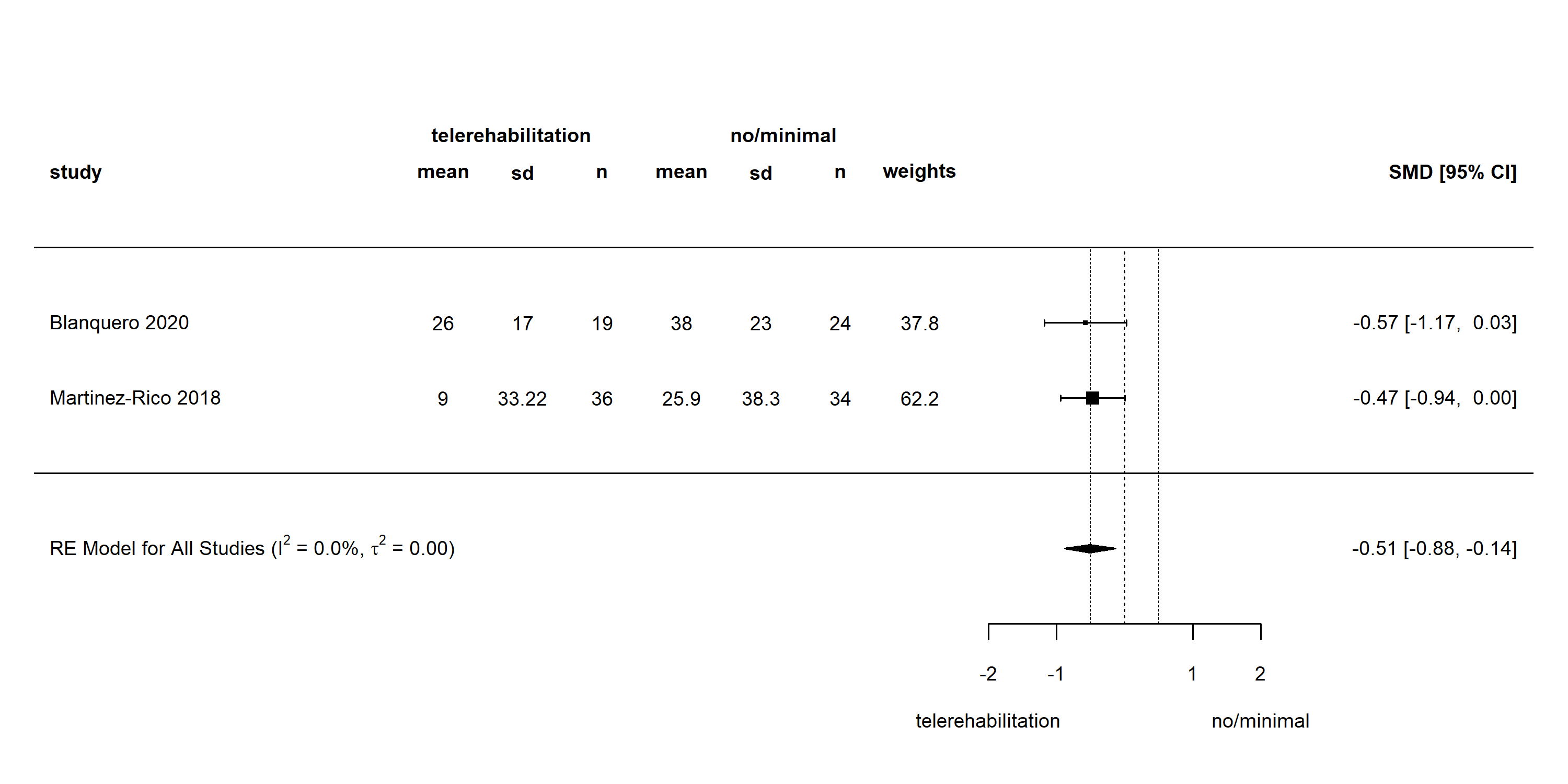


SMD: standardized mean difference, sd: standard deviation, CI: confidence interval, RE: random effects, DASH: disabilities of the arm, shoulder and hand questionnaire

## CMS telerehabilitation versus standard care

Figure 19: forest plot (CMS), telerehabilitation versus standard care


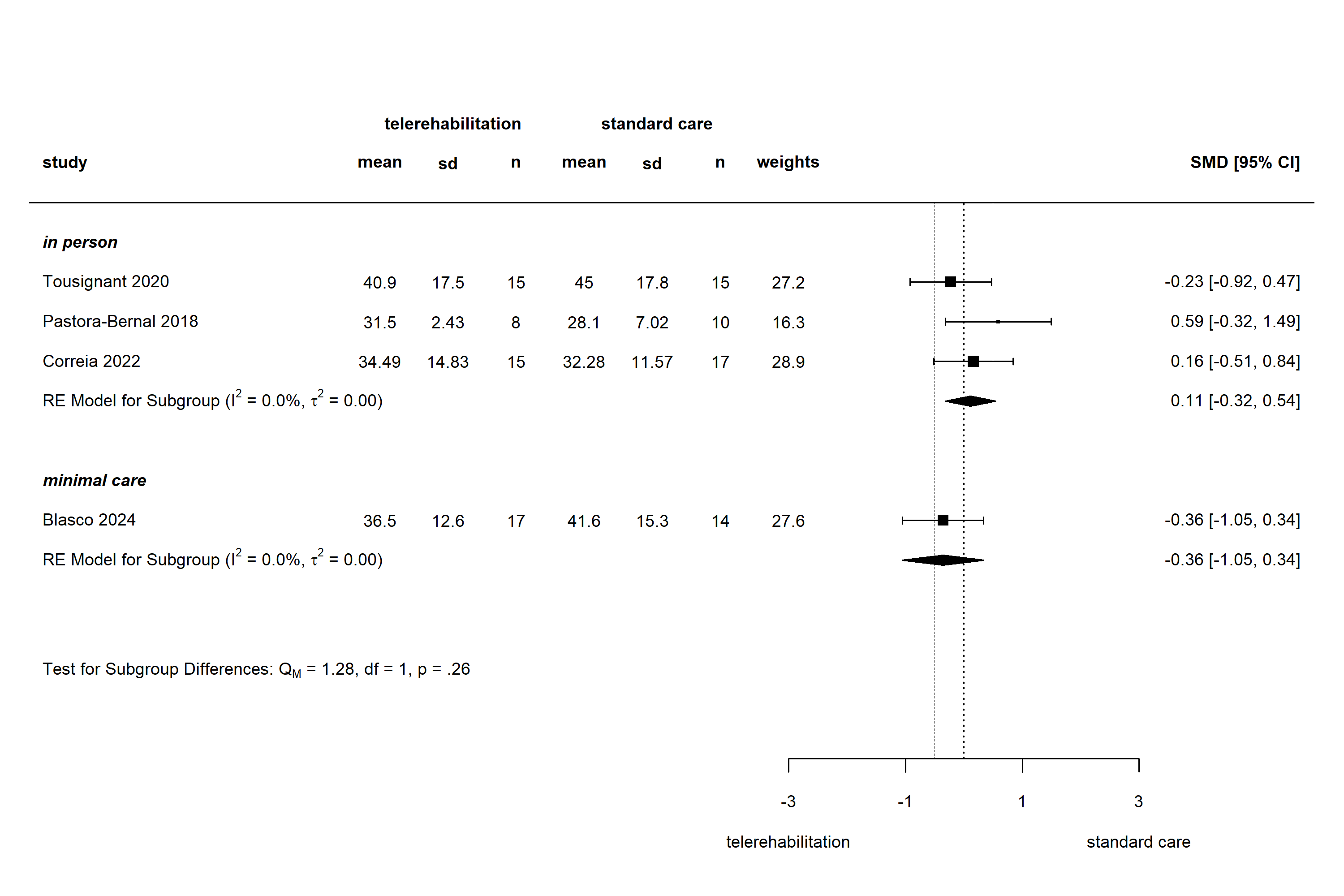


SMD: standardized mean difference, sd: standard deviation, CI: confidence interval, RE: random effects, CMS: Constant-Murley score

### Subgroup analysis: telerehabilitation versus in-person care, overall Risk of Bias

- No subgroup analysis of overall RoB 2 , since all three studies within in-person comparison are at moderate RoB and do not substantially differ for number of domains at risk

### Subgroup analysis: telerehabilitation versus in-person care, Intervention duration

Figure 20: forest plot subgroup analysis (CMS), intervention duration


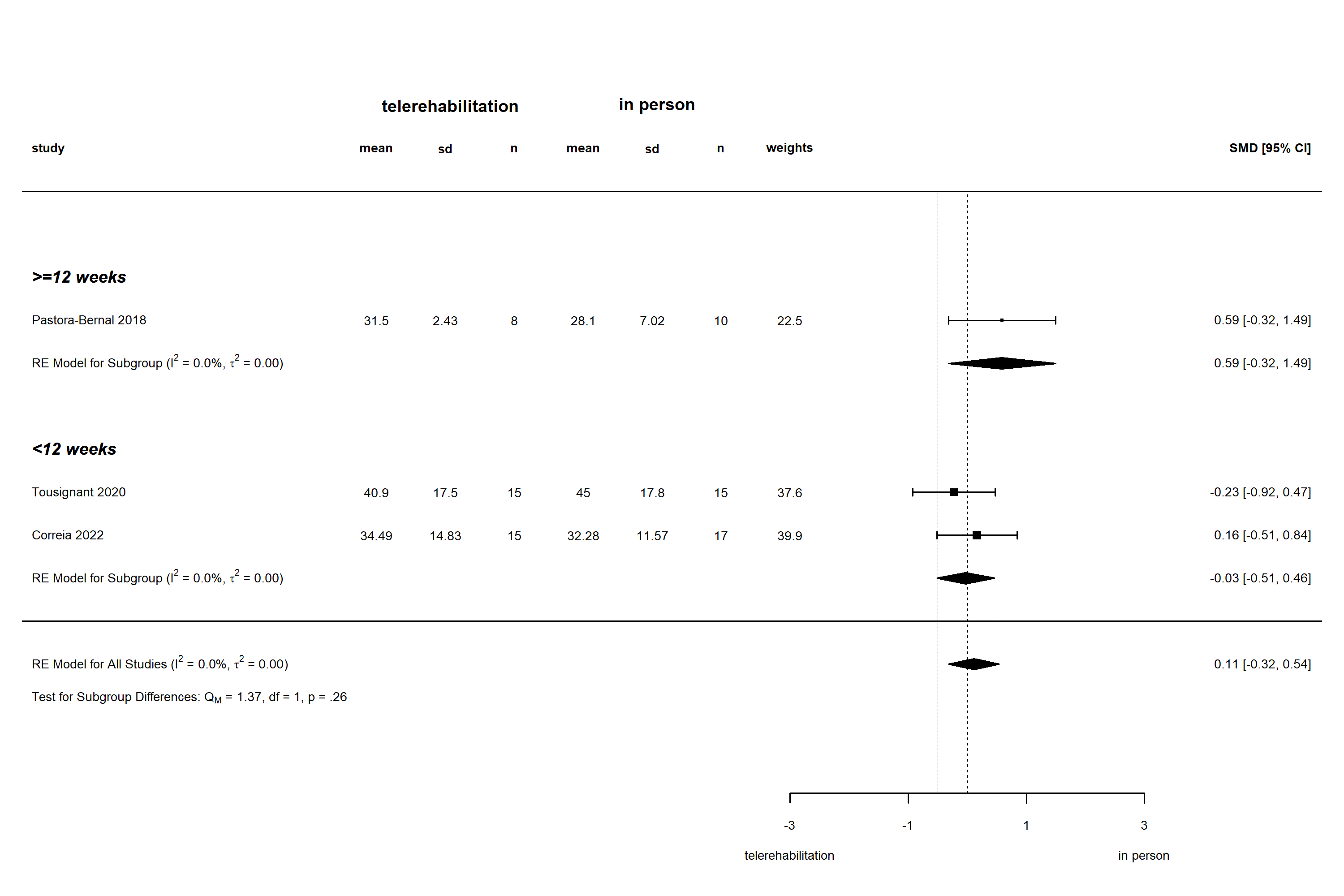


SMD: standardized mean difference, sd: standard deviation, CI: confidence interval, RE: random effects, CMS: Constant-Murley score

### Subgroup analysis: telerehabilitation versus in-person care, diagnosis

- Not applicable, all three studies examine shoulder diagnosis

## HrQol: EQ5D5L VAS/NRS telerehabilitation versus standard care

Figure 21: forest plot HrQol (EQ-5D-5L), telerehabilitation versus standard care


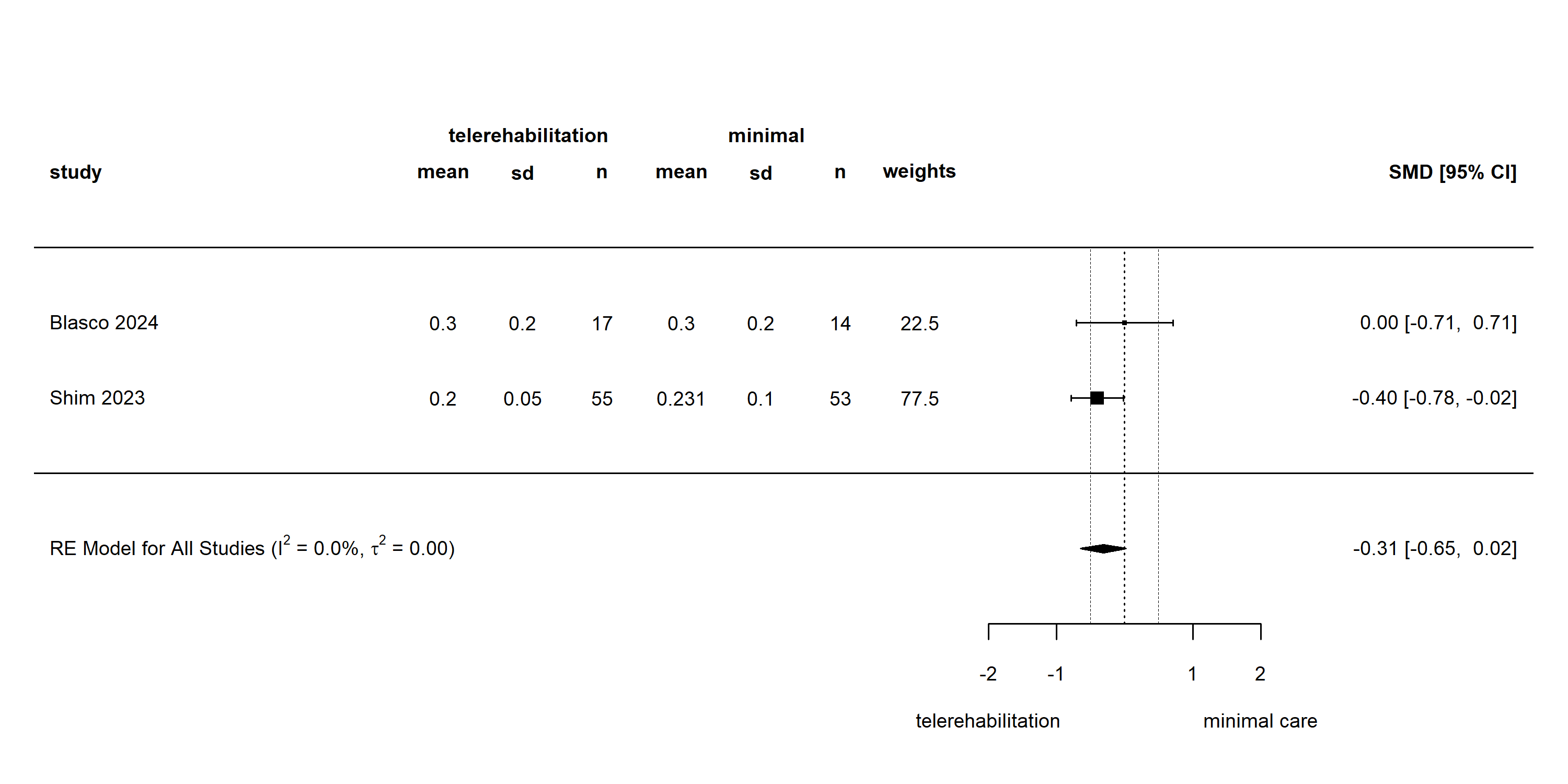


SMD: standardized mean difference, sd: standard deviation, CI: confidence interval, HrQol: Health-related quality of life RE: random effects, EQ-5D-5L: EuroQol five dimensions five levels measurement
