# Supplementary material for: The effectiveness of telerehabilitation in upper limb musculoskeletal disorders: a systematic review
Source: BMC Musculoskelet Disord. 2026 May 28;27:462. doi: 10.1186/s12891-026-10008-7 (PMC13220470; doi:10.1186/s12891-026-10008-7)
Supplement: Supplementary file 7 — Additional file 7: Results from studies reporting on follow up later than end of intervention. [file 12891_2026_10008_MOESM7_ESM.docx]

**Results from studies reporting on follow up later than end of intervention**

**Comparison 1: Telerehabilitation versus standard care (subgroup standard care = in-person rehabilitation)**

| **Study**  **(Country)** | **Population,**  **age mean (sd),**  **% female,**  **N: total (IG/CG),**  **setting** | **Intervention:**  **description (De), dose (Do), duration (Du), timing (T)** | **Control intervention: description (De), dose (Do), duration (Du), timing (T)** | **Time point from baseline** | **Instrument: name, scale, direction** | **Point estimates for groups mean (sd)*** | **Standardized effect measure**  **SMD [95% CI],**  **negative values favor telerehab^§^** |
| --- | --- | --- | --- | --- | --- | --- | --- |
| **Barrett et al., 2024**  **(USA)** | adults scheduled for primary thumb CMC arthroplasty with digital access, native speakers, exclusion of concomitant procedures  Age: 61.0 (9.5)  female: 86 %  N: 67 (31/36)  Single centre, urban clinic | De: video-based instruction to home exercise protocol  Do: 3 videos (4 minutes each), instructed exercise dosage same as CG  Du: 8 weeks  T: 4 weeks post op | De: in-person instruction to home exercise protocol  Do: 7 in-person sessions with hand therapist once weekly, 30 minutes per session, instructed exercise dosage same as IG  Du: 8 weeks  T: 4 weeks post op | 48 weeks | **ADL:**  PROMIS UE (0-100), higher is better | **ADL:**  IG: 42.5 (9.5)  CG: 40.5 (7.6) | **ADL:**  -0.23 [-0.76 to 0.31] |
| **Correia et al., 2022**  **(Portugal)** | adults with repair surgery after rotator cuff tear, complex tears and limiting comorbidities excluded  Age: 60.71 (6.9)  female: 78 %  N: 50 (27/23)  Single centre, urban clinic | De: guided exercise program using tablet app in combination with motion sensors providing real-time audio and video biofeedback during exercise sessions. Evaluation and weekly remote adjustment by the assigned therapist + home-based one-to-one physical therapy sessions.  Do: daily exercise with SWORD app (15-30 minutes) for 5 times/week, + 13 sessions in-person, 60 minutes/session  Du: 9 weeks  T: 3 weeks po | De: home-based rehabilitation provided by a physical therapist. Instruction to unsupervised exercising twice weekly.  Do: 30 sessions in-person, 3 times/week, 60 minutes/session + unsupervised exercising for at least 2 days/week  Du: 9 weeks T: 3 weeks po | 12 months | **Pain:**  CMS subscale pain (0-15), higher is better  **ADL:**  QuickDASH (0-100), lower is better  CMS total (0-100), higher is better | **Pain: median (sd)**  IG: 10 (10)  CG: 10 (10)  **ADL:**  QuickDASH:  IG: 20.6 (19.17)  CG: 28.75 (23.55)  CMS:  IG: 67.63 (11.51)  CG: 63.15 (14.92) | **pain:** median difference [95% CI]:  0 [0 to 5]  **ADL:**  QuickDASH:  -0.37 [-1.07 to 0.33]  CMS:  -0.32 [-1.02 to 0.37] |
| **Coughlin et al., 2021**  **(United Kingdom)** | adults with nonoperatively treated distal radius fractures, exclusion if unable to access video  Age: 49  female: 68 %  N: 80 (40/40)  Single centre, urban clinic | De: instruction for exercise through videos  Do: 4 instruction videos, self-guided exercising for 6 weeks  Du: 6 weeks  T: five to seven weeks post- injury | De: face-to face instruction to self-guided exercising  Do: In-person sessions with instruction to exercises  Du: 6 weeks  T: five to seven weeks post- injury | 12 months | **ADL:**  DASH (0-100), lower is better | **ADL:**  Change in DASH:  IG: 5 (12)  CG: 5 (11) | **ADL:**  Change in DASH:  0.00 [-0.54 to 0.54] |
| **Roddey et al., 2002**  **(USA)** | Adults (35-78) with rotator cuff tear, operative treatment  Age: 58  female: 36 %  N: 108 (54/54)  Single centre, urban clinic | De: instruction to self-guided exercising via three videos  Do: Three videos  Du: 24 weeks  T: Unclear | De: in-person instruction to self-guided exercising  Do: 4 in-person instruction sessions 15 minutes  Du: 24 weeks  T: Unclear | 52 weeks | **ADL:**  SPADI (0-100), lower is better  Penn Score (0-100), higher is better | **ADL:**  SPADI:  IG: 12.3 (14.3)  CG: 12.4 (14.4)  Penn Score:  IG: 85.6 (13.8)  CG: 85.9 (16.7) | **ADL:**  SPADI:  -0.01 [-0.48 to 0.47]  Penn Score:  0.02 [-0.46 to 0.50] |

SMD: Standardized mean difference, CI: confidence interval, sd: standard deviation, IG: intervention group, CG: control group, CMC: carpometacarpal, ADL: activities of daily living, HrQol: health-related quality of life, VAS: visual analogue scale, NRS: numeric rating scale, CMS: Constant-Murley score, DASH: disabilities of the arm, shoulder and hand questionnaire, PROMIS UE: patient reported outcomes measurement information system upper extremity, SPADI: shoulder pain and disability index, Penn score: Pennsylvania shoulder score

*data are presented from values of endpoint measures, if reported otherwise, data details are presented, ^§^if no SMD could be derived other effect estimates are presented with details

**Comparison 1: Telerehabilitation versus standard care (subgroup standard care = minimal rehabilitation)**

| **Study**  **(Country)** | **Population,**  **age mean (sd),**  **% female,**  **N: total (IG/CG),**  **setting** | **Intervention:**  **description (De), dose (Do), duration (Du), timing (T)** | **Control intervention: description (De), dose (Do), duration (Du), timing (T)** | **Time point from baseline** | **Instrument: name, scale, direction** | **Point estimates for groups mean (sd)*** | **Standardized effect measure**  **SMD [95% CI],**  **negative values favor telerehab^§^** |
| --- | --- | --- | --- | --- | --- | --- | --- |
| **Meijer et al., 2024**  **(Netherlands)** | Adults (18 or older) with distal radius fracture, operative or conservative treatment, who own compatible smartphone or tablet, language barriers to questionnaires  Age mean (IQR): 52 (42-65)  female: 82.8 %  N: 93 (47/46)  three regional hospitals, one academic hospital | De: smartphone or tablet-based exergaming. Upon request referral to hand-therapist  Do: Exergaming three to five times per day, for 10–15 min.  Du: 6 weeks  T: 3-5 days po or after cast removal | De: home-based unsupervised wrist exercises. Upon request or recommendation by clinician referral to hand-therapist  Do: Instruction to perform the exercises three to five times per day, for 10–15 min.  Du: 6 weeks  T: 3-5 days po or after cast removal | 12 weeks | **pain:**  NRS, (0-10), lower is better  **ADL:**  PRWE (0-100), lower is better | **pain:**  only reported visually: at week 12 endpoint values for control were lower (e.g. better) than intervention group but confidence intervals are overlapping  **ADL: mean [95% CI]**  Change in PRWE (higher is better) [95% CI]  IG: 23.9 [18.5 to 29.4]  CG: 24.8 [18.2 to 31.4] | **pain:**  -  **ADL:**  Change in PRWE:  0.04 [-0.36 to 0.45] |
| **Rodríquez-Sánchez-Laulhé et al., 2023**  **(Spain)** | Adults (18+) diagnosed with hand osteoarthritis, own smartphone or tablet with internet access, comorbidities excluded  Age: 63  female: 67.5 %  N Participants: 74 (34/40)  N hands: 144 (66/78)  two rural community health centres | De: home exercise with tablet app (diary function, self-management recommendations, exercise instructions, symptom report); monthly follow-up phone calls  Do: 15-20 min 4 times/week  Du: 12 weeks  T: NA | De: paper-based home exercise program. Introductory face-to-face session to explain the exercise program. Monitoring telephone calls once a month  Do: Instruction to exercise 15-20 min 4 times/week  Du: 12 weeks  T: NA | 24 weeks | **pain:**  NRS (0-10), lower is better  AUSCAN pain (0-25), lower is better  **ADL:**  QuickDASH (0-100), lower is better  AUSCAN  (0-75), lower is better | **pain:**  change in NRS:  IG: 0.0 (2.6)  CG: 0.4 (3.5)  Change in AUSCAN pain:  IG: -2.0 (4.6)  CG: -1.1 (5.4)  **ADL:**  Change in QuickDASH, lower is better:  IG: -8.9 (18.2)  CG: -1.2 (28.0)  Change in AUSCAN  IG: -5.3 (11.7)  CG: -2.1 (14.3) | **pain:**  change in NRS:  -0.13 [-0.48 to 0.23]  Change in AUSCAN pain:  -0.18 [-0.53 to 0.18]  **ADL:**  Change in QuickDASH:  -0.32 [-0.68 to 0.04]  Change in AUSCAN:  -0.24 [-0.60 to 0.11] |
| **Shim et al., 2023**  **(Republic of Korea)** | Adults (50+) with rotator cuff tear, operative treatment, limiting comorbidities excluded  Age (sd): 64  female: 61 %  N: 115 (58/57)  Single centre, urban clinic | De: brochure-based exercise for 6 weeks followed by AR-based exercises for 6 weeks with real-time feedback on exercises and session performance, feedback at 6 and 12 weeks by physician.  Do: instruction to perform 3–5 sets of exercises with 10 repetitions of each set per day  Du: 6 weeks  T: Few days po (brochure-based exercises)  6 weeks: AR exercising | De: brochure-based home exercises, exercise diary, condition checked by weekly telephone call.  Do: instruction to perform 3–5 sets of exercises with 10 repetitions of each set per day  Du: 6 weeks  T: Few days po  (brochure-based exercises) | 24 weeks | **pain:**  NRS (0-10), lower is better  **ADL:**  SST, 0-12, higher is better  SPADI (0-100), lower is better  DASH (0-100), lower is better  **HrQol:**  EQ-5D-5L  (0-1), higher is better | **pain:**  IG: 2.35 (1.88)  CG: 2.25 (1.80)  **ADL:**  SST:  IG: 8.93 (2.68)  CG: 8.68 (3.16)  SPADI:  IG: 11.94 (10.08)  CG: 15.18 (12.66)  DASH:  IG: 11.08 (7.45)  CG: 14.75 (11.75)  **HrQol:**  IG: 0.833 (0.053)  CG: 0.806 (0.091) | **pain:**  0.05 [-0.32 to 0.43]  **ADL:**  SST:  -0.07 [-0.45 to 0.30]  SPADI:  -0.28 [-0.66 to 0.10]  DASH:  -0.37 [-0.75 to 0.01]  **HrQol:**  -0.36 [-0.74 to 0.02] |

SMD: Standardized mean difference, CI: confidence interval, sd: standard deviation, IG: intervention group, CG: control group, CMC: carpometacarpal, ADL: activities of daily living, HrQol: health-related quality of life, VAS: visual analogue scale, NRS: numeric rating scale, CMS: Constant-Murley score, DASH: disabilities of the arm, shoulder and hand questionnaire, EQ‑5D‑5L: EuroQol five dimensions five levels measurement, SST: simple shoulder test, SPADI: shoulder pain and disability index, PRWE: patient-rated wrist evaluation, AUSCAN: Australian/Canadian osteoarthritis hand index

*data are presented from values of endpoint measures, if reported otherwise, data details are presented, ^§^if no SMD could be derived other effect estimates are presented with details

**Comparison 2: Telerehabilitation as add-on to standard care versus standard care without/minimal add-on**

| **Study**  **(Country)** | **Population,**  **age mean (sd),**  **% female,**  **N: total (IG/CG),**  **setting** | **Intervention:**  **description (De), dose (Do), duration (Du), timing (T)** | **Control intervention: description (De), dose (Do), duration (Du), timing (T)** | **Time point from baseline** | **Instrument: name, scale, direction** | **Point estimates for groups mean (sd)*** | **Standardized effect measure**  **SMD [95% CI],**  **negative values favor telerehab^§^** |
| --- | --- | --- | --- | --- | --- | --- | --- |
| **Martinez-Rico et al., 2018**  **(Spain)** | Adults with shoulder instability and Bankart repair  Age: 28  female: 23 %  71 (36/35)  Single centre | De: 3 weeks of out-patient physical therapy with home exercise program + phone-based coaching to self-care  Do: + 3 phone calls/week during first month  Du: 4 weeks  T: 3-4 weeks po (at sling removal) | De: 3 weeks of out-patient physical therapy with home exercise program  Do: Same outpatient rehabilitation as IG  Du: 4 weeks  T: 3-4 weeks po (at sling removal) | 6 months  12 months | **pain:**  VAS (0-10), lower is better  **ADL:**  DASH (0-100), lower is better  OSIS (12-60), lower is better  **pain:**  VAS (0-10), lower is better  **ADL:**  DASH (0-100), lower is better  OSIS (12-60), lower is better  Rowe (0-100), higher is better | **pain:**  IG: 0.3  CG: 2.2  P between: 0.01  **ADL:**  DASH:  IG: 2.9  CG: 9.7  P between: 0.07  OSIS:  IG: 14.5  CG: 21.6  P between: 0.004  **pain**:  IG: 0.2  CG: 1.2  p-between: 0.051  **ADL**:  DASH:  IG: 0.9  CG: 5.0  P between: 0.181  OSIS:  IG: 12.5  CG: 17.2  P between: 0.013  Rowe:  IG: 93.4  CG: 89.1  P between: 0.21 | **pain:**  -0.63 [-1.11 to ‑0.15]  **ADL:**  DASH:  -0.44 [-0.91 to 0.04]  OSIS:  -0.70 [-1.19 to -0.22]  **pain**:  -0.47 [-0.94 to 0.01]  **ADL**:  DASH:  -0.32 [-0.79 to 0.15]  OSIS:  -0.60  [-1.08 to -0.12]  Rowe:  -0.30 [-0.77 to 0.17] |

SMD: Standardized mean difference, CI: confidence interval, sd: standard deviation, IG: intervention group, CG: control group, CMC: carpometacarpal, ADL: activities of daily living, VAS: visual analogue scale, DASH: disabilities of the arm, shoulder and hand questionnaire, OSIS: Oxford shoulder instability score

*data are presented from values of endpoint measures, if reported otherwise, data details are presented, ^§^if no SMD could be derived other effect estimates are presented with details
