# Supplementary material for: The effectiveness of telerehabilitation in upper limb musculoskeletal disorders: a systematic review
Source: BMC Musculoskelet Disord. 2026 May 28;27:462. doi: 10.1186/s12891-026-10008-7 (PMC13220470; doi:10.1186/s12891-026-10008-7)
Supplement: Supplementary file 8 — Additional file 8: Characteristics and results of Non-Randomized Studies of Interventions (NRSIs). [file 12891_2026_10008_MOESM8_ESM.docx]

**Characteristics and results of Non-Randomized Studies of Interventions (NRSIs)**

**Telerehabilitation versus standard care (subgroup standard care = in-person care)**

| **Study**  **(Country)** | **Population,**  **age mean (sd),**  **% female,**  **N: total (IG/CG),**  **setting** | **Intervention:**  **description (de), dose (do), duration (du), timing (t)** | **Control intervention:**  **description (de), dose (do), duration (du), timing (t)** | **Time point from baseline** | **Instrument: name, scale, direction** | **Point estimates for groups mean (sd)*** | **Standardized effect measure**  **SMD [95% CI],**  **negative values favor telerehab^§^** |
| --- | --- | --- | --- | --- | --- | --- | --- |
| **Palm et al., 2023**  **(USA)** | patients undergoing thumb carpometacarpal arthroplasty (thumb cohort) or reverse total shoulder arthroplasty (shoulder cohort)  Age (median (IQR):  IG: 61 (56-66); CG: 70 (64-74) (thumb cohort)  IG: 66.7 (66-70); CG: 71.9 (69-76)  (shoulder cohort)  Female: 83% (thumb cohort), 89 % (shoulder cohort)  N: 30 (19/11) (thumb cohort)  19 (5/14) (shoulder cohort)  Single centre, urban clinic | De: alternating in-person and videoconferencing visits (physical therapy/occupational therapy)  Do: 5 tele-sessions and 5 in-person sessions, one session per week (week 1-8), one session every 2 weeks (week 9-12)  Du: 12 weeks  T: 2 weeks po | De: in-person (physical therapy/occupational therapy), same content as IG  Do: 10 in-person sessions one session per week (week 1-8), one session every 2 weeks (week 9-12)  Du: 12 weeks  T: 2 weeks po | 12 weeks  24 weeks | **Pain:**  VAS (0-10), lower is better  **ADL:**  QuickDASH,  0-100, lower is better  ASES, 0-100, higher is better  **HrQol:**  VR-12 physical  **Pain:**  VAS (0-10), lower is better  **ADL:**  QuickDASH,  0-100, lower is better | **Pain:**  Median (IQR) (thumb)  IG: 2 (0,3)  CG: 1 (0,3)  Median (IQR) (shoulder)  IG: 0 (0,0.5)  CG: 0 (0,1.75)  **ADL:**  QuickDASH, Median (IQR) (thumb)  IG: 28 (19,40)  CG: 50 (31,56)  QuickDASH, Median (IQR) (shoulder)  IG: 25 (23,48)  CG: 57 (53,60)  ASES, Median (IQR) (shoulder)  IG: 71 (64,78)  CG: 54 (37,63)  **HrQol:**  Median (IQR) (shoulder)  IG: 45 (44,48)  CG: 37 (35,39)  **Pain:**  Median (IQR) (thumb)  IG: 0 (0,2)  CG: 0 (0,2)  Median (IQR) (shoulder)  IG: 0 (0,0)  CG: 2 (1,3.5)  **ADL:**  Median (IQR) (thumb)  IG: 11 (5,18)  CG: 34 (17,41) | **Pain:**  Only p value provided  p = 1.0 (thumb)  p = 0.85 (shoulder)  **ADL:**  p = 0.24 (thumb)  p = 0.8 (shoulder)  ASES  p = 0.51 (shoulder)  **HrQol:**  p = 0.23  **Pain:**  p = 1.0 (thumb)  p = 0.6 (shoulder)  **ADL:**  p = 0.55 (thumb) |
| **Mayer et al., 2021**  **(Israel)** | Patients with operative treatment following elbow fracture  Age (median (IQR)):  IG: 33 (27.9); CG: 60 (37.0)  female: 61 %  N: 18 (9/9)  NA | De: occupational therapy with blended in-person and tele-sessions, home exercise using app connected to sensory system  Do: 1-2 in-person sessions + 1-2 telerehabilitation sessions, 30 minutes each+ home exercise  Du: 4 weeks  T: immediately post-operative | De: occupational therapy via in-person sessions, instruction to home exercise  Do: 3-4 in-person sessions, 30 minutes each+ home exercise  Du: 4 weeks  T: immediately post-operative | 4 weeks  12 weeks  12 months | **ADL:**  Change in DASH, 0-100 (%), higher is better  Change in PREE, 0-100 (%), higher is better  Change in JHFT, 0-100 (%), higher is better  **ADL:**  Change in DASH, 0-100 (%), higher is better  Change in PREE, 0-100 (%), higher is better  Change in JHFT, 0-100 (%), higher is better  **ADL:**  Change in DASH, 0-100 (%), higher is better  Change in PREE, 0-100 (%), higher is better  Change in JHFT, 0-100 (%), higher is better | **ADL:**  Change in DASH % (IQR)  IG: 52 (33.17)  CG: 35.3 (58.0)  Change in PREE % (IQR)  IG: 60.4 (48.8)  CG: 7.2 (52.4)  Change in JHFT % (IQR)  IG: 15.4 (46.3)  CG: 28.0 (22.9)  **ADL:**  Change in DASH % (IQR)  IG: 59.1 (49.6)  CG: 53.6 (58.8)  Change in PREE % (IQR)  IG: 42.9 (58.4)  CG: 58.8 (67.03)  Change in JHFT % (IQR)  IG: 32.1 (26.1)  CG: 24.6 (37.7)  **ADL:**  Change in DASH % (IQR)  IG: 75.0 (50.9)  CG: 94.7 (30.3)  Change in PREE % (IQR)  IG: 80 (65.16)  CG: 70.4 (44.7)  Change in JHFT % (IQR)  IG: 38.9 (31.8)  CG: 43.3 (58.8) | no between-group statistically significant differences for each evaluation timepoint, bayes factor BF01:  DASH (BF01 range: 1.314–1.957)  PREE (BF01 range: 0.949–2.415)  JHFT (BF01 range: 1.921–2.178) |

SMD: Standardized mean difference, CI: confidence interval, IG: intervention group, CG: control group, IQR: inter quartile range, ADL: activities of daily living, HrQol: health-related quality of life, VAS: visual analogue scale, NRS: numeric rating scale, CMS: Constant-Murley score, DASH: disabilities of the arm, shoulder and hand questionnaire, ASES score: American shoulder and elbow surgeons score, VR-12: Veterans RAND 12-item health survey, PREE: The patient-rated elbow evaluation, JHFT: Jebsen hand function test

*Data are presented from values of endpoint measures, if reported otherwise, data details are presented, ^§^if no SMD could be derived other effect estimates are presented with details

**Telerehabilitation versus standard care (subgroup standard care = minimal rehabilitation)**

| **Study**  **(Country)** | **Population,**  **age mean (sd),**  **% female,**  **N: total (IG/CG),**  **setting** | **Intervention:**  **description (de), dose (do), duration (du), timing (t)** | **Control intervention:**  **description (de), dose (do), duration (du), timing (t)** | **Time point from baseline** | **Instrument: name, scale, direction** | **Point estimates for groups mean (sd)*** | **Standardized effect measure**  **SMD [95% CI],**  **negative values favor telerehab^§^** |
| --- | --- | --- | --- | --- | --- | --- | --- |
| **Chen et al., 2020**  **Taiwan** | Patients with confirmed diagnosis of adhesive capsulitis,  patients  Age: 55  female: 36 %  N: 15 (8/7)  Single centre | De: app-based home-exercise program with monitoring function, monthly supervision of progression with physician  Do: daily exercise 10 times a day  Du: 12 weeks  T: NA | De: instruction to home-based exercises along with education on disease, recommendations on pain management and ADL behavior, monthly supervision and progression of exercise with physician  Do: daily exercise 10 times a day  Du: 12 weeks  T:NA | 12 weeks | **pain:**  VAS (0-10), lower is better  **ADL:**  QuickDASH  (0-100), lower is better | **pain:**  IG: 2.0 (0.6)  CG: 3.3 (1.1)  **ADL:**  IG: 9.8 (12.4)  CG: 19.1 (13.7) | **pain:**  beta (SE), group*time: -0.43 (0.70)  **ADL:**  beta (SE), group*time: -16.60 (5.97) |

SMD: Standardized mean difference, CI: confidence interval, IG: intervention group, CG: control group, SE: standard error, ADL: activities of daily living, HrQol: health-related quality of life, VAS: visual analogue scale, DASH: disabilities of the arm, shoulder and hand questionnaire

*Data are presented from values of endpoint measures, if reported otherwise, data details are presented, ^§^if no SMD could be derived other effect estimates are presented with details

**Telerehabilitation as add-on to standard care versus standard care without/minimal add-on**

| **Study**  **(Country)** | **Population,**  **age mean (sd),**  **% female,**  **N: total (IG/CG),**  **setting** | **Intervention:**  **description (de), dose (do), duration (du), timing (t)** | **Control intervention:**  **description (de), dose (do), duration (du), timing (t)** | **Time point from baseline** | **Instrument: name, scale, direction** | **Point estimates for groups mean (sd)*** | **Standardized effect measure**  **SMD [95% CI],**  **negative values favor telerehab^§^** |
| --- | --- | --- | --- | --- | --- | --- | --- |
| **Suero-Pineda et al., 2023 a & b**  **(Spain)** | Patients with trauma or soft injuries of the wrist, hand and fingers  Age: 51.5  female: 67.1 %  N: 663 (270/393)  Four hospitals | De: home exercise via tablet app + in-person physical therapy if needed  Do: Home exercise 20-25 minutes/day, 5 days/week; In-person PT only if needed  Du: 4 weeks or longer  T: splint removal/post-operative clearance | De: instruction to home exercise + in-person physical therapy if needed  Do: Home exercise 20-25 minutes/day, 5 days/week; In-person PT only if needed  Du: 4 weeks or longer  T: splint removal/post-operative clearance | 4 weeks  12 weeks | **pain:**  VAS (0-10), lower is better  **ADL:**  QuickDASH  (0-100), lower is better  PRWE (0-100), lower is better  **pain:**  VAS (0-10), lower is better  **ADL:**  QuickDASH  (0-100), lower is better  PRWE (0-100), lower is better | **pain:**  IG: 4.32 (2.49)  CG: 5.09 (2.39)  **ADL:**  QuickDASH  IG: 42.01 (21.61)  CG: 49.1 (24.71)  PRWE  IG: 48.69 (23.89)  CG: 57.37 (22.91)  **pain:**  IG: 2.91 (2.74)  CG: 3.94 (2.98)  **ADL:**  QuickDASH  IG: 26.5 (25.5)  CG: 36.01 (26.69)  PRWE  IG: 28.97 (22.94)  CG: 43.89 (28.27) | Results from ANOVA group*time:  **pain:**  n² = 0.038, p = 0.001  **ADL:**  QuickDASH  n²= 0.067, p = 0.001  PRWE:  n² = 0.066, p = 0.01 |

SMD: Standardized mean difference, CI: confidence interval, IG: intervention group, CG: control group, SE: standard error, ADL: activities of daily living, HrQol: health-related quality of life, VAS: visual analogue scale, DASH: disabilities of the arm, shoulder and hand questionnaire, PRWE: patient-rated wrist evaluation

*Data are presented from values of endpoint measures, if reported otherwise, data details are presented, ^§^if no SMD could be derived other effect estimates are presented with details
